# IGF2BP1-Mediated m⁶A Modification Stabilizes HMGA2 mRNA to Promote Intrahepatic Cholangiocarcinoma Progression

**DOI:** 10.1016/j.jcmgh.2026.101837

**Published:** 2026-06-24

**Authors:** Yu Guo, Le Zhu, Kaiheng Hu, Xiangyu Wang, Sanfeng Han, Meiping Ni, Jixuan Chen, Keyu Shen, Qimeng Chang, Chengjie Zhang, Mingxiu Cai, Hong Cheng, Chaoqun Wang, Haoting Sun, Qiongzhu Dong

**Affiliations:** 1 Department of General Surgery, Hepatobiliary Surgery Center, Huashan Hospital and Cancer Metastasis Institute, Fudan University, Shanghai, China; 2 Key Laboratory of Whole-Period Monitoring and Precise Intervention of Digestive Cancer, Shanghai Municipal Health Commission, Minhang Hospital, Fudan University, Shanghai, China; 3 Queen Mary School, Jiangxi Medical College, Nanchang University, Nanchang, Jiangxi, China; 4 College of Acupuncture-Moxibustion, Anhui University of Chinese Medicine, Hefei, Anhui, China; 5 Department of Medical Oncology, National Clinical Research Center for Cancer/Cancer Hospital, National Cancer Center, Chinese Academy of Medical Sciences and Peking Union Medical College, Beijing, China

**Keywords:** HMGA2, IGF2BP1, Intrahepatic Cholangiocarcinoma, N6-Methyladenosine

## Abstract

**Background & Aims:**

Intrahepatic cholangiocarcinoma (iCCA) remains a lethal malignancy with a lack of effective therapies, underscoring the critical need to identify novel therapeutic targets. The high-mobility group protein A2 (HMGA2) is an oncogenic architectural transcription factor aberrantly overexpressed in multiple cancers; yet its function and regulatory mechanisms in iCCA are poorly defined. This study aimed to elucidate the clinical significance and molecular mechanism of HMGA2 in iCCA progression.

**Methods:**

We integrated analyses across 4 independent iCCA cohorts (The Cancer Genome Atlas, 2 Zhongshan Hospital cohorts, and our 192-patient institutional cohort). Functional investigations were conducted using iCCA cell lines and multiple mouse models, including xenograft, syngeneic, YAP/AKT-driven spontaneous iCCA, and metastasis models.

**Results:**

We demonstrated that HMGA2 was significantly upregulated in iCCA, correlating with poor survival, and exhibited sexually dimorphic prognostic effects with a female-specific link to perineural invasion. Functionally, HMGA2 depletion suppressed iCCA cell proliferation, migration, in vivo tumor growth and metastasis. Mechanistically, HMGA2 expression was positively regulated by the N^6^-methyladenosine reader insulin-like growth factor 2 messenger RNA-binding protein 1 (IGF2BP1), which directly bound to and stabilized HMGA2 messenger RNA via its KH3-4 domains in an N^6^-methyladenosine–dependent manner. High IGF2BP1 expression predicted poor iCCA prognosis, was required for HMGA2–driven progression, and the axis promoted PI3K-AKT pathway activation.

**Conclusions:**

Our results reveal a critical role for the IGF2BP1–HMGA2 axis in iCCA pathogenesis, thereby highlighting its potential as a therapeutic target.

**Summary**

We identify high-mobility group protein A2 (HMGA2) as a key oncogenic driver of intrahepatic cholangiocarcinoma growth and metastasis. Insulin-like growth factor 2 messenger RNA-binding protein 1 (IGF2BP1) positively regulates HMGA2 expression via m^6^A modification, with the IGF2BP1–HMGA2 axis promoting PI3K–AKT pathway activation in intrahepatic cholangiocarcinoma, highlighting a promising therapeutic strategy.

**What You Need to Know**

**Background**

Intrahepatic cholangiocarcinoma (iCCA) is a lethal cancer with limited therapies. High-mobility group protein A2 (HMGA2) overexpression is linked to poor prognosis, but its role and regulation in iCCA progression remain unclear.

**Impact**

We identify HMGA2 as a key driver of iCCA progression. The N^6^-methyladenosine reader insulin-like growth factor 2 messenger RNA-binding protein 1 (IGF2BP1) stabilizes HMGA2 messenger RNA, defining the IGF2BP1–HMGA2 axis as a novel prognostic biomarker and promising therapeutic target.

**Future Directions**

Future studies will prioritize developing novel inhibitors targeting the IGF2BP1–HMGA2 axis, systematically evaluate their synergistic potential with standard therapies, and validate clinical efficacy in large-scale cohorts of patients with iCCA.

The global incidence of intrahepatic cholangiocarcinoma (iCCA) is steadily increasing, yet it remains a highly aggressive malignancy with a dismal prognosis.^1^^,^^2^ Although surgical resection represents the primary curative treatment, only a minority of patients are eligible for this intervention. Even among these eligible patients, the 5-year overall survival (OS) rate postresection remains modest, at merely 25%–40%.^2^ For advanced unresectable iCCA, first-line standard systemic therapies are based on gemcitabine plus cisplatin, with the anti- PD-L1 inhibitor durvalumab plus this chemotherapy doublet as the latest preferred standard of care.^3^ Additional standard iCCA regimens include second-line folinic acid, fluorouracil, and oxaliplatin (FOLFOX), and adjuvant capecitabine for patients following curative resection.^1^^,^^4^ Despite the identification of multiple targetable genetic alterations in iCCA and continuous advances in clinical research, current therapeutic strategies are still hampered by limited efficacy and acquired resistance.^5^, ^6^, ^7^

High-mobility group protein A2 (HMGA2), a nonhistone chromatin architectural transcription factor, functions as a master regulator of gene expression by modulating chromatin conformation and DNA accessibility.^8^ HMGA2 is aberrantly overexpressed across multiple malignancies and drives oncogenic phenotypes, including uncontrolled proliferation, invasion, metastasis, and therapy resistance.^9^ Mechanistically, HMGA2 activates critical signaling pathways such as Wnt/β-catenin, mTOR, TGF-β, and Akt, positioning it as a central orchestrator of tumorigenesis and cancer progression.^9^, ^10^, ^11^, ^12^ Although its oncogenic role is well-established in pancreatic,^13^ colorectal,^14^ and esophageal cancers,^12^ emerging clinical evidence links HMGA2 dysregulation to cholangiocarcinoma. It acts as an independent adverse prognostic factor in both perihilar/distal extrahepatic cholangiocarcinoma (correlating with aggressive pathologic features)^15^^,^^16^ and iCCA (associated with lymph node metastasis and shortened patient survival).^17^ The molecular functions and clinical relevance of HMGA2 in iCCA remain incompletely characterized and warrant further investigation.

N^6^-methyladenosine (m^6^A), the most prevalent internal modification in eukaryotic messenger RNA (mRNA), dynamically regulates RNA metabolism—including stability, splicing, nuclear export, and translation—through reversible chemical alterations.^18^^,^^19^ This modification is precisely controlled by 3 classes of regulatory factors: writers (methyltransferases), erasers (demethylases), and readers (m^6^A-binding proteins), which cooperatively fine-tune m^6^A deposition and function.^19^ As a key reader protein, insulin-like growth factor 2 mRNA-binding protein 1 (IGF2BP1) recognizes m^6^A-modified transcripts to enhance their stability and translation. In cancer contexts, IGF2BP1 overexpression promotes tumorigenesis by stabilizing oncogenic mRNAs (eg, MYCN, HEG1, eIF4G) that frequently undergo m^6^A modification.^20^, ^21^, ^22^ Notably, IGF2BP1 drives malignant progression in iCCA through m^6^A-dependent regulation of c-Myc and ZIC2, highlighting its therapeutic relevance.^23^ However, whether IGF2BP1 stabilizes additional mRNA targets to promote iCCA progression, as well as the underlying molecular mechanisms, remains incompletely characterized.

Based on integrated analyses of clinical datasets and functional validation, this study demonstrates that HMGA2 is significantly upregulated in iCCA and strongly correlates with adverse clinical outcomes, including shortened survival and advanced disease progression. Inhibition of HMGA2 not only significantly suppressed iCCA progression in vitro and in vivo, but also inhibited hepatic and pulmonary metastases of iCCA, underscoring its critical role in driving malignant progression. Furthermore, IGF2BP1—coexpressed with HMGA2 and associated with poor prognosis—enhances HMGA2 mRNA stability through m^6^A-dependent mechanisms, with the KH3-4 domains of IGF2BP1 being essential for this regulation. The IGF2BP1–HMGA2 axis promotes activation of the phosphoinositide 3-kinase (PI3K)-AKT signaling pathway during iCCA progression. Collectively, these results establish the IGF2BP1–HMGA2 axis as a key driver of iCCA malignant progression and identify it as a promising therapeutic target for this aggressive malignancy.

## Results

### HMGA2 Is Elevated in iCCA and Associated With Poor Prognosis of Patients With iCCA

**Figure 1:**
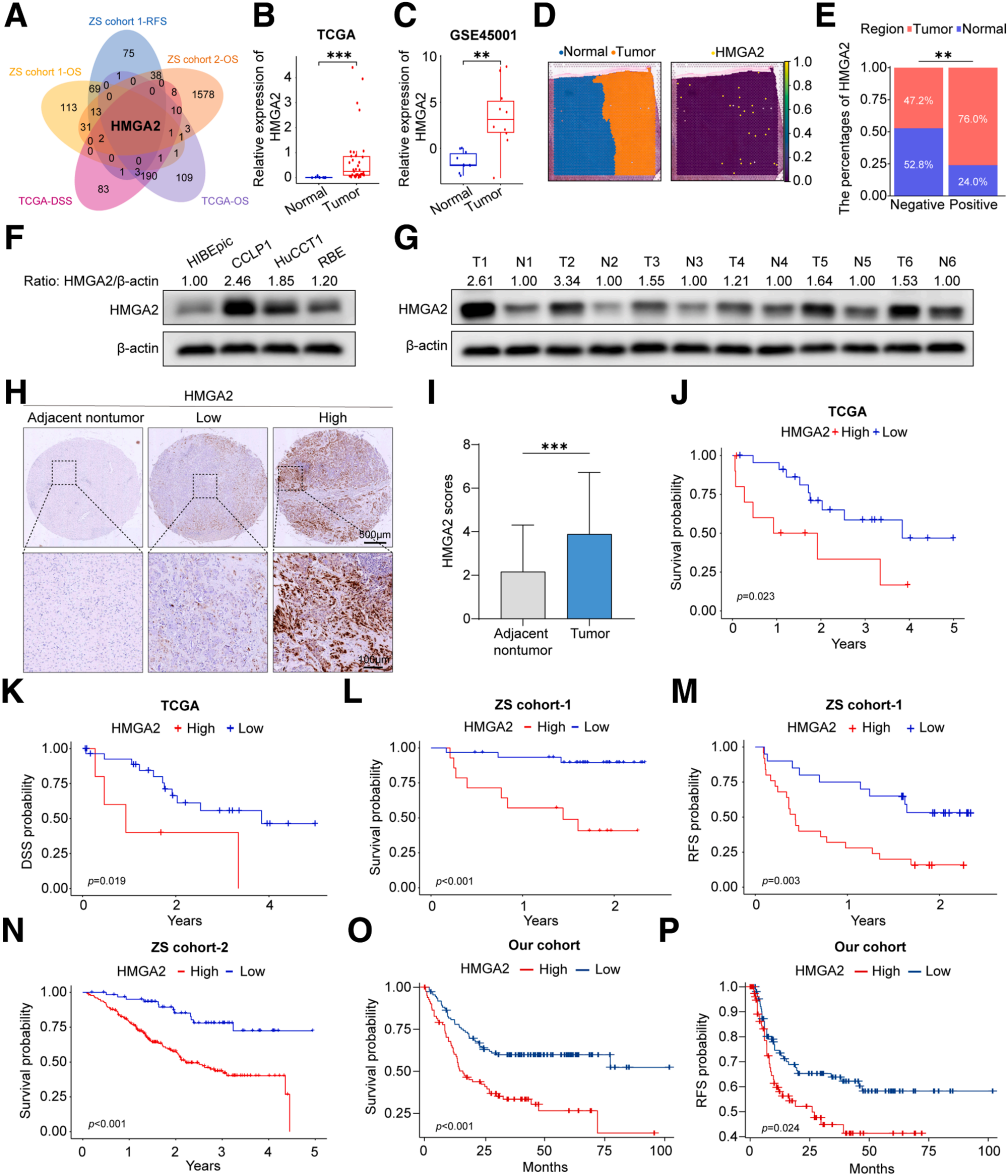
**The expression levels of HMGA2 in iCCA.** (*A*) The Venn diagram depicting the overlap of significant risk genes identified by univariate Cox regression analyses, using OS and DSS from TCGA-CHOL database, OS and RFS from ZS cohort-1, and OS from ZS cohort-2 as endpoints. (*B* and *C*) Comparison of HMGA2 expression levels between tumor samples and adjacent normal tissues in TCGA-CHOL database (*B*) and GSE45001 datasets (*C*). (*D*) Spatial annotation of the iCCA tissue slice (*left*) and spatial expression of HMGA2 (*right*) (Visium platform). *Yellow color* indicates higher normalized transcript abundance. (*E*) Barplot shows the percentage of the HMGA2 expression levels among tumor and normal regions of the iCCA tissue slice. (*F*) Detection of HMGA2 expression levels in iCCA cells by Western blot. (*G*) Detection of HMGA2 expression levels in iCCA tissues and matched adjacent normal tissues by Western blot. (*H*) Representative pictures of IHC staining of HMGA2 in iCCA tissues and matched adjacent nontumor tissues (N = 192). Scale bars, 500 μm (upper) and 100 μm (lower). (*I*) Detection of HMGA2 expression levels by IHC in our cohort. (*J–P*) Kaplan–Meier curves showing the association of high/low HMGA2 expression with OS, DSS and RFS in the TCGA-CHOL database (*J* and *K*), ZS cohort-1 (*L* and *M*), ZS cohort-2 (*N*), and our cohort (*O* and *P*). The Student *t* test was used to analyze the data; ∗∗*P* < .01; ∗∗∗*P* < .001.

To elucidate the regulatory mechanisms underlying key phenotypes of iCCA, we performed a comprehensive integrated analysis of 3 independent public datasets: The Cancer Genome Atlas (TCGA), Zhongshan Hospital cohort 1 (ZS cohort-1), and Zhongshan Hospital cohort 2 (ZS cohort-2). Through systematic evaluation of 3 clinical endpoints—OS, recurrence-free survival (RFS), and disease-specific survival (DSS)—we identified molecular markers significantly associated with poor prognosis. Subsequent cross-validation across multiple datasets consistently identified HMGA2 as a key prognostic indicator of patients with iCCA (Figure 1*A*). Both the TCGA and GSE45001 datasets confirmed significant HMGA2 upregulation in iCCA compared with adjacent normal tissues (Figure 1*B* and *C*). The analysis of published spatial transcriptome data provided further confirmation that HMGA2 expression localizes in a manner consistent with our findings^24^ (Figure 1*D* and *E*). Western blotting confirmed the upregulation of HMGA2 in multiple cholangiocarcinoma cell lines (CCLP1, HuCCT1, and RBE) compared with normal biliary epithelial cells (HIBEpic) (Figure 1*F*). In addition, HMGA2 expression was significantly upregulated in iCCA tissues relative to matched adjacent normal tissues (Figure 1*G*). Further validation by immunohistochemistry (IHC) on the tissue microarrays (TMAs) from our cohort of 192 patients with iCCA revealed that HMGA2 expression levels were markedly elevated in tumor tissues compared with adjacent nontumor tissues (Figure 1*H* and *I*).

**Figure 2:**
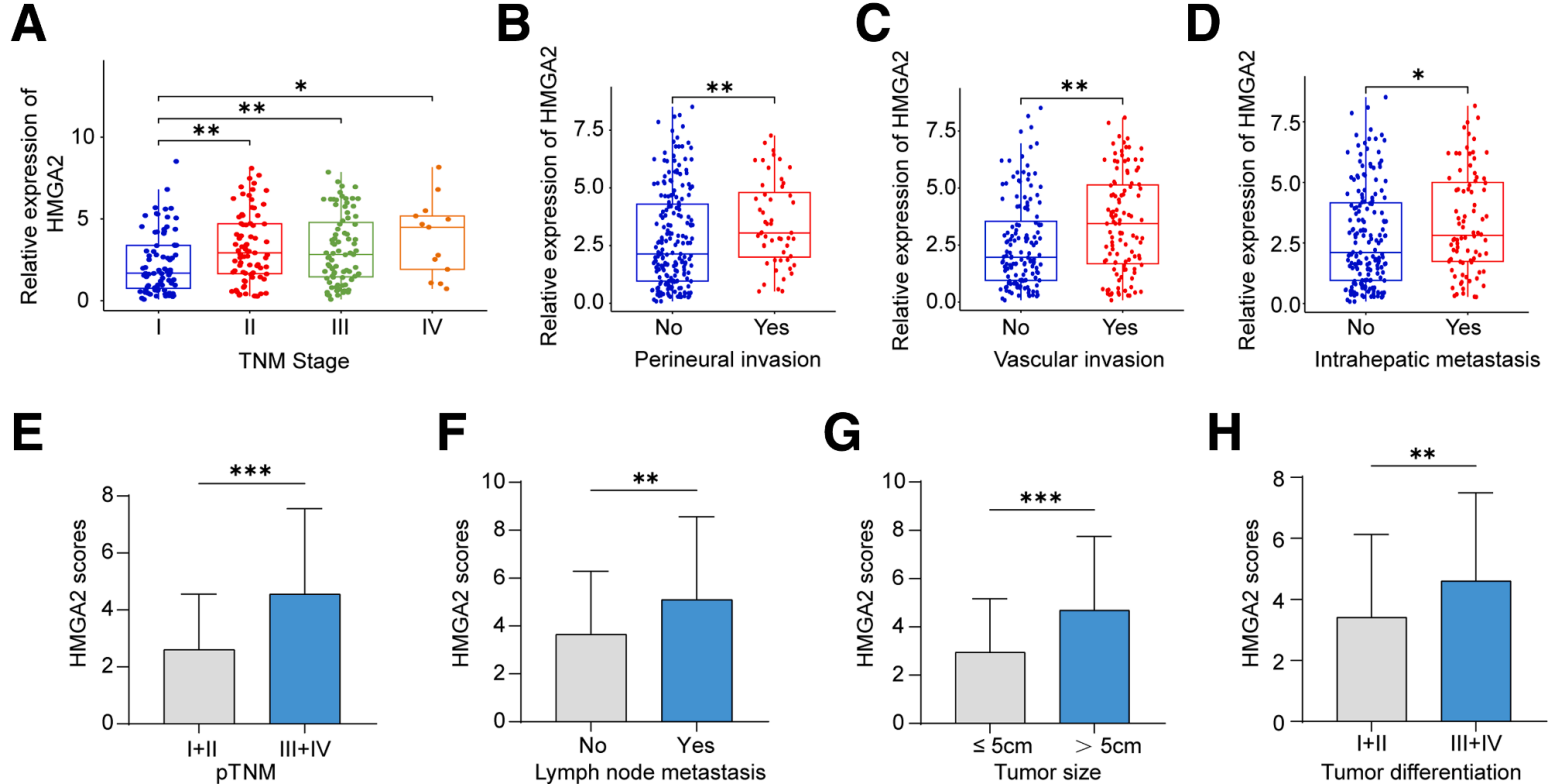
**Prognostic significance of HMGA2 expression in iCCA.** (*A–D*) The difference of HMGA2 expression among TNM stage groups (*A*), PNI (*B*), vascular invasion (*C*), and intrahepatic metastasis (*D*) in the ZS cohort-2. (*E–H*) The difference of HMGA2 expression among pathologic TNM (pTNM) stage groups (*E*), lymph node metastasis (*F*), tumor size (*G*), and tumor differentiation (*H*) statuses in our cohort. The Student *t* test or one-way/two-way analysis of variance was used to analyze the data; ∗*P* < .05; ∗∗*P* < .01; ∗∗∗*P* < .001.

To further evaluate the clinical significance of HMGA2 in iCCA, we conducted survival analysis on the TCGA datasets, which revealed that elevated HMGA2 expression was significantly associated with reduced OS and DSS in iCCA (Figure 1*J* and *K*). Furthermore, assessment of ZS cohort-1 revealed that high HMGA2 expression correlated with poorer OS and RFS (Figure 1*L* and *M*). This association was subsequently validated in ZS cohort-2, where increased HMGA2 expression independently predicted shortened OS in patients with iCCA (Figure 1*N*). Similarly, the patients in the high HMGA2 group had significantly shorter OS and RFS than those in the low HMGA2 group in our validation cohort (Figure 1*O* and *P*). Correlation analyses in ZS cohort-2 revealed that HMGA2 expression was significantly elevated in patients with advanced TNM stages (II–IV) compared with those in stage I (Figure 2*A*). In addition, high HMGA2 levels were correlated with the presence of perineural invasion (PNI), vascular invasion, and intrahepatic metastasis (Figure 2*B–D*). Similarly, analysis within our cohort demonstrated that HMGA2 expression was significantly elevated in patients with advanced pathologic TNM stages III/IV compared with those with stages I/II (Figure 2*E*) and was markedly upregulated in patients with lymph node metastasis (Figure 2*F*). Moreover, higher HMGA2 levels were associated with larger tumor size (>5 cm), and poor tumor differentiation (III/IV) (Figure 2*G* and *H*, Table 1). Overall, these data demonstrate that HMGA2 plays a crucial role in promoting iCCA progression.

**Table 1:** Comparison of Clinicopathologic Profiles Between Low and High HMGA2 Expression in Our Cohort (N = 192)

Variable	HMGA2 expression		*P*
	Low (n = 110)	High (n = 82)	
Gender			
Female	44	34	.838
Male	66	48	
Age, *y*			
≥50	92	63	.237
<50	18	19	
HBsAg			
Negative	71	58	.366
Positive	39	24	
HBcAb			
Positive	73	57	.644
Negative	37	25	
ALT			
≤75	82	62	.866
>75	28	20	
Tbil			
≤17.1	81	54	.243
>17.1	29	28	
GGT			
≤60	60	34	.073
>60	50	48	
Tumor size, cm			
≤5	65	23	**<.001**
>5	45	59	
Tumor number			
Single	102	76	.991
Multiple	8	6	
Tumor differentiation			
I + II	76	40	**.004**
III + IV	34	42	
pTNM			
I + II	47	19	**.005**
III + IV	63	63	
Regional lymph node metastasis			
Negative	96	64	.090
Positive	14	18	

NOTE. Boldface *P* values indicate statistical significance. All categorical comparisons were performed using the χ² test.

ALT, alanine aminotransferase; GGT, gamma-glutamyl transferase; HbcAb, hepatitis B core antibody; HBsAg, hepatitis B surface antigen; HMGA2, high-mobility group protein A2; pTNM, pathologic TNM; Tbil, total bilirubin.

### HMGA2 Exhibits Sexually Dimorphic Prognostic Effects in iCCA, With a Female-Specific Link to Perineural Invasion

**Figure 3:**
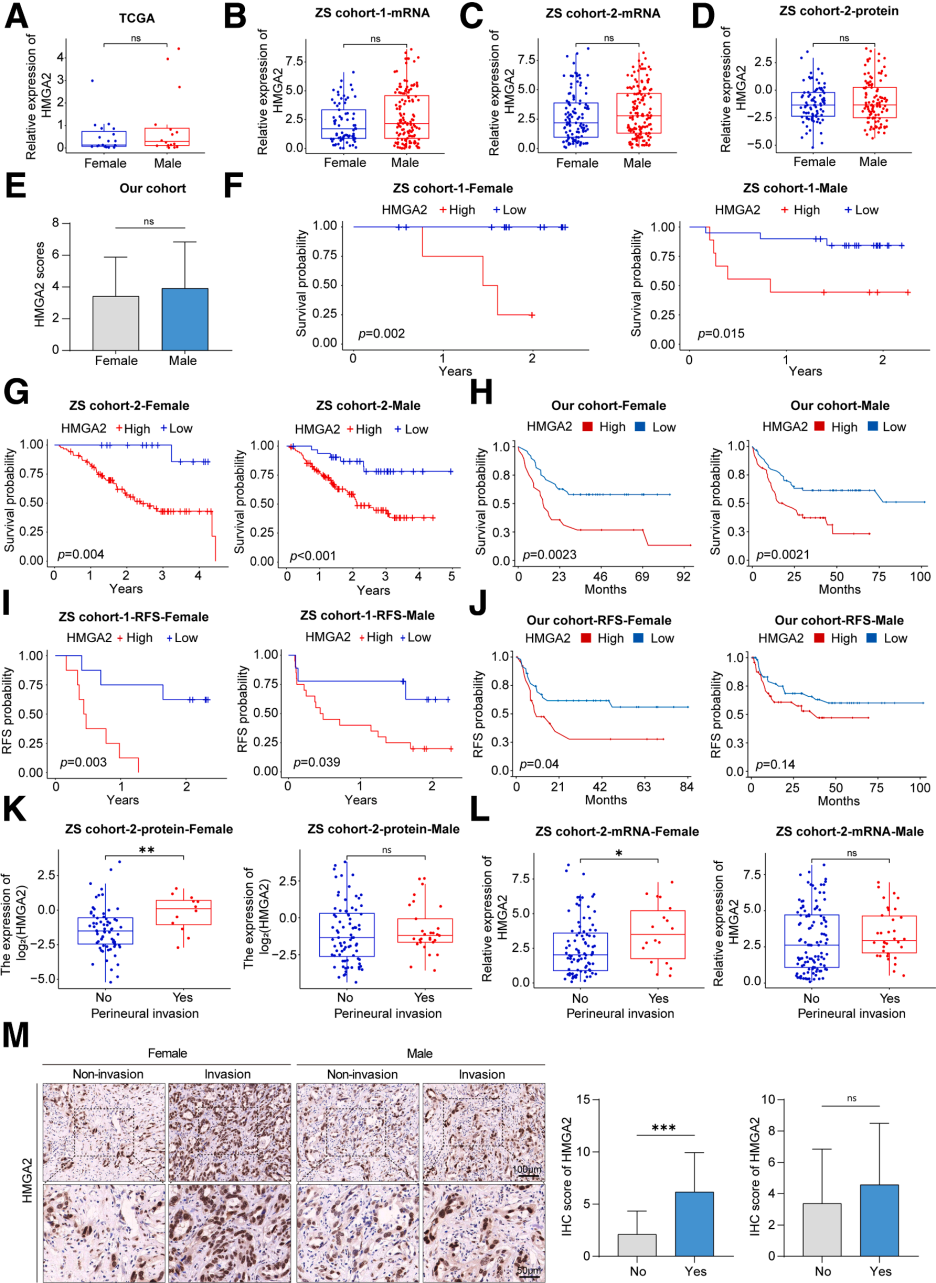
**Sex-specific prognostic role of HMGA2 and its female-specific correlation with PNI in iCCA.** (*A–E*) HMGA2 expression levels in female and male patients with iCCA across 4 independent cohorts: TCGA (*A*), ZS cohort-1 (mRNA, *B*), ZS cohort-2 (mRNA, *C*; protein, *D*), and our cohort (*E*). (*F–J*) Kaplan–Meier analysis of OS (*F–H*) and RFS (*I* and *J*) stratified by HMGA2 expression in female and male patients from the ZS cohort-1, the ZS cohort-2, and our cohort. (*K* and *L*) HMGA2 protein (*K*) and mRNA (*L*) expression in female and male ZS cohort-2 patients with or without PNI. (*M*) Representative IHC staining and quantification of HMGA2 expression in iCCA tissues with or without PNI, stratified by sex. Scale bars, 100 μm (*upper*) and 50 μm (*lower*). The Student *t* test was used to analyze the data; ∗*P* < .05; ∗∗*P* < .01; ∗∗∗*P* < .001; ns, not significant.

To further delineate whether the prognostic impact of HMGA2 in iCCA exhibits sexual dimorphism, we analyzed its expression, prognostic value, and clinicopathologic correlations across 4 independent iCCA cohorts: the TCGA dataset, the ZS cohort-1, the ZS cohort-2, and our institutional cohort. The results showed that baseline mRNA and protein expression levels of HMGA2 in tumor tissues were comparable between female and male patients with iCCA across all included cohorts (Figure 3*A–E*). These findings indicated no significant sex-specific differences in the basal expression of HMGA2. In OS analyses, high HMGA2 expression uniformly predicted unfavorable clinical outcomes in both female and male patients with iCCA across the ZS cohort-1, the ZS cohort-2, and our institutional cohort (Figure 3*F–H*). These findings consistently established HMGA2 as a robust, sex-independent adverse prognostic biomarker for OS.

In contrast, the prognostic performance of HMGA2 for RFS exhibited pronounced sexual dimorphism: although high HMGA2 expression was significantly associated with shortened RFS in both sexes in ZS cohort-1, this correlation reached higher statistical significance in female patients (*P* = .003 vs *P* = .039 in males) (Figure 3*I*). Consistently in our independent cohort, this prognostic association reached statistical significance exclusively in female patients, with no statistically significant correlation detected in the male subgroup (Figure 3*J*).

We further performed sex-stratified analyses of the correlations between HMGA2 expression and various clinicopathologic features of iCCA. Notably, only PNI showed a sex-dependent association with HMGA2 expression. In the ZS cohort-2, female patients with iCCA and PNI displayed markedly higher HMGA2 mRNA and protein levels compared with those without PNI (Figure 3*K* and *L*). Conversely, no significant difference in HMGA2 expression was observed between male patients with and without PNI. This female-specific association was further validated in our patients with iCCA: HMGA2 protein levels were markedly elevated in female patients with iCCA and PNI but not in males (Figure 3*M*).

Collectively, this study established HMGA2 as a consistent sex-independent adverse prognostic biomarker for OS in patients with iCCA. The stronger prognostic impact of HMGA2 on RFS in female patients may be attributed to its female-specific function in driving tumor recurrence via promoting PNI. These findings provide novel clinical evidence for deciphering the molecular mechanisms underlying sex-disparate recurrence and progression of iCCA.

### Knockdown of HMGA2 Effectively Suppresses the Progression of iCCA In Vitro

**Figure 4:**
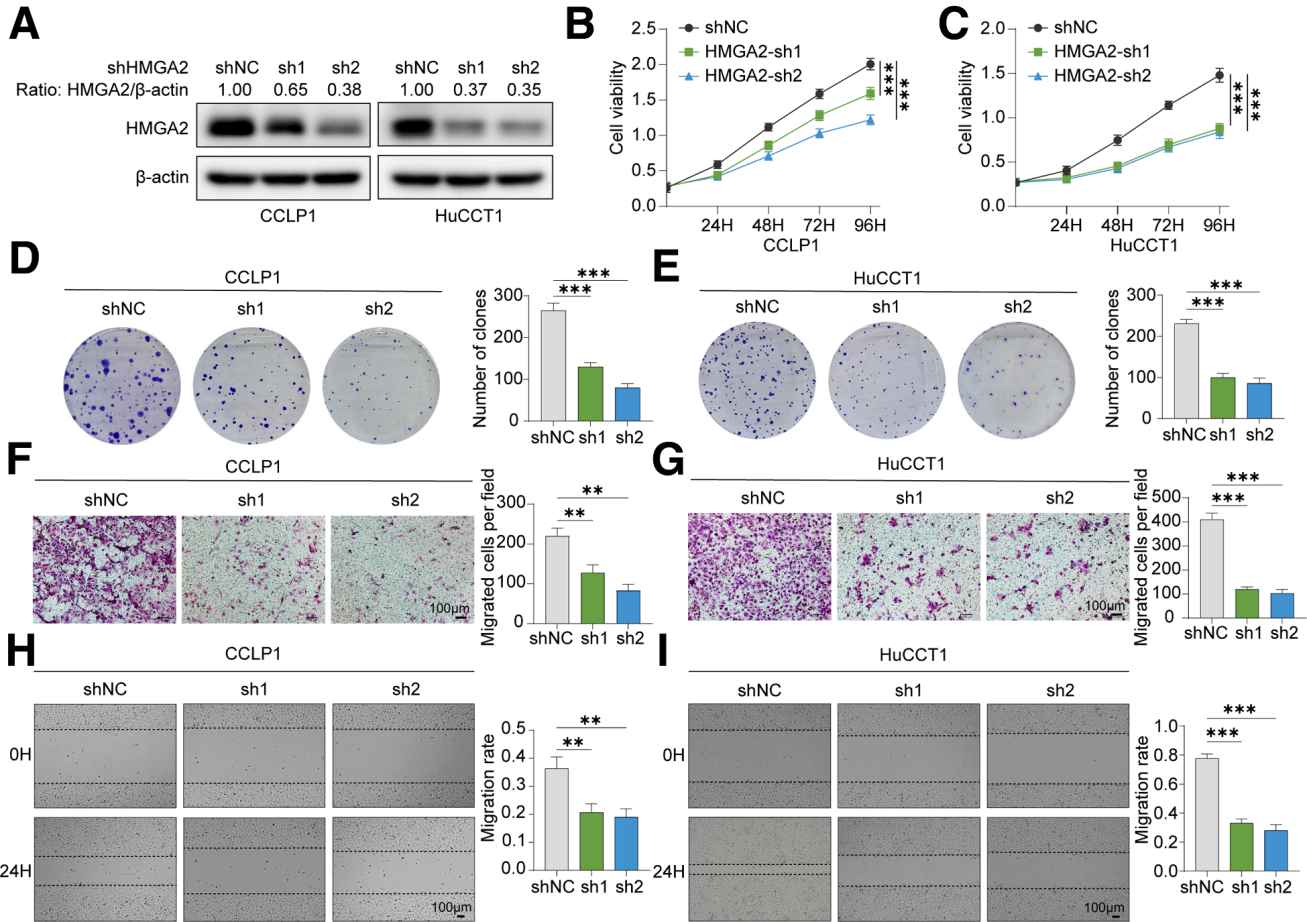
**The effects of HMGA2 on in vitro proliferation, clonogenicity, and migration.** (*A*) Western blot validation of HMGA2 knockdown efficiency in CCLP1 and HuCCT1 cells. (*B* and *C*) Proliferation curves of CCLP1 (*B*) and HuCCT1 (*C*) cells after HMGA2 shRNA transfection. (*D* and *E*) Colony formation assays in HMGA2-knockdown CCLP1 (*D*) and HuCCT1 (*E*) cells. (*F* and *G*) Transwell migration assays in HMGA2-silenced CCLP1 (*F*) and HuCCT1 (*G*) cells. Scale bars, 100 μm. (*H* and *I*) Wound-healing assays in HMGA2-knockdown CCLP1 (*H*) and HuCCT1 (*I*) cells. Scale bars, 100 μm. All results are shown as mean ± standard deviation (n = 3). One-way/2-way analysis of variance was used to analyze the data; ∗∗*P* < .01; ∗∗∗*P* < .001.

Given that HuCCT1 and CCLP1 exhibited the highest HMGA2 protein expression among the 3 iCCA cell lines examined (Figure 1*F*), we selected them for subsequent HMGA2 functional studies. Two independent short hairpin RNAs (shRNAs) were used to achieve specific HMGA2 knockdown, and their knockdown efficiencies were confirmed by Western blot (Figure 4*A*). Functional assays demonstrated that HMGA2 depletion significantly impaired cell proliferation and colony formation in both iCCA cell lines (Figure 4*B–E*). Moreover, Transwell and wound healing assays revealed that HMGA2 knockdown substantially reduced the migratory capacity of iCCA cells (Figure 4*F–I*).

**Figure 5:**
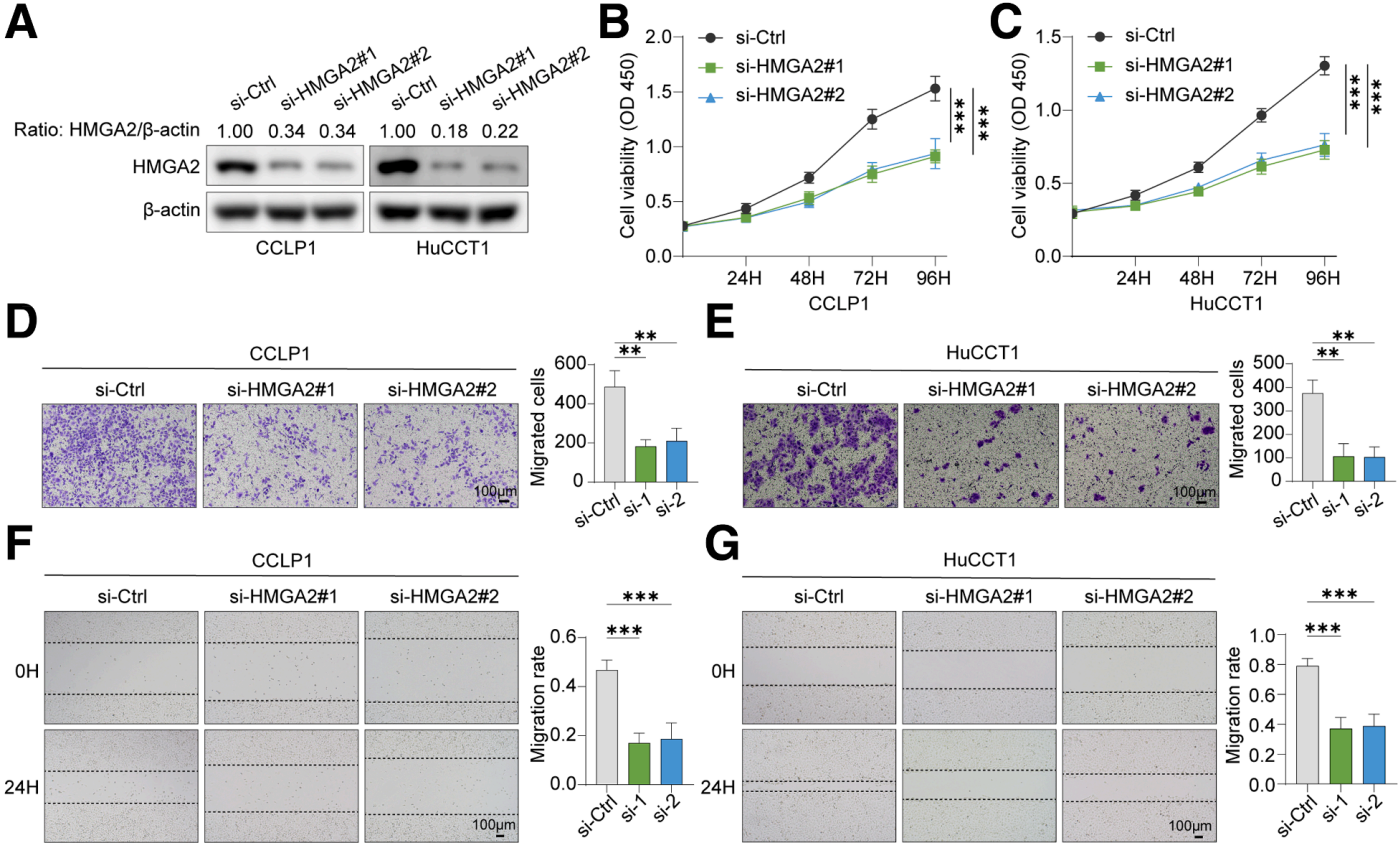
**The effects of transient HMGA2 knockdown on in vitro proliferation and migration.** (*A*) Western blot validation of HMGA2 knockdown efficiency in CCLP1 and HuCCT1 cells using 2 independent siRNAs. (*B* and *C*) Cell growth curve of CCLP1 and HuCCT1 cells transfected with 2 independent HMGA2 siRNAs or control. (*D* and *E*) Transwell migration assays after HMGA2 knockdown in CCLP1 and HuCCT1 cells. Scale bars, 100 μm. (*F* and *G*) Wound-healing assays after HMGA2 silencing in CCLP1 and HuCCT1 cells. Scale bars, 100 μm. All results are shown as mean ± standard deviation (n = 3). One-way/2-way analysis of variance was used to analyze the data; ∗∗*P* < .01; ∗∗∗*P* < .001.

To strengthen the robustness of these findings, we performed independent validation using 2 distinct small interfering RNAs (siRNAs) targeting HMGA2 in CCLP1 and HuCCT1 cells. Consistent with the shRNA knockdown results, siRNA-mediated HMGA2 downregulation significantly inhibited iCCA cell proliferation and migration in vitro (Figure 5*A–G*).

Collectively, these in vitro results from 2 independent knockdown systems demonstrate that HMGA2 is critical for iCCA cell proliferation, clonogenicity, and migration, confirming its oncogenic role in iCCA.

### Knockdown of HMGA2 Potently Inhibits In Vivo Progression of iCCA

**Figure 6:**
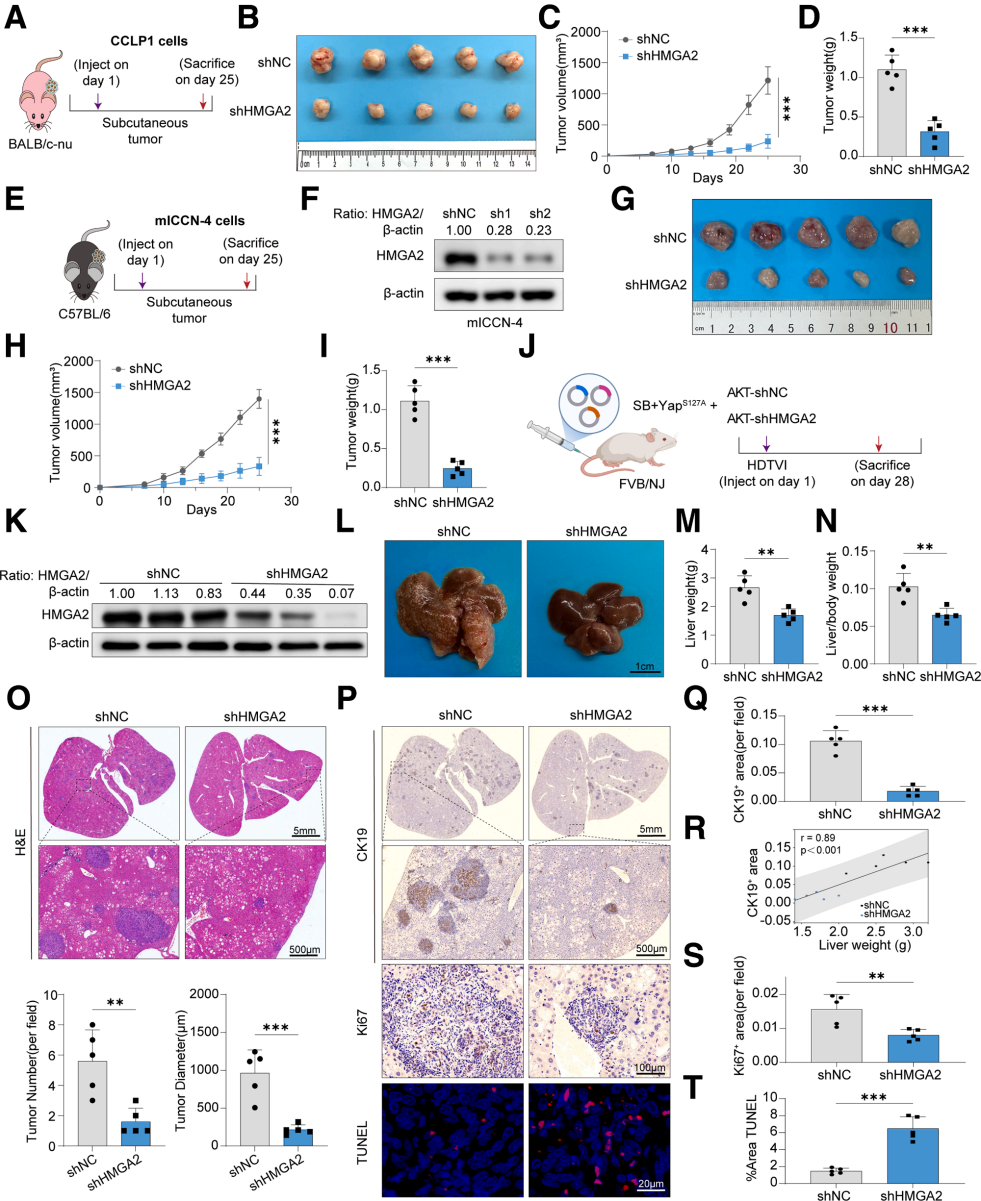
**The effects of HMGA2-depleted on in vivo tumor progression of iCCA in subcutaneous tumor and hydrodynamic models.** (*A*) Schematic illustrating the establishment of the subcutaneous tumor model using BALB/c nude mice. (*B*) Overview of tumors in BALB/c-nu mice subcutaneously implanted with HMGA2-depleted and control CCLP1 cells (n = 5). (*C* and *D*) Tumor growth curve (*C*) and tumor weights (*D*) of the BALB/c-nu mice implanted with HMGA2-depleted and control CCLP1 cells. (*E*) Schematic illustrating the establishment of the subcutaneous tumor model using C57BL/6 mice. (*F*) Western blot analysis of HMGA2 protein expression after knockdown in mICCN-4 cells. (*G*) Overview of tumors in C57BL/6 mice subcutaneously implanted with HMGA2-depleted and control mICCN-4 cells (n = 5). (*H* and *I*) Tumor growth curve (*H*) and tumor weights (*I*) of the C57BL/6 mice implanted with HMGA2-depleted and control mICCN-4 cells. (*J*) Diagram of the study design in the HDTVI mouse model (n = 5). (*K*) HMGA2 protein levels in mouse tumor tissues from the shNC and shHMGA2 groups were analyzed by Western blotting. (*L*) Representative images of shNC and shHMGA2 mouse livers. (*M* and *N*) Liver weight (*M*) and ratios of liver weight to body weight (*N*) in shNC and shHMGA2 mice. (*O*) Representative H&E staining images of livers in shNC and shHMGA2 mice and quantitative analysis of tumor number and diameter based on H&E staining. (*P*) Representative IHC staining of CK19, Ki67, and fluorescence staining of TUNEL in shNC and shHMGA2 mouse livers. (*Q*) Quantitative analysis of CK19-positive (CK19^+^) area in shNC and shHMGA2 mouse livers. (*R*) Correlation between liver weight and CK19^+^ proportion; *P* values were calculated by Pearson correlation. (*S* and *T*) Quantitative analysis of Ki67-positive (Ki67^+^) area (*S*), and TUNEL-positive (TUNEL^+^) area (*T*) in shNC and shHMGA2 mouse livers. All results are shown as mean ± standard deviation (n = 5). The Student *t* test was used to analyze the data; ∗∗*P* < .01; ∗∗∗*P* < .001.

To investigate the critical role of HMGA2 in vivo, we first utilized 2 independent mouse models. In a xenograft mouse model (Figure 6*A*), HMGA2 knockdown (shHMGA2) significantly attenuated tumor growth compared with shNC controls, as evidenced by reduced tumor size and weight at endpoint (Figure 6*B–D*). Similarly, in a syngeneic subcutaneous tumor model generated by implanting murine iCCA cells (mICCN-4) into immunocompetent mice (Figure 6*E*), HMGA2 depletion also suppressed tumor progression (Figure 6*F–I*).

We performed further validation in a spontaneous iCCA model established in FVB/NJ mice via hydrodynamic tail vein injection (HDTVI) (Figure 6*J*).^25^, ^26^, ^27^ This model used the Sleeping Beauty (SB) transposon system, Yap^S127A^, and AKT plasmids harboring either HMGA2-targeting shRNA (AKT-shHMGA2) or control shRNA (AKT-shNC). HMGA2 knockdown efficiency in mouse tissues was confirmed by WB (Figure 6*K*). At 4 weeks post-injection, mice in the shNC group developed substantial tumor burden, whereas mice in the shHMGA2 group showed only minor hepatic lesions (Figure 6*L*). Mice in the shHMGA2 group displayed significantly reduced tumor burden compared with shNC controls, reflected by decreased liver weight and liver-to-body weight ratio (Figure 6*M* and *N*). Hematoxylin and eosin (H&E) staining revealed fewer and smaller tumor lesions in shHMGA2 mice (Figure 6*O*). Histological analysis demonstrated markedly reduced cytokeratin 19 (CK19)-positive area in shHMGA2 group mice (Figure 6*P* and *Q*), with liver weight strongly correlated with CK19-positive area proportion (Figure 6*R*). IHC staining of the proliferation marker protein Ki67 and terminal deoxynucleotidyl transferase-mediated dUTP nick end labeling (TUNEL) immunofluorescence assays revealed that HMGA2 knockdown significantly reduced proliferation and markedly enhanced apoptosis of tumor cells in mouse models in vivo (Figure 6*P*, *S*, and *T*).

These in vivo data establish that HMGA2 acts as a key oncogenic driver of iCCA, promoting tumor progression in vivo by enhancing tumor cell proliferation and restraining cell apoptosis.

### Overexpression of HMGA2 Potently Promotes In Vivo Progression of iCCA

**Figure 7:**
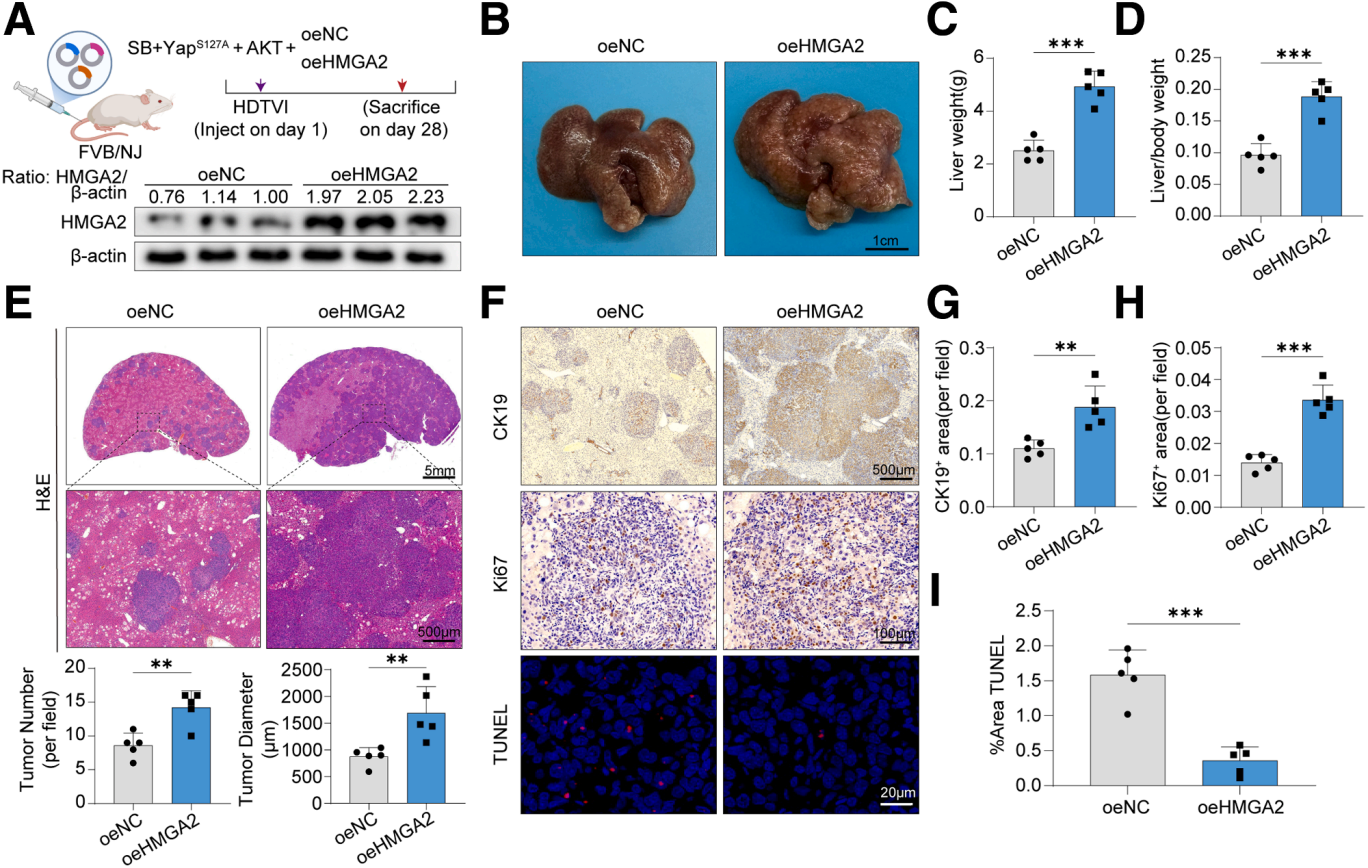
**The effects of HMGA2 overexpression on in vivo tumor progression of iCCA in hydrodynamic models.** (*A*) *Upper*: Schematic illustrating the establishment of the HDTVI spontaneous tumor model (n = 5). *Lower*: HMGA2 protein levels in mouse tumor tissues from the oeNC and oeHMGA2 groups were analyzed by Western blotting. (*B–D*) Representative gross pathologic photographs (*B*) and statistical analysis of liver weights (*C*) and ratio of liver weight to body weight (*D*) in oeNC and oeHMGA2 mice livers (n = 5). (*E*) Representative H&E-staining images of livers in oeNC and oeHMGA2 mice and quantitative analysis of tumor number and diameter based on H&E staining. (*F*) Representative IHC staining of CK19, Ki67, and fluorescence staining of TUNEL in oeNC and oeHMGA2 mouse livers. (*G–I*) Quantitative analysis of CK19^+^ area (*G*), Ki67^+^ area (*H*), and TUNEL^+^ area (*I*) in oeNC and oeHMGA2 mouse livers. All results are shown as mean ± standard deviation (n = 5). The Student *t* test was used to analyze the data; ∗∗*P* < .01; ∗∗∗*P* < .001.

To further validate the oncogenic function of HMGA2 in iCCA progression, we established a liver-specific HMGA2 overexpression iCCA mouse model via hydrodynamic transfection of the HMGA2 overexpression plasmid. The SB transposon system was coadministered with Yap^S127A^ and AKT constructs, together with either the HMGA2 overexpression plasmid (oeHMGA2) or the empty vector control (oeNC) (Figure 7*A*). Overexpression of HMGA2 in liver tissues was confirmed by Western blotting (Figure 7*A*).

At 4 weeks postinjection, mice in the oeHMGA2 group developed markedly increased tumor burden compared with oeNC controls, as evidenced by significantly elevated liver weight and liver-to-body weight ratios (Figure 7*B–D*). H&E staining further revealed that mice in the oeHMGA2 group developed more abundant and larger tumor nodules in the liver (Figure 7*E*). IHC analysis of CK19 showed that oeHMGA2 tumors exhibited significantly expanded CK19-positive areas (Figure 7*F* and *G*). IHC staining of Ki67 and TUNEL immunofluorescence assays confirmed that HMGA2 overexpression significantly enhanced tumor cell proliferation and markedly reduced apoptosis in vivo (Figure 7*F*, *H*, and *I*).

Collectively, these in vivo findings demonstrate that HMGA2 overexpression potently accelerates iCCA progression by promoting tumor cell proliferation and inhibiting cell apoptosis, further confirming HMGA2 as a critical oncogenic driver of iCCA.

### Knockdown of HMGA2 Inhibits In Vivo Metastasis of iCCA

As shown in Figure 2*A–F*, HMGA2 was significantly upregulated in advanced-stage iCCA and correlated with multiple metastatic features, including PNI, vascular invasion, intrahepatic metastasis, and lymph node metastasis. We further evaluated the in vivo metastatic potential of HMGA2 in iCCA.

**Figure 8:**
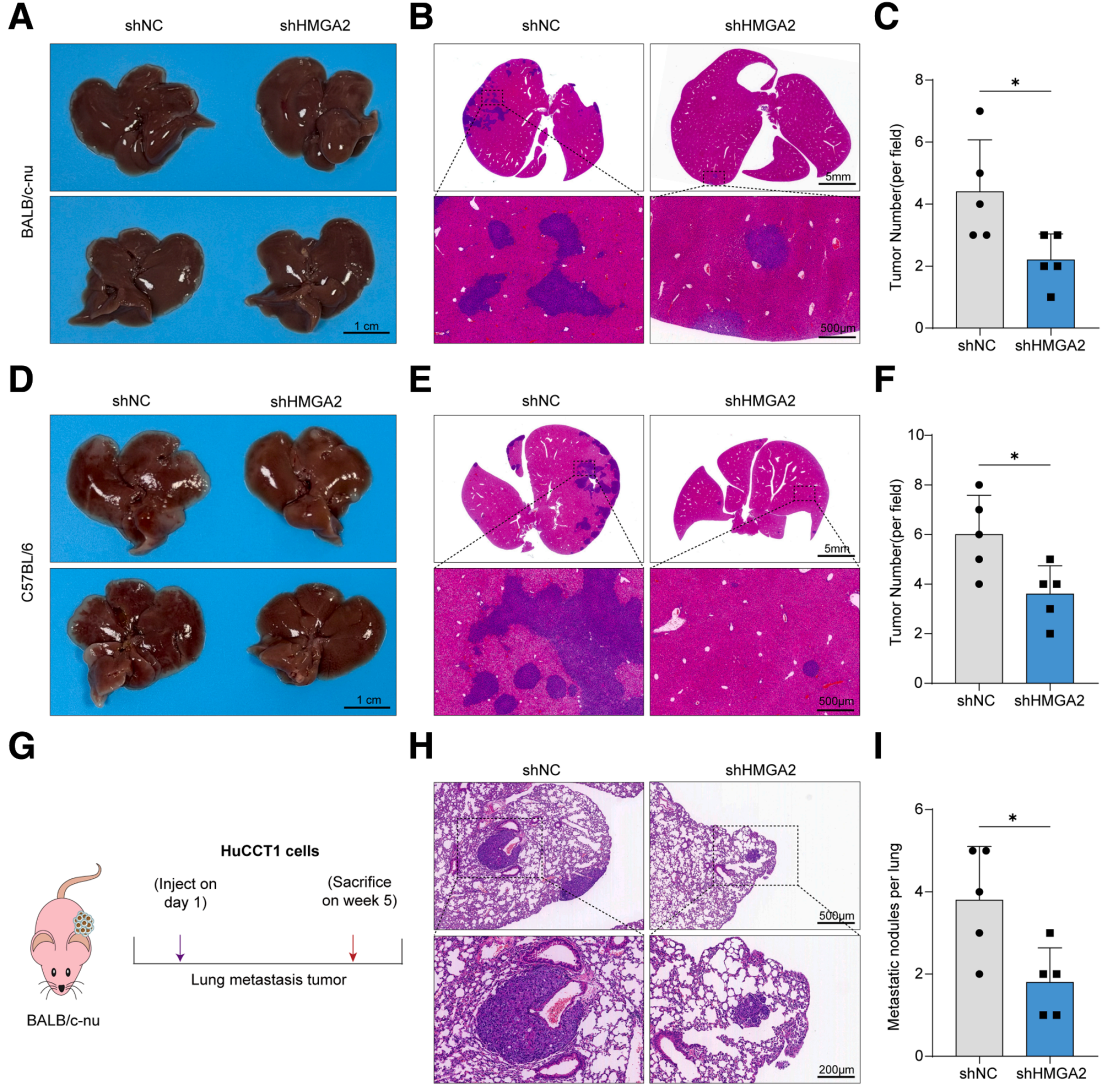
**HMGA2 knockdown inhibits iCCA liver and lung metastasis in vivo.** (*A*) Representative images of liver metastasis model in BALB/c-nu mice inoculated with HMGA2-depleted and control HuCCT1 cells (n = 5). (*B* and *C*) Representative H&E images (*B*) and quantification of the metastatic nodules (*C*) in the livers. (*D*) Representative images of liver metastasis model in C57BL/6 mice inoculated with HMGA2-depleted and control mICCN-4 cells (n = 5). (*E* and *F*) Representative H&E images (*E*) and quantification of the metastatic nodules (*F*) in the livers. (*G*) Schematic of the lung metastasis model in BALB/c-nu mice inoculated with HMGA2-depleted and control HuCCT1 cells (n = 5). (*H* and *I*) Representative H&E images (*H*) and quantification of metastatic lung nodules (*I*) in the lungs. All results are shown as mean ± standard deviation (n = 5). The Student *t* test was used to analyze the data; ∗*P* < .05.

To assess liver metastasis, control and HMGA2-depleted HuCCT1 cells were injected into nude mice via the spleen. Results showed significantly fewer liver metastatic nodules in the HMGA2-knockdown group than in the control group, as further confirmed by H&E staining (Figure 8*A–C*). Consistently, in immunocompetent C57BL/6 mouse models, HMGA2 knockdown markedly reduced the number of liver metastatic nodules and tumor burden compared with shNC controls (Figure 8*D–F*).

To evaluate lung metastasis, control and HMGA2-depleted HuCCT1 cells were injected into nude mice via the tail vein. Our results revealed markedly fewer lung metastatic nodules in the HMGA2-knockdown group compared with the control group, as verified by H&E staining (Figure 8*G–I*).

Collectively, these data demonstrate that HMGA2 knockdown potently suppresses iCCA hepatic and pulmonary metastasis in vivo, identifying HMGA2 as a critical driver of iCCA metastatic progression.

### HMGA2 Expression Correlates With the N^6^-Methyladenosine Reader IGF2BP1 in iCCA

**Figure 9:**
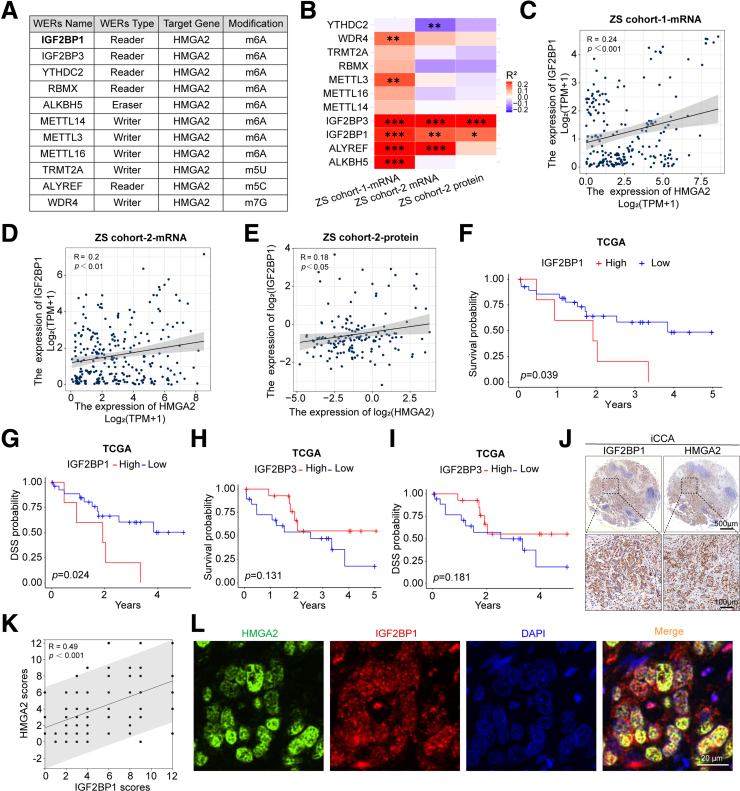
**HMGA2 and m^6^A reader IGF2BP1 are positively correlated in iCCA.** (*A*) Writers, erasers, and readers (WERs) of RNA modification that target HMGA2, as predicted by the RM2Target database. (*B*) Heatmap depicting the correlation analysis of the HMGA2 with potential RNA-modifying genes in the RM2Target database. *P* values were calculated by Pearson correlation; ∗*P* < .05; ∗∗*P* < .01; ∗∗∗*P* < .001. (*C–E*) Correlation analysis between IGF2BP1 and HMGA2 expression in independent iCCA patient cohorts: ZS cohort-1 (mRNA level, *C*), ZS cohort-2 (mRNA level, *D*; protein level, *E*). (*F* and *G*) Kaplan–Meier plot curves showing the association of IGF2BP1 high/low expression with OS (*F*) and DSS (*G*) in the TCGA-CHOL cohort. (*H* and *I*) Kaplan–Meier curves for OS (*H*) and DSS (*I*) stratified by IGF2BP3 expression (high vs low) in the TCGA-CHOL cohort. (*J*) Representative IHC staining of HMGA2 and IGF2BP1 in our iCCA patient cohort (n = 192). (*K*) Correlation analysis of HMGA2 and IGF2BP1 IHC scores in iCCA tissues. (*L*) Immunofluorescence staining showing colocalization of HMGA2 (*green*) and IGF2BP1 (*red*) in tumor tissues from patients with iCCA, with 4',6-diamidino-2-phenylindole (DAPI; *blue*) nuclear counterstaining. Scale bar, 20 μm.

Given the pivotal role of m^6^A modifications in cancer progression, we screened the RM2Target database to identify RNA modification genes potentially regulating HMGA2 expression.^28^, ^29^, ^30^ This analysis identified 11 candidate genes implicated in m^6^A-dependent regulation of HMGA2 (Figure 9*A*). We performed subsequent correlation analysis between their expression levels and HMGA2 across 2 independent cohorts (ZS cohort-1 and ZS cohort-2). This analysis revealed that only IGF2BP1 and IGF2BP3 exhibited statistically significant positive correlations with HMGA2 in all datasets (Figure 9*B–E*).

Further Kaplan–Meier analysis of TCGA data provided critical insights for candidate selection. The analysis demonstrated that elevated IGF2BP1 expression correlated with significantly reduced survival, whereas IGF2BP3 showed no statistically significant association with survival outcomes (Figure 9*F–I*). Based on this distinct prognostic value, we selected IGF2BP1 for further investigation, as its expression pattern demonstrated stronger clinical relevance in iCCA progression. Consistent with these findings, IHC analysis on the TMAs from our cohort confirmed a positive correlation between IGF2BP1 and HMGA2 protein levels (Figure 9*J* and *K*).

The integrated analysis of expression correlation and clinical outcome data thus positioned IGF2BP1 as a more promising candidate for understanding the HMGA2 regulatory network in iCCA pathogenesis. In addition, we performed double immunofluorescence staining of HMGA2 and IGF2BP1 in human iCCA tumor tissues. The results directly confirmed the coexpression of IGF2BP1 and HMGA2 proteins in these clinical specimens (Figure 9*L*).

Collectively, these results indicate a strong association between IGF2BP1 expression and HMGA2 upregulation in iCCA, consistent with a potential role for m^6^A-dependent mRNA stabilization. However, direct mechanistic evidence requires further experimental validation.

### HMGA2 Serves as a Key N^6^-Methyladenosine Modification Target of IGF2BP1 in iCCA

**Figure 10:**
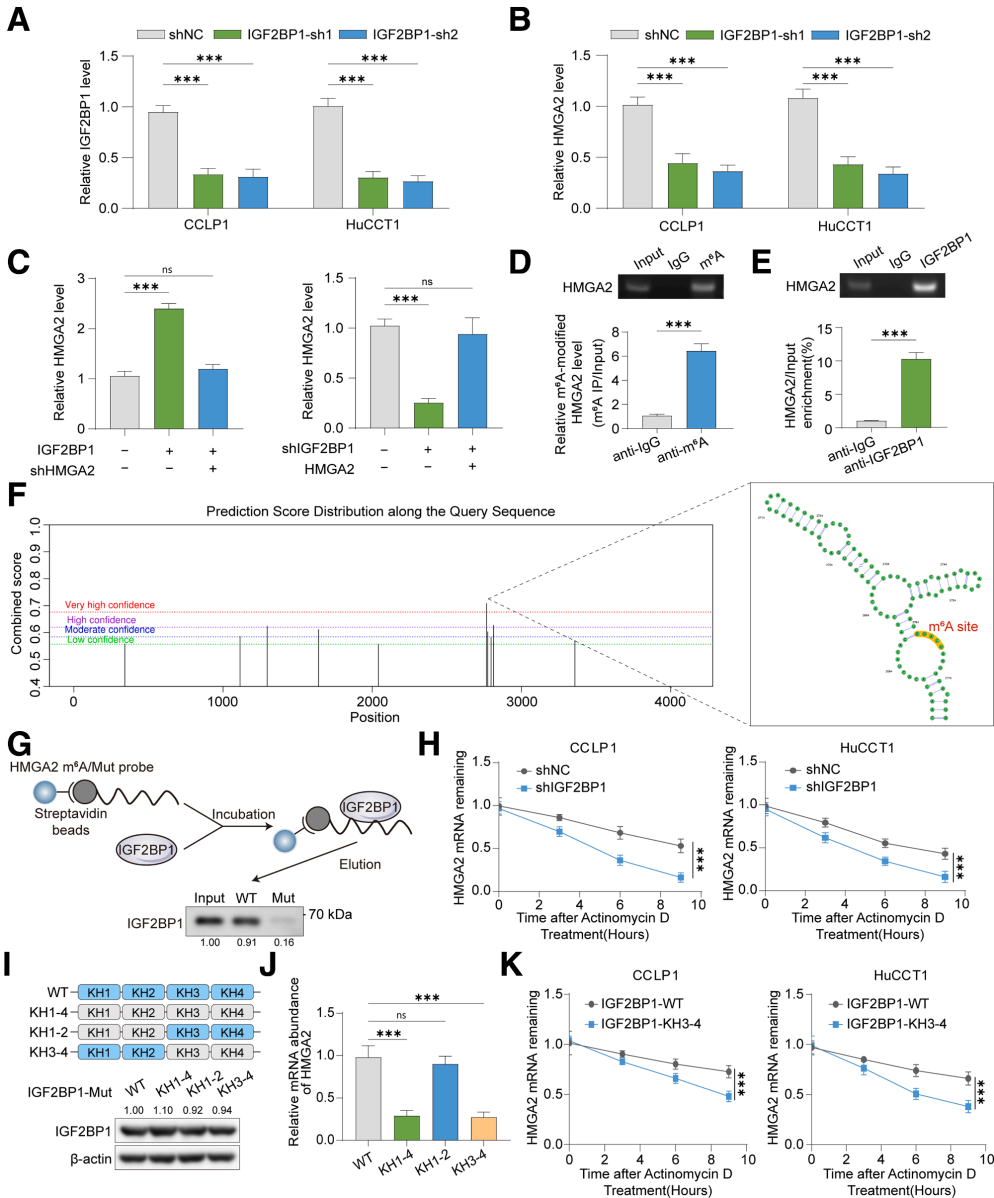
**IGF2BP1 enhances HMGA2 mRNA stability by recognizing and binding to its m^6^A site in iCCA.** (*A* and *B*) The mRNA levels of IGF2BP1 (*A*) and HMGA2 (*B*) in IGF2BP1-knockdown iCCA cells were detected by qRT-PCR. (*C*) Rescue experiments detecting the HMGA2 expression levels after cotransfection with IGF2BP1 silencing and HMGA2 overexpression or IGF2BP1 overexpression and HMGA2 silencing in iCCA cells assayed by qRT-PCR. (*D*) MeRIP followed by qRT-PCR showed enrichment of m^6^A-modified HMGA2 mRNA by the anti-m^6^A antibody in CCLP1 cells. (*E*) RIP followed by qRT-PCR showed binding of the HMGA2 with anti-IGF2BP1 in CCLP1 cells. (*F*) Cuilab (http://www.cuilab.cn/) online platform showing the predicted potential m^6^A binding sites of HMGA2 and the most reliable m^6^A binding site pattern diagram. (*G*) RNA pull-down followed by Western blot showed in vitro binding of the HMGA2-WT and HMGA2-Mut probes with IGF2BP1 protein in CCLP1 cells. (*H*) The HMGA2 mRNA half-life by silencing IGF2BP1 in CCLP1 and HuCCT1 cells. (*I*) *Upper panel*: Schematic structures showing RNA binding domains within IGF2BP1 protein and a summary of IGF2BP1 variants used in this study (*blue*, WT; *gray*, mutated sequences). *Lower panel*: Western blot analysis confirming protein expression of WT and Mut IGF2BP1 constructs. (*J*) The expression of HMGA2 in CCLP1 cell with forced expression of WT or mutated IGF2BP1 variants by qRT-PCR. (*K*) The HMGA2 mRNA half-life by forced expression of WT or the KH3-4–mutated IGF2BP1 variant in CCLP1 and HuCCT1 cells. All results are shown as mean ± standard deviation (n = 3). The Student *t* test or one-way/two-way analysis of variance was used to analyze the data; ∗∗*P* < .01; ∗∗∗*P* < .001; ns: not significant.

To validate whether HMGA2 is a direct m^6^A-modified target of IGF2BP1, we first evaluated the regulatory effect of IGF2BP1 on HMGA2 expression by depleting IGF2BP1 in CCLP1 and HuCCT1 cells (Figure 10*A*). IGF2BP1 knockdown significantly reduced HMGA2 mRNA levels in both cell lines (Figure 10*B*). Functional rescue experiments showed that HMGA2 re-expression restored transcript levels in IGF2BP1-depleted cells, whereas HMGA2 knockdown reversed elevated expression in IGF2BP1-overexpressing cells (Figure 10*C*). Critically, m^6^A-RNA immunoprecipitation (MeRIP) and RNA immunoprecipitation (RIP) assays confirmed direct binding of IGF2BP1 to m^6^A-modified HMGA2 mRNA (Figure 10*D* and *E*).

Using the SRAMP database, we designed biotin-labeled wild-type (HMGA2-WT) and matched mutant (HMGA2-Mut) RNA probes. The HMGA2-WT probe contained a single m^6^A site (GGACU) predicted as a very high-confidence site (Figure 10*F*). RNA pull-down assays demonstrated specific enrichment of IGF2BP1 by the HMGA2-WT probe but not the mutant, validating the recognition of m^6^A-modified HMGA2 by IGF2BP1 (Figure 10*G*). IGF2BP1 depletion significantly shortened HMGA2 mRNA half-life, confirming its role in transcript stabilization (Figure 10*H*).

To identify the key IGF2BP1 domains for m^6^A recognition, we generated KH domain mutants (KH1–4, KH1–2, KH3–4) as described previously.^31^, ^32^, ^33^ Western blot analysis confirmed that full-length IGF2BP1 and various KH domain mutants (KH1–4, KH1–2, KH3–4) displayed equivalent protein abundance in transfected iCCA cells (Figure 10*I*). Quantitative real-time polymerase chain reaction (qRT-PCR) analysis revealed that KH1–4 and KH3–4 mutants significantly decreased HMGA2 mRNA levels compared with WT IGF2BP1, whereas KH1–2 had no effect (Figure 10*J*). Actinomycin D-based RNA stability assays further showed that the KH3–4 mutant accelerated HMGA2 mRNA decay (Figure 10*K*). Collectively, these results demonstrate that the KH3–4 domains are essential for IGF2BP1-mediated m^6^A recognition and HMGA2 mRNA stabilization.

### IGF2BP1 is Involved in HMGA2–Mediated iCCA Progression

**Figure 11:**
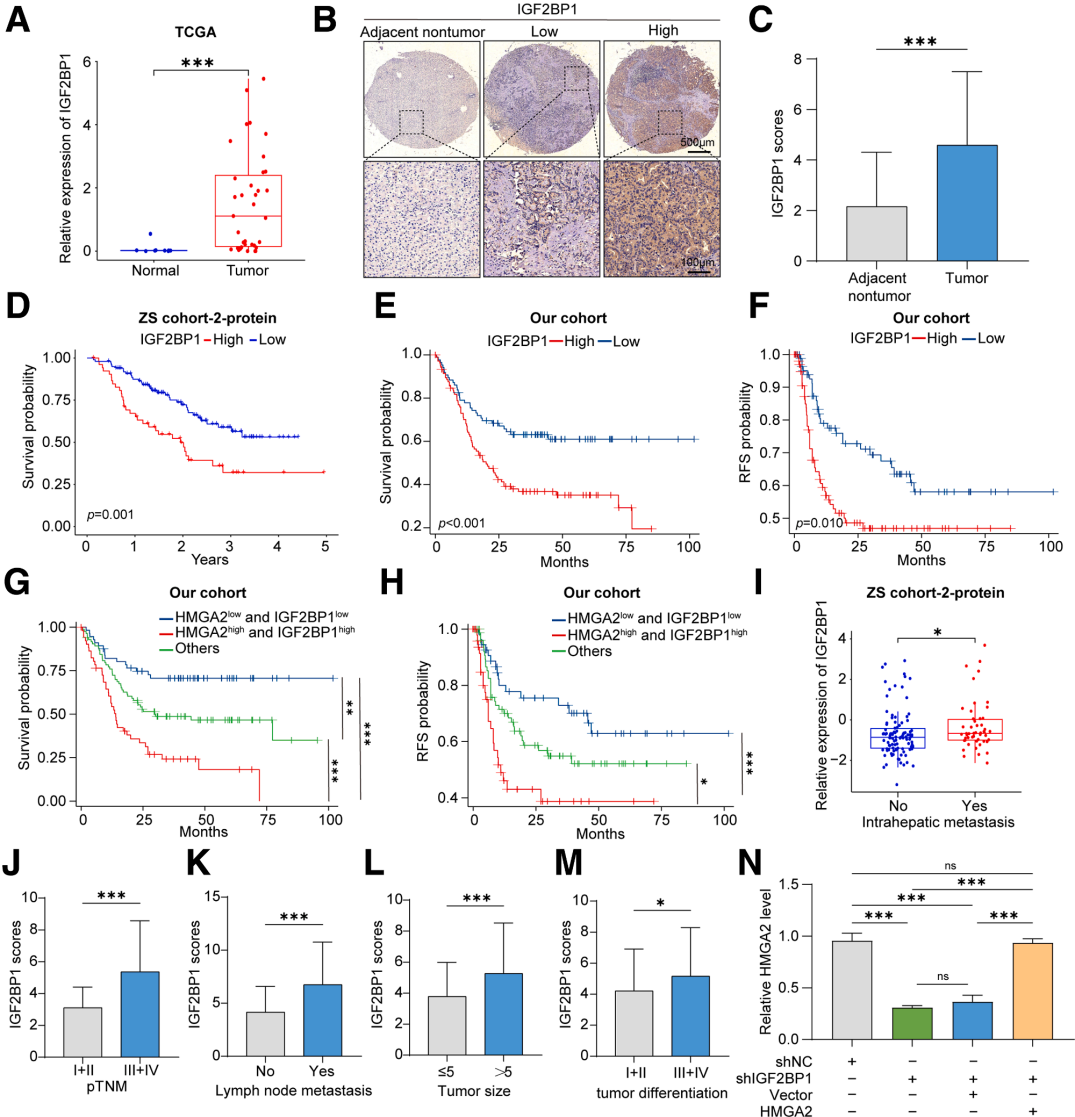
**Elevated IGF2BP1 expression correlates with poor clinical outcomes and malignant phenotypes in iCCA.** (*A*) Comparison of IGF2BP1 expression levels between tumor samples and adjacent normal controls in TCGA-CHOL database. (*B*) Representative pictures of IHC staining of in IGF2BP1 iCCA tissues and matched adjacent nontumor tissues (N = 192). (*C*) IHC scores of IGF2BP1 in our cohort. (*D*) OS analysis of patients with different IGF2BP1 protein expression levels in the ZS cohort-2. (*E–H*) Kaplan–Meier analysis of OS and RFS based on IGF2BP1 expression (*E–F*) and HMGA2/IGF2BP1 combinations (*G* and *H*) in our cohort, compared by the log-rank test. (*I*) The difference of IGF2BP1 expression between intrahepatic metastasis and no-metastasis groups in the ZS cohort-2. (*J–M*) The difference of IGF2BP1 expression in relation to pathologic TNM (pTNM) stage (*J*), lymph node metastasis (*K*), tumor size (*L*), and tumor differentiation (*M*) statuses in our cohort. (*N*) The mRNA levels of HMGA2 in HuCCT1 cells (shNC, shIGF2BP1, shIGF2BP1+Vector, and shIGF2BP1+HMGA2) were detected by qRT-PCR. The Student *t* test or one-way/two-way analysis of variance was used to analyze the data; ∗*P* < .05; ∗∗*P* < .01; ∗∗∗*P* < .001; ns: not significant.

Mounting evidence indicates that IGF2BP1, an RNA binding protein, is frequently upregulated in multiple cancers and strongly associated with poor clinical outcomes, including accelerated tumor progression, metastasis, and therapy resistance.^34^^,^^35^ To evaluate the clinical relevance of IGF2BP1 in iCCA, we analyzed its expression using the TCGA dataset and our independent patient cohort. IGF2BP1 was consistently overexpressed in iCCA across both datasets (Figure 11*A–C*). Survival analysis in the ZS cohort-2 demonstrated that elevated IGF2BP1 expression correlated with significantly shorter OS (Figure 11*D*). Consistently, high IGF2BP1 expression in our cohort predicted worse OS and RFS (Figure 11*E* and *F*). Notably, patients with concurrent high expression of IGF2BP1 and HMGA2 exhibited the most aggressive disease course, with the shortest OS and RFS among all subgroups (Figure 11*G* and *H*).

Analysis of clinicopathologic correlations revealed significantly higher IGF2BP1 expression in patients with iCCA and intrahepatic metastases vs nonmetastatic cases (ZS cohort-2) (Figure 11*I*). In our cohort, elevated IGF2BP1 levels correlated with multiple aggressive clinicopathologic features (Figure 11*J–M*, Table 2). These features included advanced pathologic TNM stage (III/IV vs I/II), lymph node metastasis, larger tumor size (>5 cm vs ≤5 cm), and poor tumor differentiation (III/IV vs I/II). These findings highlight a strong association between IGF2BP1 overexpression and malignant progression in iCCA.

**Table 2:** Comparison of Clinicopathologic Profiles Between Low and High IGF2BP1 Expression in Our Cohort (N = 192)

Variable	IGF2BP1 expression		*P*
	Low (n = 86)	High (n = 106)	
Gender			
Female	32	46	.385
Male	54	60	
Age, *y*			
≥50	70	85	.833
<50	16	21	
HBsAg			
Negative	55	74	.390
Positive	31	32	
HBcAb			
Positive	56	74	.489
Negative	30	32	
ALT			
≤75	63	81	.615
>75	23	25	
Tbil			
≤17.1	63	72	.421
>17.1	23	34	
GGT			
≤60	45	49	.401
>60	41	57	
Tumor size, *cm*			
≤5	47	41	**.027**
>5	39	65	
Tumor number			
Single	83	95	.068
Multiple	3	11	
Tumor differentiation			
I + II	55	61	.367
III + IV	31	45	
pTNM			
I + II	39	27	**.004**
III + IV	47	79	
Regional lymph node metastasis			
Negative	74	86	.364
Positive	12	20	

NOTE. Boldface *P* values indicate statistical significance. All categorical comparisons were performed using the χ² test.

ALT, alanine aminotransferase; GGT, gamma-glutamyl transferase; HbcAb, hepatitis B core antibody; HBsAg, hepatitis B surface antigen; IGF2BP1, insulin-like growth factor 2 messenger RNA-binding protein 1; pTNM, pathologic TNM; Tbil, total bilirubin.

**Figure 12:**
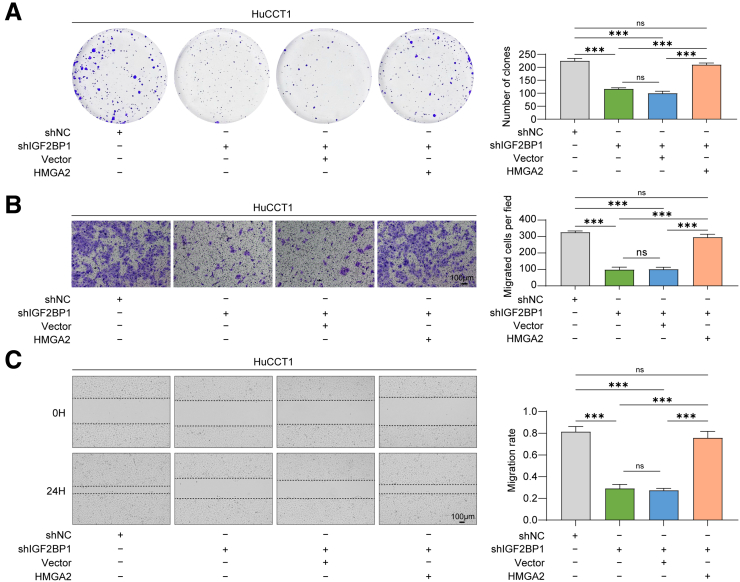
**The IGF2BP1–HMGA2 axis drives iCCA cell clonogenicity and migration.** (*A–C*) Colony-forming assays (*A*), cell migration ability (*B*), and wound-healing assays (*C*) after stably cotransfected with shNC, shIGF2BP1, shIGF2BP1+Vector and shIGF2BP1+HMGA2 in HuCCT1 cells; all results are shown as mean ± standard deviation (n = 3). One-way analysis of variance was used to analyze the data; ∗∗∗*P* < .001; ns: not significant.

To validate IGF2BP1 as the functional mediator of HMGA2-driven tumor progression, we performed rescue experiments by overexpressing HMGA2 in IGF2BP1-knockdown iCCA cells. HMGA2 re-expression reversed the inhibitory effects of IGF2BP1 depletion on colony formation (Figure 11*N*, and Figure 12*A*), and migration (Figure 12*B* and *C*).

Collectively, these data demonstrate that high IGF2BP1 expression predicts poor prognosis in iCCA and is essential for HMGA2-dependent tumor progression.

### The IGF2BP1–HMGA2 Axis Drives the Progression of iCCA

**Figure 13:**
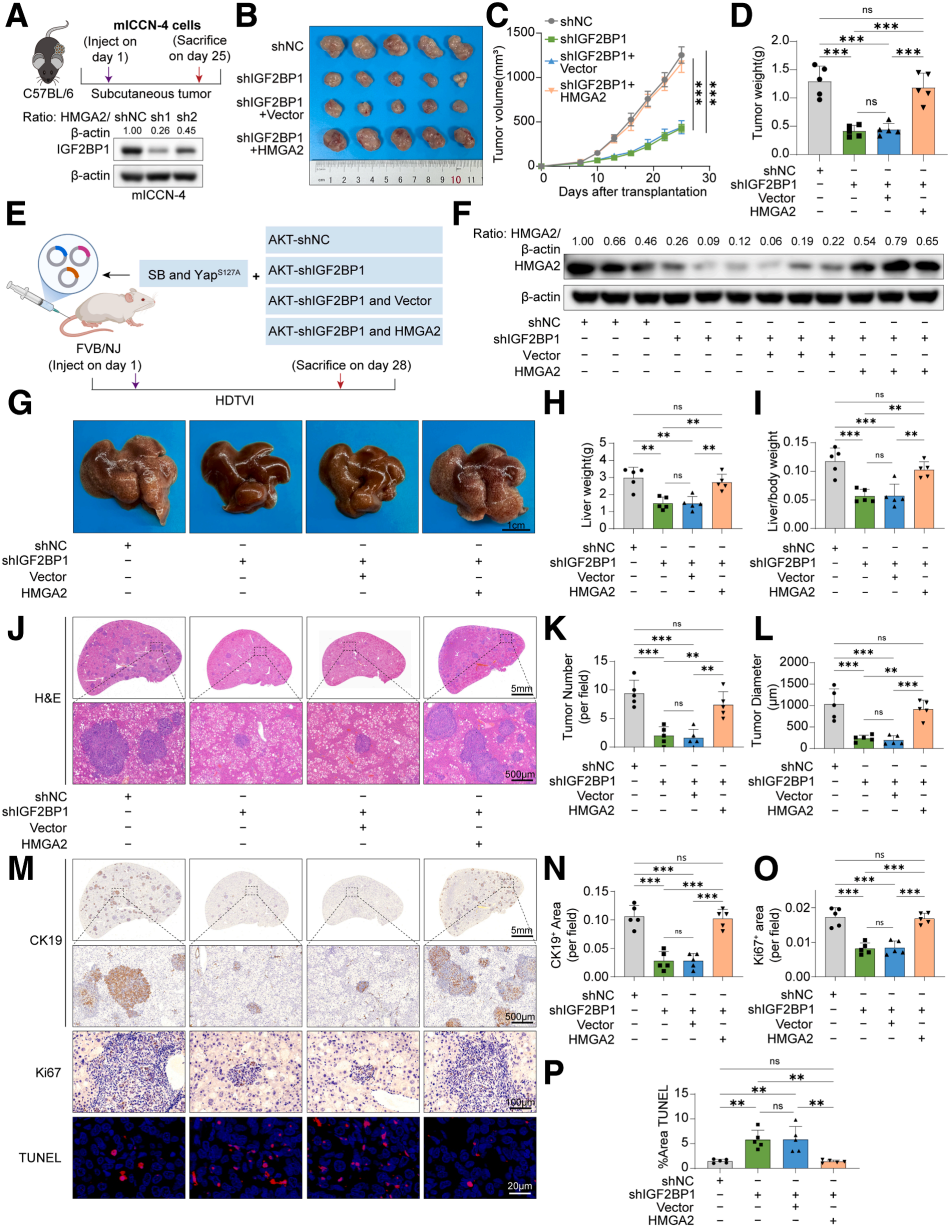
**The IGF2BP1–HMGA2 stabilization axis drives iCCA progression in subcutaneous tumor and hydrodynamic models.** (*A*) *Upper*: Diagram of the study design in the subcutaneous tumor mouse model (n = 5). *Lower*: The protein level of IGF2BP1 knockdown was confirmed by Western blotting in mICCN-4 cells. (*B*) Overview of tumors in C57 mice subcutaneously implanted with mICCN-4 cells that were stably cotransfected with shNC, shIGF2BP1, shIGF2BP1+PCDH and shIGF2BP1+HMGA2 (n = 5). (*C* and *D*) The tumor growth curves (*C*) and tumor weights (*D*) in C57 mice implanted with mICCN-4 cells that were stably cotransfected with shNC, shIGF2BP1, shIGF2BP1+PCDH and shIGF2BP1+HMGA2. (*E*) Diagram of the study design in the HDTVI mouse model (n = 5). (*F*) Western blot of the expression levels of HMGA2 in shNC, shIGF2BP1, shIGF2BP1+Vector and shIGF2BP1+HMGA2 mouse tumor tissues. (*G*) Representative images of shNC, shIGF2BP1, shIGF2BP1+Vector and shIGF2BP1+HMGA2 mouse livers. (*H* and *I*) Liver weight (*H*) and ratios of liver weight to body weight (*I*) in shNC, shIGF2BP1, shIGF2BP1+Vector and shIGF2BP1+HMGA2 mice. (*J–L*) Representative H&E-staining images of livers (*J*) and quantitative analysis of tumor number (*K*) and diameter (*L*) in shNC, shIGF2BP1, shIGF2BP1+Vector and shIGF2BP1+HMGA2 mice based on H&E staining. (*M–P*) Representative IHC staining of CK19, Ki67, and fluorescence staining of TUNEL in shNC, shIGF2BP1, shIGF2BP1+Vector and shIGF2BP1+HMGA2 mouse livers (*M*) and quantitative analysis of CK19^+^ area (*N*), Ki67^+^ area (*O*), and TUNEL^+^ area (*P*). All results are shown as mean ± standard deviation (n = 5). One-way/2-way analysis of variance was used to analyze the data, ∗∗*P* < .01; ∗∗∗*P* < .001; ns: not significant.

To further determine whether IGF2BP1 is required for HMGA2-mediated iCCA progression, we examined the effect of IGF2BP1 depletion on HMGA2-driven tumor growth and progression in vivo. We established 4 groups of murine mICCN-4 cells: IGF2BP1-knockdown (shIGF2BP1), rescue (shIGF2BP1+HMGA2), and 2 control groups (shNC, shIGF2BP1+Vector). Cells were subcutaneously inoculated into C57BL/6 mice (Figure 13*A*). IGF2BP1 knockdown significantly suppressed tumor growth, resulting in reduced final tumor weight and volume at the endpoint. This effect was reversed by HMGA2 reintroduction, which restored tumor growth to control levels (Figure 13*B–D*).

Furthermore, we established a spontaneous iCCA model in FVB/NJ mice via HDTVI. Injected plasmids encoded SB transposase, Yap^S127A^, HMGA2 (or empty vector), and AKT constructs harboring either IGF2BP1-targeting shRNA (AKT-shIGF2BP1) or control shRNA (AKT-shNC) (Figure 13*E*). Western blotting confirmed HMGA2 expression levels in mouse liver tissues (Figure 13*F*). At 4 weeks postinjection, mice in the shNC group developed substantial tumors. Conversely, AKT-shIGF2BP1 mice showed minimal hepatic tumor lesions (Figure 13*G*). This was accompanied by significantly reduced tumor burden, as reflected by decreased liver weight and liver-to-body weight ratio (Figure 13*H* and *I*). They also exhibited fewer and smaller tumor foci in H&E staining (Figure 13*J–L*), decreased CK19- and Ki67-positive areas (Figure 13*M–O*), and increased TUNEL-positive area (Figure 13*M* and *P*). Notably, all these phenotypic alterations were effectively rescued in the AKT-shIGF2BP1+HMGA2 group (Figure 13*G–P*). This confirmed that HMGA2 restoration rescues the tumor-suppressive effects of IGF2BP1 depletion.

Collectively, these in vivo rescue experiments demonstrate that the IGF2BP1-HMGA2 axis drives the progression of iCCA.

### The IGF2BP1–HMGA2 Axis Promotes Activation of the PI3K–AKT Signaling Pathway in iCCA

**Figure 14:**
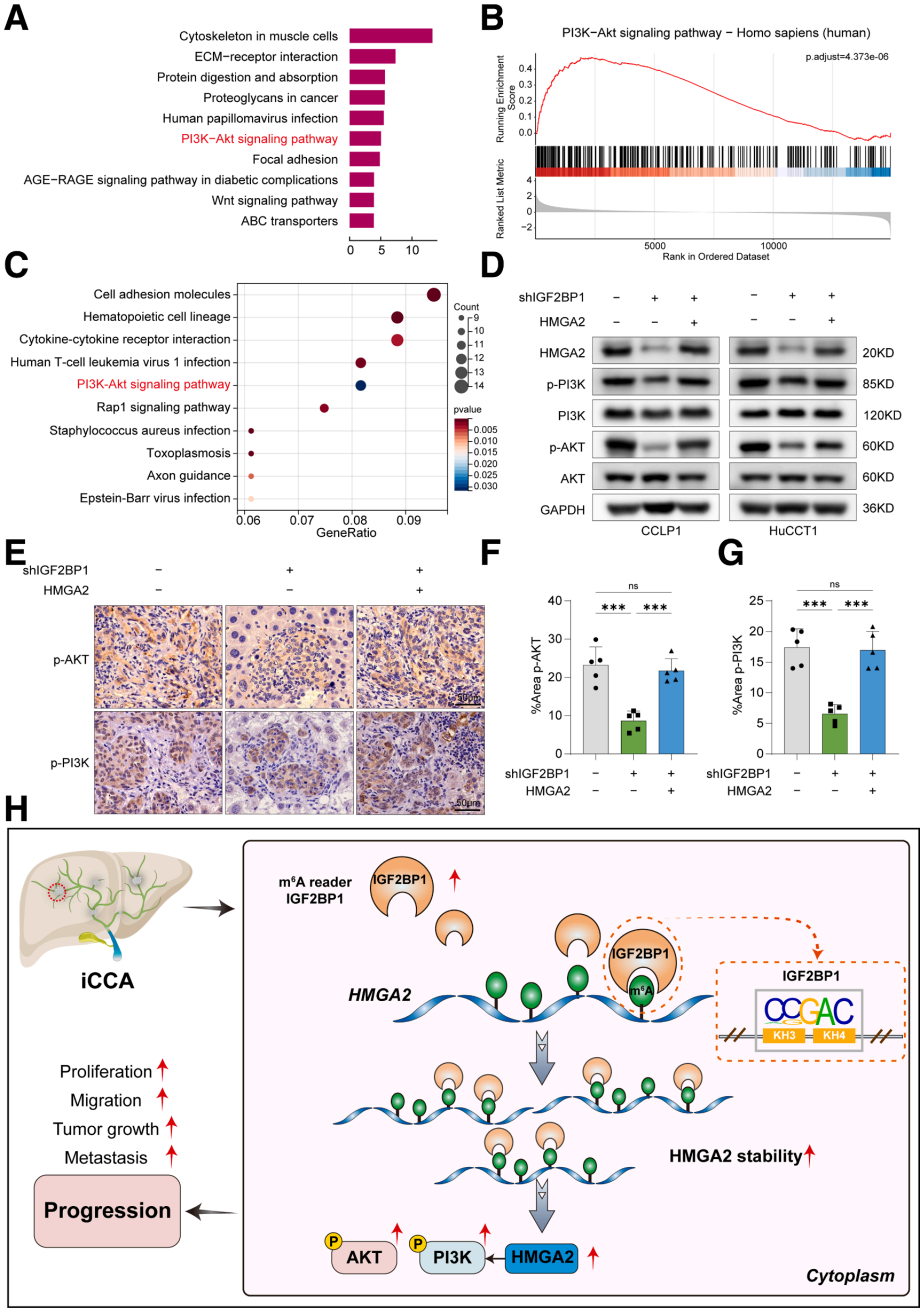
**The IGF2BP1–HMGA2 axis promotes activation of the PI3K-AKT signaling pathway during iCCA progression.** (*A*) KEGG pathway enrichment analysis of DEGs, displaying the top 10 significantly enriched pathways from TCGA dataset. (*B*) Gene Set Enrichment Analysis (GSEA) analysis of the PI3K-Akt signaling pathway in TCGA dataset. (*C*) KEGG pathway enrichment analysis of DEGs, displaying the top 10 significantly enriched pathways from our RNA-seq dataset. (*D*) Western blot analysis of HMGA2, phosphorylated/total PI3K, and phosphorylated/total AKT protein levels in CCLP1 and HuCCT1 cells following IGF2BP1 knockdown and/or HMGA2 overexpression. (*E–G*) Representative IHC staining of p-AKT and p-PI3K in shNC, shIGF2BP1, and shIGF2BP1+HMGA2 mouse livers (*E*) and quantitative analysis of p-AKT–positive area (*F*), and p-PI3K–positive area (*G*) from the HDTVI mouse model. Scale bars, 50 μm. (*H*) Schematic model illustrating the mechanism: IGF2BP1 recognizes m^6^A-modified HMGA2 mRNA via its KH3-4 domains to enhance HMGA2 stability, which in turn promotes activation of the PI3K-AKT pathway during iCCA progression. All results are shown as mean ± standard deviation (n = 5). One-way analysis of variance was used to analyze the data, ∗∗∗*P* < .001; ns: not significant.

To further explore the downstream mechanisms of the IGF2BP1–HMGA2 axis in iCCA progression, we first analyzed the TCGA dataset. We stratified TCGA-CHOL samples into high- and low-HMGA2 expression groups and performed differential gene expression analysis. Kyoto Encyclopedia of Genes and Genomes (KEGG) enrichment analysis was performed on the identified differentially expressed genes (DEGs), with the top 10 enriched pathways visualized in Figure 14*A* and *B*. In parallel, we performed RNA sequencing (RNA-seq) in HMGA2-knockdown and matched control HuCCT1 cells. KEGG enrichment was conducted on the resulting DEGs, with the top 10 pathways presented in Figure 14*C*. PI3K-AKT signaling was significantly enriched in both the TCGA cohort and our RNA-seq dataset. This pathway is a well-established core oncogenic driver of iCCA and multiple malignancies,^36^, ^37^, ^38^ and we thus selected it for further mechanistic validation.

Western blotting analysis showed that IGF2BP1 knockdown significantly downregulated the protein levels of HMGA2, phosphorylated PI3K (p-PI3K) and phosphorylated AKT (p-AKT) in both CCLP1 and HuCCT1 cells. Conversely, restoration of HMGA2 expression in IGF2BP1-silenced cells markedly elevated the protein levels of HMGA2, p-PI3K, and p-AKT (Figure 14*D*). Consistent with these in vitro findings, we performed IHC staining and quantitative analysis using the YAP/AKT-driven spontaneous iCCA mouse model. IGF2BP1 knockdown significantly reduced the positive staining areas of p-PI3K and p-AKT, and this alteration was effectively reversed by HMGA2 overexpression (Figure 14*E–G*). Collectively, these data demonstrated that the IGF2BP1–HMGA2 axis promotes activation of the PI3K-AKT signaling pathway during iCCA malignant progression, and this pathway may serve as a key downstream cascade mediating the oncogenic effects of this axis.

In conclusion, HMGA2 and IGF2BP1 are significantly upregulated in iCCA and predict adverse patient prognosis. Functionally, as a key oncogene, HMGA2 drives iCCA cell proliferation and migration in vitro, as well as in vivo tumor growth and metastasis. Mechanistically, the m^6^A reader IGF2BP1 binds m^6^A-modified HMGA2 mRNA via its KH3–4 domains to enhance transcript stability, and the IGF2BP1-HMGA2 axis promotes activation of the PI3K-AKT signaling pathway in iCCA progression (Figure 14*H*). Collectively, our findings establish this axis as a novel prognostic biomarker and promising therapeutic target for iCCA.

## Discussion

Accumulating evidence firmly establishes HMGA2 as a key oncogenic driver across multiple malignancies, driving invasion, metastasis, and therapy resistance through diverse mechanisms.^9^^,^^39^ In colorectal cancer, HMGA2 promotes progression via USP48-dependent stabilization^40^ and STAT3-mediated tumor-associated macrophage recruitment.^14^ In pancreatic ductal adenocarcinoma, it enhances LIN28B downstream mRNA translation to drive disease pathogenesis.^13^ Despite these advances, the molecular functions of HMGA2 in iCCA remain poorly defined. Here, we show that HMGA2 is markedly upregulated in iCCA and correlates with advanced disease and poor patient survival. Our results are consistent with a previous cohort study reporting HMGA2 as an independent prognostic marker in iCCA.^17^ Critically, we provide the first functional evidence that HMGA2 knockdown suppresses iCCA progression both in vitro and in vivo, highlighting its therapeutic potential.

m^6^A is a critical post-transcriptional regulator of cancer gene expression.^28^^,^^29^^,^^41^ As a core m^6^A reader, IGF2BP1 stabilizes oncogenic transcripts to promote tumorigenesis.^42^ Through bioinformatic screening and clinical validation, we identified IGF2BP1 as an upstream regulator of HMGA2 in iCCA. Consistent with previous reports,^23^ IGF2BP1 was significantly overexpressed in iCCA tissues and associated with adverse clinical outcomes. Notably, we observed a significant positive correlation between IGF2BP1 and HMGA2 expression in our patient cohort, a regulatory relationship not previously characterized in iCCA. Mechanistically, IGF2BP1 directly binds the m^6^A-modified HMGA2 mRNA and enhances its stability. Rescue experiments confirmed that IGF2BP1 exerts oncogenic functions via HMGA2 upregulation. These findings define a novel m^6^A-dependent IGF2BP1-HMGA2 axis driving iCCA progression.

Although our findings uncover novel mechanistic insights into iCCA progression, several limitations warrant consideration. Functional investigations relied on constitutive knockdown or overexpression in established iCCA cell lines, which may not fully recapitulate dynamic gene regulation during native iCCA progression. Although key findings were validated across multiple mouse models—including xenografts, syngeneic tumors, and spontaneous iCCA models—these preclinical models cannot fully mimic human iCCA’s genetic heterogeneity and tumor microenvironment. Further validation using patient-derived organoids or primary human iCCA cultures would better mirror clinical disease characteristics and enhance the translational relevance of our results. We did not assess the pharmacologic targetability of the IGF2BP1–HMGA2 axis using small-molecule inhibitors; future preclinical studies are therefore needed to evaluate its therapeutic potential. Finally, our multicohort analyses are limited by inherent intercohort heterogeneity and technical batch effects, which may restrict the overall generalizability of our prognostic conclusions; in particular, the female-specific prognostic association between HMGA2 and RFS, as well as its link to PNI, requires further validation in larger, multicenter prospective cohorts to confirm its clinical applicability.

In conclusion, our study identifies HMGA2 as a key oncogenic driver in iCCA. HMGA2 and its upstream m^6^A regulator IGF2BP1 are significantly upregulated in iCCA and predict poor patient prognosis. Mechanistically, IGF2BP1 stabilizes HMGA2 mRNA via its KH3–4 domains, and the IGF2BP1-HMGA2 axis promotes activation of the PI3K-AKT signaling pathway during iCCA progression (Figure 14*H*). Collectively, our findings establish this axis as a novel prognostic biomarker and promising therapeutic target for iCCA.

## Materials and Methods

### Patients and Follow-Up

A total of 192 patients with a confirmed diagnosis of iCCA were enrolled in our cohort. After obtaining written informed consent, paired samples of tumor and adjacent nontumor liver tissues were collected from patients who underwent surgical resection of iCCA at the participating institutions. Immediately following collection, specimens were either snap-frozen in liquid nitrogen or fixed in 10% neutral buffered formalin and embedded in paraffin. None of the included patients had received neoadjuvant chemotherapy or radiotherapy prior to surgery. The study protocol received approval from the Ethics Committee of Fudan University.

### Tissue Microarrays of iCCA Tissues and Immunohistochemistry Assay

IHC staining was performed according to previously described methods.^43^ Briefly, following deparaffinization, rehydration, and antigen retrieval, tissue sections were incubated with primary antibodies at 4 °C overnight, followed by incubation with a secondary antibody (Dako Denmark A/S) at 37 °C for 30 minutes. TMA blocks were constructed using tumor and matched adjacent nontumor tissues from 192 patients with iCCA. Two 4-μm core biopsies from each donor block were transferred to recipient paraffin blocks at predefined array positions. Detailed information for all antibodies is provided in Table 3.

**Table 3:** Details of Antibodies Used in This Study

Antibodies	Commercial sources	Catalog no.
HMGA2	Cell Signaling Technology	8179
IGF2BP1	Abcam	ab290736/ab313422
CK19	Proteintech	10712-1-AP
Ki67	Cell Signaling Technology	34330
AKT	Cell Signaling Technology	4691
p-AKT	Cell Signaling Technology	4060
PI3K	Cell Signaling Technology	4249
p-PI3K	Invitrogen	PA5-104853
GAPDH	Proteintech	60004-1-Ig
β-actin	Proteintech	66009-1-Ig

The staining results for HMGA2 and IGF2BP1 were evaluated using a semiquantitative scoring system based on the product of the percentage of positive cells and staining intensity. The percentage of positive cells was scored as follows: 0 (0%–5%), 1 (6%–25%), 2 (26%–50%), 3 (51%–75%), or 4 (75%–100%). Staining intensity was graded as: 0 (none), 1 (weak), 2 (moderate), or 3 (strong). Based on the total score, all specimens were divided into 2 groups: the low-expression group (total score: 0–3) and the high-expression group (total score: 4–12).

### N^6^-Methyladenosine–RNA Immunoprecipitation Assay

Total RNA isolated from CCLP1 cells was chemically fragmented to ∼100 nt fragments. Following the EpiQuick CUT&RUN m^6^A RNA Enrichment kit protocol (P-9018, Epigentek), 10 μg of fragmented mRNA per sample was incubated with 2 μg anti-m^6^A antibody (or control IgG) and 4 μL Affinity Beads for 1.5 hours at 25 °C with rotation. The mixture was then treated with 10 μL Nuclear Digestion Enhancer and 2 μL Cleavage Enzyme Mix (4 minutes, 25 °C), followed by proteinase K digestion and RNA elution. The immunoprecipitated RNA underwent m^6^A-enriched mRNA profiling via high-throughput sequencing and was validated by qRT-PCR using primers specified in Table 4.

**Table 4:** Sequences of Primers, shRNAs, and Overexpression Plasmid

Primers used in qRT-PCR analysis		
Name	Forward primer	Reverse primer
HMGA2	ACCCAGGGGAAGACCCAAA	CCTCTTGGCCGTTTTTCTCCA
IGF2BP1	GGCCATCGAGAATTGTTGCAG	CCAGGGATCAGGTGAGACTG
β-actin	GGCTGTATTCCCCTCCATCG	CCAGTTGGTAACAATGCCATGT

Sequences of shRNAs, siRNAs, and overexpression plasmid	
Name	Sequences (5′-3′)
HMGA2-h-sh1	AGTCCCTCTAAAGCAGCTCAA
HMGA2-h-sh2	CCGAATTGGGTTTAGTCAATC
HMGA2-m-sh1	GCCACAACAAGTCGTTCAGAA
HMGA2-m-sh2	AGACCTAGGAAATGGCCACAA
IGF2BP1-h-sh1	ACGCTTAGAGATTGAACATTC
IGF2BP1-h-sh2	CTCCAAAGTTCGTATGGTTAT
IGF2BP1-m-sh1	GAAACACCTGACTCCAAAGTT
IGF2BP1-m-sh2	CGACCAAGTCATTGTTAAGAT
HMGA2-h-si#1	AGAUUGAGAUUGAAAGUGCCU
HMGA2-h-si#2	GCACUUUCAAUCUCAAUCU
HMGA2-h	NM_003483.6
HMGA2-m	NM_010441.3
IGF2BP1-h	NM_006546.4

qRT-PCR, quantitative real-time polymerase chain reaction; shRNA, short hairpin RNA; siRNA, small interfering RNA.

### RNA Immunoprecipitation Assay

The RIP assay was performed using the Magna RIP RNA-Binding Protein Immunoprecipitation Kit (17-700, Merck Millipore) in strict accordance with the manufacturer's protocol. Briefly, iCCA cells were lysed with the provided RIP Lysis Buffer supplemented with protease and RNase inhibitors to maintain complex integrity and prevent RNA degradation. The lysate was then incubated overnight at 4 °C with protein A/G magnetic beads conjugated with 5 μg of anti-IGF2BP1 antibody or control IgG. Following extensive washing steps to remove nonspecifically bound materials, coprecipitated RNA was purified and analyzed via qRT-PCR using gene-specific primers listed in Table 4.

### Biotinylated RNA Pull-Down Assay

Biotinylated single-stranded RNA (ssRNA) probes were synthesized in vitro by Sangon Biotech (Shanghai) Co, Ltd. The WT probe was 45 nucleotides in length, designed based on the predicted very high-confidence m^6^A site from the SRAMP database, and contained a single m^6^A site (GGACU). The corresponding mutant probe carried an A-to-U substitution at this predicted m^6^A modification site. The oligonucleotide sequences are provided in Table 5. RNA-protein pull-down assays were performed using the Pierce Magnetic RNA-Protein Pull-Down Kit (Thermo) following the manufacturer’s protocol. Briefly, 100 picomoles of biotinylated ssRNA probes were bound to magnetic beads and incubated with cell lysates to capture RNA-binding proteins. After washing steps to remove nonspecifically bound materials, the retained complexes were eluted and detected by Western blot analysis using the anti-IGF2BP1 antibody.

**Table 5:** Sequences of ssRNA Probes

Name	Sequences (5′-3′)
WT probe	AAUGAAUUCUUUAAGGAGAUGGACUAAUUGACUUGCAAAGACCUA
Mut probe	AAUGAAUUCUUUAAGGAGAUGGUCUAAUUGACUUGCAAAGACCUA

Mut, mutant; ssRNA, single-stranded RNA; WT, wild-type.

### RNA Stability Assay

CCLP1 or HuCCT1 cells were transfected with shNC (non-targeting control), shIGF2BP1 (targeting IGF2BP1), IGF2BP1-WT (wild-type), or IGF2BP1-KH34 (KH domains mutant) constructs using lipofectamine-based transfection reagents. Subsequently, the transfected cells were treated with actinomycin D (HY-17559, MCE) at a final concentration of 10 μg/mL for 0, 3, 6, and 9 hours. After treatment, cells were harvested, and total RNA was extracted using a commercial RNA isolation kit. The extracted RNA was then analyzed by qRT-PCR to evaluate mRNA expression levels using primers listed in Table 4.

### Bioinformatics Analysis

Gene expression and clinical data were collected from multiple independent iCCA/CCA cohorts. Fragments per kilobase of transcript per million mapped read–formatted transcriptomic and survival data for TCGA-CHOL were obtained from the UCSC Xena platform, with clinical annotations supplemented using the R package “TCGAbiolinks.” We also included 2 Zhongshan Hospital cohorts: ZS cohort-1 (Lin et al^44^), comprising 207 tumor transcriptomes from 45 patients with iCCA; and ZS cohort-2 (Dong et al^45^), comprising 255 tumor transcriptomes and 214 tumor proteomes from 262 patients with iCCA, both with complete prognostic information. The GSE45001 dataset was acquired from the Gene Expression Omnibus database, and Visium spatial transcriptomic data of iCCA were obtained from Wu et al.^24^

All bulk transcriptomic data were converted from fragments per kilobase of transcript per million mapped read to transcripts per million and log_2_(x+1)-transformed to reduce the influence of outliers. Proteomic data from Dong et al^45^ were used directly as provided by the authors without further processing. All bioinformatic and statistical analyses were performed in R (v4.2.2) and Python (v3.9.18). Univariate Cox regression analysis was performed using the “coxph” function in the R package survival to estimate the prognostic value of each gene for OS, RFS, and DSS in the TCGA and Zhongshan Hospital cohorts. Genes with *P* < .01 and hazard ratio >1 were defined as candidate risk factors.

Spatial expression patterns of HMGA2 were visualized using Visium spatial transcriptomic data. Tumor and normal tissue regions were delineated by an experienced pathologist on the basis of H&E staining. Spots with HMGA2 expression were defined as HMGA2-positive, whereas those with undetectable expression were categorized as HMGA2-negative. χ^2^ tests were subsequently performed to compare the proportions of HMGA2-positive and HMGA2-negative spots between tumor and normal regions.

For survival analysis stratification, the optimal expression cutoffs for HMGA2 and IGF2BP1 were determined using the “surv_cutpoint” function in the R package “survminer,” and samples were then divided into high- and low-expression subgroups. Kaplan–Meier survival analyses were performed for OS, RFS, and DSS in the TCGA cohort, ZS cohort-1, ZS cohort-2, and our in-house iCCA cohort, with differences between curves assessed by the log-rank test.

For pathway enrichment analysis, tumor samples from the TCGA-CHOL cohort were dichotomized into high and low HMGA2 expression groups by median expression level. Genes with <30 counts in over 50% of samples were excluded to filter low-expression transcripts. Differential expression analysis was performed using the R package “limma,” with thresholds of |log_2_FC| > 1 and *P*-value < .05. KEGG pathway enrichment analysis of the identified DEGs was conducted using the R package “clusterProfiler.” In addition, Gene Set Enrichment Analysis was performed via the gseKEGG function on the full gene expression profile. This analysis further validated the association between HMGA2 expression and key oncogenic KEGG pathways.

### Cell Culture

Human cholangiocarcinoma cell lines (CCLP1, HuCCT1, and RBE) and human intrahepatic biliary epithelial cells (HIBEpic) were cultured following previously established protocols.^46^ The murine cholangiocarcinoma cell line mICCN-4, which exhibits stable proliferative characteristics and tumorigenic capacity in vivo, was generously provided by the research group of Professor Qin Lunxiu at Huashan Hospital, Fudan University. This cell line has been granted a national invention patent (Patent No.: CN202311094740.1). All cells were maintained in Dulbecco’s Modified Eagle Medium (DMEM) medium supplemented with 10% fetal bovine serum (FBS), 1% penicillin/streptomycin, and incubated at 37 °C under 5% CO_2_. All functional assays were performed using cells between passages 3 and 8 to minimize phenotypic drift and ensure experimental consistency. Cells were digested, counted, and seeded for functional assays when they reached 70% to 80% confluence (logarithmic growth phase).

### Cell Proliferation and Colony Formation Assays

Cell proliferation was assessed using the Cell Counting Kit-8 (CCK-8; Dojindo). Briefly, 1 × 10^3^ iCCA cells were seeded per well in 96-well plates. At designated time points, the culture medium was replaced with a mixture of 10 μL CCK-8 reagent and 90 μL complete medium per well. After incubation at 37 °C under 5% CO_2_ for 1 to 2 hours, absorbance was measured at 450 nm. For the colony formation assay, which measures clonogenic potential, 1 × 10^3^ treated or control iCCA cells were seeded per well in 6-well plates and cultured for 14 days. The resulting cell colonies were fixed with 4% formaldehyde and stained with crystal violet. The number of colonies was subsequently quantified and statistically analyzed using ImageJ software.

### Migration Assays

Cell migration was evaluated using 24-well Transwell chambers (Corning) equipped with 8-μm pore polycarbonate membranes. Briefly, 5 × 10^4^ iCCA cells resuspended in serum-free medium were seeded into the upper chamber, while the lower chamber was filled with DMEM containing 20% FBS as a chemoattractant. The chambers were incubated at 37 °C under 5% CO_2_ for the desired migration period. After incubation, cells that migrated to the underside of the membrane were fixed with 4% paraformaldehyde for 15 minutes and stained with 0.5% crystal violet solution for 30 minutes. Following gentle rinsing with phosphate-buffered saline (PBS) to remove excess dye, the migrated cells were imaged under a light microscope. Quantitative analysis was performed by counting cells in 3 to 5 random fields per membrane, and statistical analysis was based on 3 independent experiments with triplicate replicates.

### Wound Healing Assay

The cell migration capacity was assessed using a wound healing assay. Briefly, cells were seeded into 6-well plates and cultured until reaching 95% to 100% confluence. Wounds were introduced into the monolayer using a sterile 10-μL pipette tip. After 3 washes with PBS to remove detached cells, the cells were cultured in low-FBS medium. Following 24 hours of incubation, wound areas were imaged under an inverted microscope. The images were analyzed using ImageJ software, and the percentage of cell migration was calculated as: (Initial scratch area – Final scratch area) / Initial scratch area × 100%.

### Western Blot

Western blot analysis was performed following an established protocol.^43^ Total protein was extracted using RIPA lysis buffer supplemented with a protease and phosphatase inhibitor cocktail. Protein samples were separated by sodium dodecyl sulfate–polyacrylamide gel electrophoresis and subsequently transferred onto polyvinylidene fluoride membranes. The membranes were blocked with 5% skim milk in Tris-buffered saline containing 0.1% Tween-20 (TBST) for 1 hour at room temperature. Thereafter, they were incubated with specific primary antibodies—including HMGA2, IGF2BP1, AKT, p-AKT, PI3K, p-PI3K, GAPDH, and β-actin antibody—overnight at 4 °C, followed by incubation with the secondary antibody for 1 to 2 hours at room temperature. Protein bands were visualized using enhanced chemiluminescence substrate and imaged with an ImageQuant LAS 4000 system (GE Healthcare Life Sciences). Detailed information for all antibodies is provided in Table 3.

### RNA Extraction and Real-Time Quantitative Reverse Transcription Polymerase Chain Reaction

RNA was extracted according to previously established protocols.^43^^,^^47^ The purity and concentration of the isolated RNA were determined using the NanoDrop 2000 spectrophotometer. Subsequently, first-strand complementary DNA was synthesized from total RNA using the HiScript IV All-in-One Ultra RT SuperMix for qPCR (R433-01, Vazyme). qRT-PCR was performed with the Taq Pro Universal SYBR qPCR Master Mix (Q712-03, Vazyme) on an ABI PRISM 7900 Sequence Detection System (Applied Biosystems). Gene expression fold changes were calculated using the 2−ΔΔCt method, with β-actin serving as the endogenous control for normalization. The sequences of all primers used are provided in Table 4.

### Terminal Deoxynucleotidyl Transferase Mediated dUTP Nick-End Labeling Assay

Mouse iCCA tissues were fixed in 4% paraformaldehyde, embedded in paraffin, and sectioned at 5 μm. After dewaxing, rehydration and permeabilization, TUNEL staining was performed using the In Situ Cell Death Detection Kit (TMR red, Roche, Cat. No. 12156792910). TUNEL reaction mixture was freshly prepared and incubated with sections at 37 °C for 60 minutes in the dark. Fluorescent signals were detected under a fluorescence microscope. The percentage of apoptotic cells was quantified and statistically analyzed using the same method as IHC.

### Animal Experiments

Male C57BL/6 and BALB/c nude mice (5 weeks old) were supplied by Shanghai Slac Laboratory Animal Co, Ltd, while FVB/NJ mice (6–7 weeks old) were obtained from GemPharmatech Co, Ltd. All animals were maintained under specific pathogen-free conditions with ad libitum access to standard rodent diet and water. The experimental protocols were approved by the Animal Care and Use Committee of Fudan University.

For xenograft experiments, CCLP1 cells (shNC or shHMGA2; 2×10^6^ cells in 100 μL PBS) were subcutaneously injected into the right flank of BALB/c nude mice (n = 5 per group). Similarly, mouse mICCN-4 cells (including shNC, shHMGA2, shIGF2BP1, Vector, and HMGA2 groups; 5 × 10^6^ cells in 100 μL PBS) were inoculated into the right flank of C57BL/6 mice (n = 5 per group). At the end point, all tumors were excised and weighed. Tumor volume was calculated according to the formula: Volume = ½ × (length × width^2^), where length represents the longest tumor diameter and width the shortest diameter.

The HDTVI mouse model was established using male FVB/NJ mice (6–7 weeks old) in accordance with established protocols.^25^, ^26^, ^27^^,^^46^ A plasmid mixture contained 30 μg of pT3-EF1α-YapS127A, along with 20 μg of either pT3-EF1α-AKT-shHMGA2 or its control plasmid (pT3-EF1α-AKT-shNC). This mixture was combined with the SB transposase plasmid at a mass ratio of 1:10 to 1:25 relative to total DNA. The mixture was diluted in 2.0 mL of sterile saline and injected into the tail vein of FVB/NJ mice within 5–10 seconds using a high-volume syringe. In a separate group, 30 μg of pT3-EF1α-YapS127A was coinjected with 20 μg of either pT3-EF1α-AKT-shIGF2BP1 or its corresponding control, together with 20 μg of pT3-EF1α-HMGA2 or empty vector. SB transposase was included at the same ratio (1:10 to 1:25), and the mixture was prepared and injected as described above.

For the liver-specific HMGA2 overexpression HDTVI mouse model, we prepared a plasmid mixture containing 30 μg of pT3-EF1α-YapS127A, 20 μg of pT3-EF1α-AKT, and 20 μg of either pT3-EF1α-HMGA2 or empty vector. This mixture was combined with the SB transposase plasmid at the same mass ratio of 1:10 to 1:25 relative to total DNA. All surviving mice were humanely euthanized 4 weeks postinjection, after which livers were harvested, weighed, and processed for IHC analysis.

To establish the lung metastasis model, 2 × 10^6^ HuCCT1 cells stably transfected with shNC or shHMGA2 were resuspended in 0.2 mL PBS. Cells were injected via the tail vein into 5- to 6-week-old BALB/c nude mice (n = 5 per group). Five weeks later, mice were humanely euthanized, and lung tissues were harvested, fixed, dissected, and paraffin-embedded for H&E staining.

For the liver metastasis model, 5 × 10^6^ HuCCT1 cells (shNC and shHMGA2) resuspended in 100 μL PBS were injected into the splenic parenchyma of nude mice (n = 5 per group). The spleen was then resected to prevent tumor growth at the injection site. Similarly, 5 × 10^6^ murine mICCN-4 cells (shNC and shHMGA2) in 100 μL PBS were inoculated into the splenic parenchyma of C57BL/6 mice (n = 5 per group). Four weeks postinjection, mice were humanely euthanized, and liver tissues were harvested for H&E staining.

### Hematoxylin and Eosin and Immunohistochemical Staining

H&E staining and IHC were performed according to established methodologies.^43^ Briefly, mouse tissues were fixed in 10% neutral buffered formalin and embedded in paraffin. Sections were cut at 4- to 5-μm thickness, deparaffinized in xylene, and rehydrated through a graded ethanol series. For H&E staining, sections were stained with H&E, dehydrated through graded ethanol and xylene, and mounted with neutral balsam. For IHC, antigen retrieval was followed by blocking with 5% goat serum. Sections were incubated with anti-CK19 or anti-Ki67 at 4 °C overnight, then with the secondary antibody at 37 °C for 30 minutes. Detection was performed using 3,3'-diaminobenzidine substrate, followed by hematoxylin counterstaining.

Quantitative analyses were performed according to established methodologies.^48^ For tumor lesion quantification on H&E-stained sections: 3 nonoverlapping random low-power fields (100× magnification) were selected per sample. Tumor nodules were identified and enumerated based on typical malignant cytologic features, including cellular pleomorphism and nuclear hyperchromasia. Tumor diameter was defined as the longest axis of the largest tumor nodule within each selected field. Mean values for tumor number and tumor diameter were calculated from the 3 fields per sample. For CK19 and Ki-67 IHC quantification, 3 nonoverlapping random high-power fields (400× magnification) were acquired per section. Quantitative analysis was performed using ImageJ software, and the mean number of positive cells per field was calculated from the 3 selected fields.

### RNA Sequencing

HuCCT1 cells were transfected with shHMGA2 or nontargeting shNC. Total RNA was extracted using TRIzol reagent (Invitrogen), with purity and integrity verified via the NanoDrop ND-2000 and the Agilent 5300 Bioanalyzer. Qualified RNA was used for library construction following the standard Illumina protocol. After being quantified by the Qubit 4.0, 2 × 150 bp paired-end sequencing was performed on the Illumina NovaSeq X Plus sequencer (Illumina) at Genechem Biopharmaceutical Technology Co, Ltd. DEGs were screened using a fold change threshold of |log_2_FC| ≥ 1, with statistical significance determined by a parametric F-test (*P* < .05) using the R package edgeR.

### Statistical Analysis

Statistical analyses were conducted using GraphPad Prism version 8.0. Data are presented as means ± standard deviation. Intergroup comparisons were carried out using the 2-tailed Student *t* test, one-way/two-way analysis of variance, or Wilcoxon rank sum test, as appropriate. A *P* value less than .05 was regarded as statistically significant, and significance levels are denoted as follows: ∗*P* < .05; ∗∗*P* < .01; ∗∗∗*P* < .001; ns, not significant.

## Supplementary Material

Supplementary Material

Extended PDF
