# Supplementary material for: IGF2BP1-Mediated m⁶A Modification Stabilizes HMGA2 mRNA to Promote Intrahepatic Cholangiocarcinoma Progression
Source: Cell Mol Gastroenterol Hepatol. 2026 Jun 24;20(10):101837. doi: 10.1016/j.jcmgh.2026.101837 (PMC13474244; doi:10.1016/j.jcmgh.2026.101837)

Figure 1. E

HMGA2

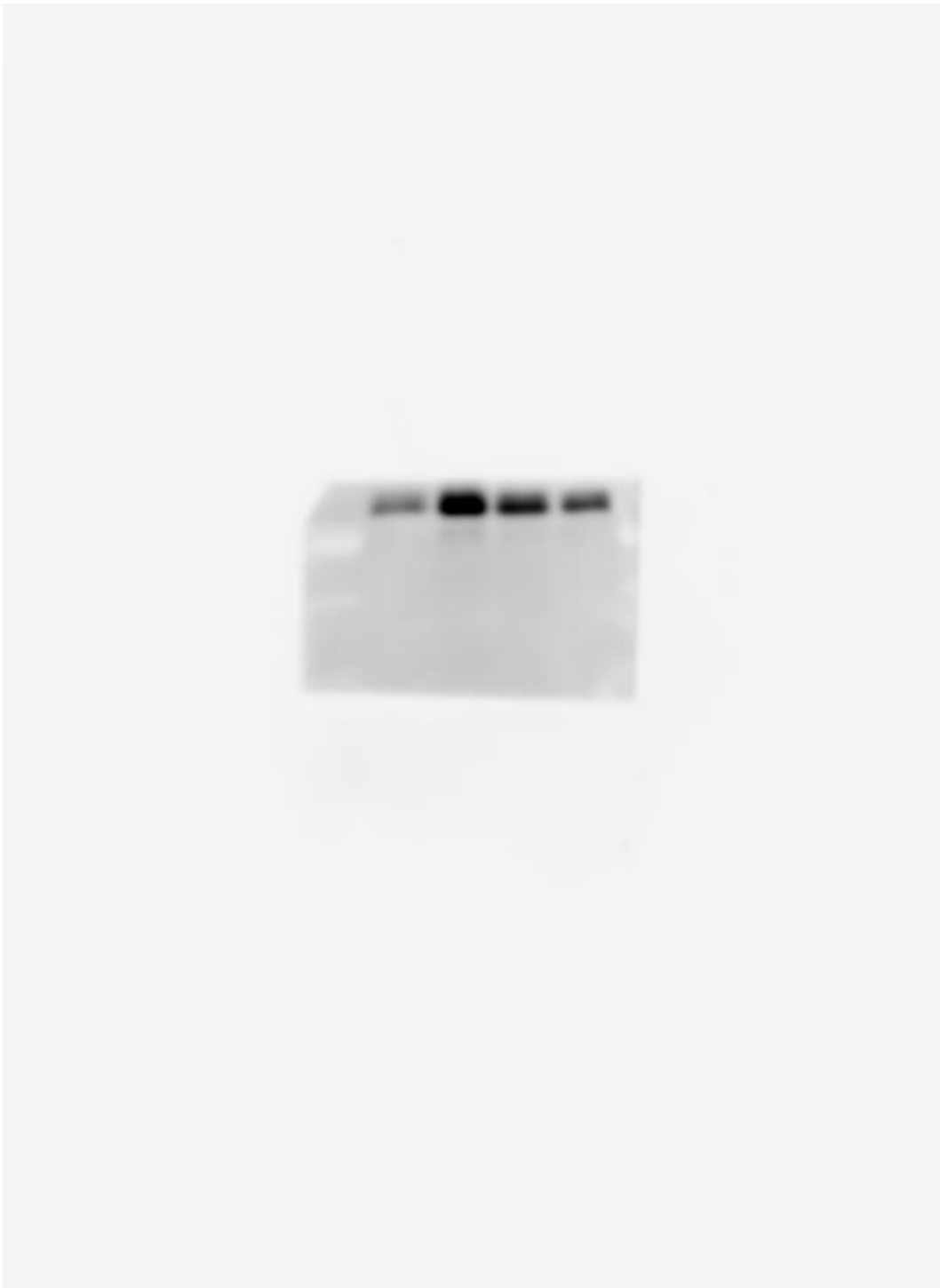

β-actin

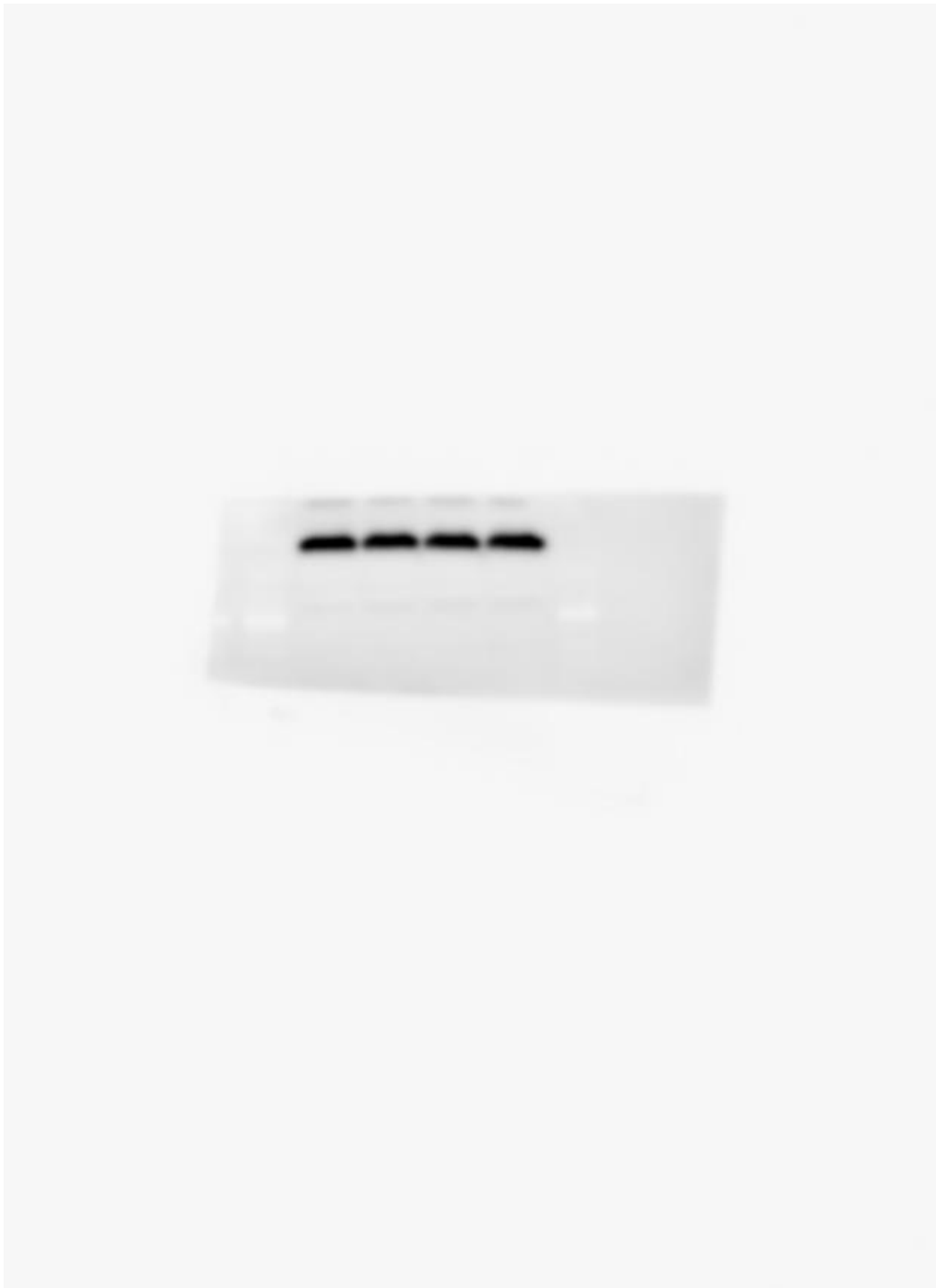

Figure 1. F

HMGA2

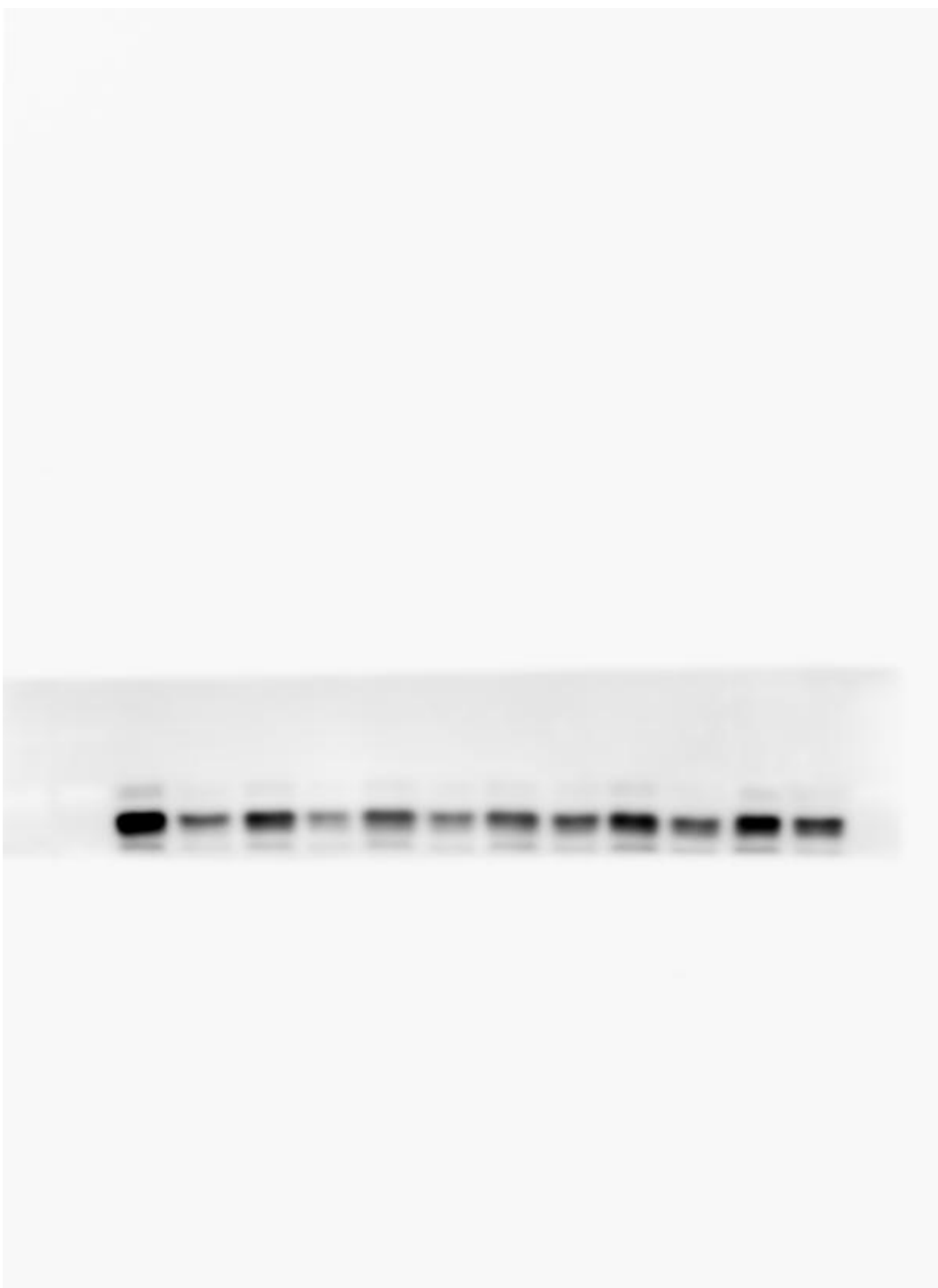

β-actin

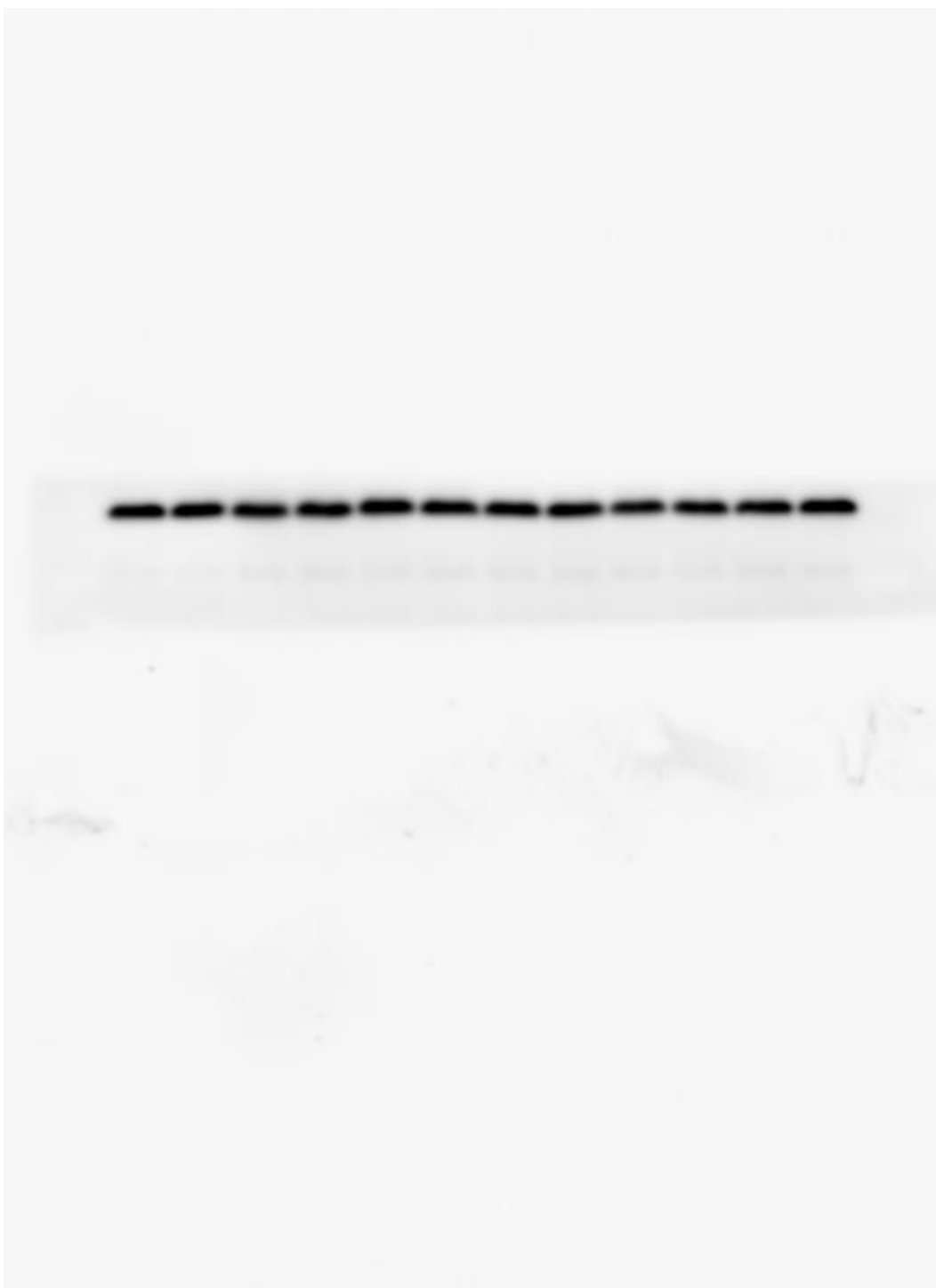

Figure 3. A

CCLP1-HMGA2

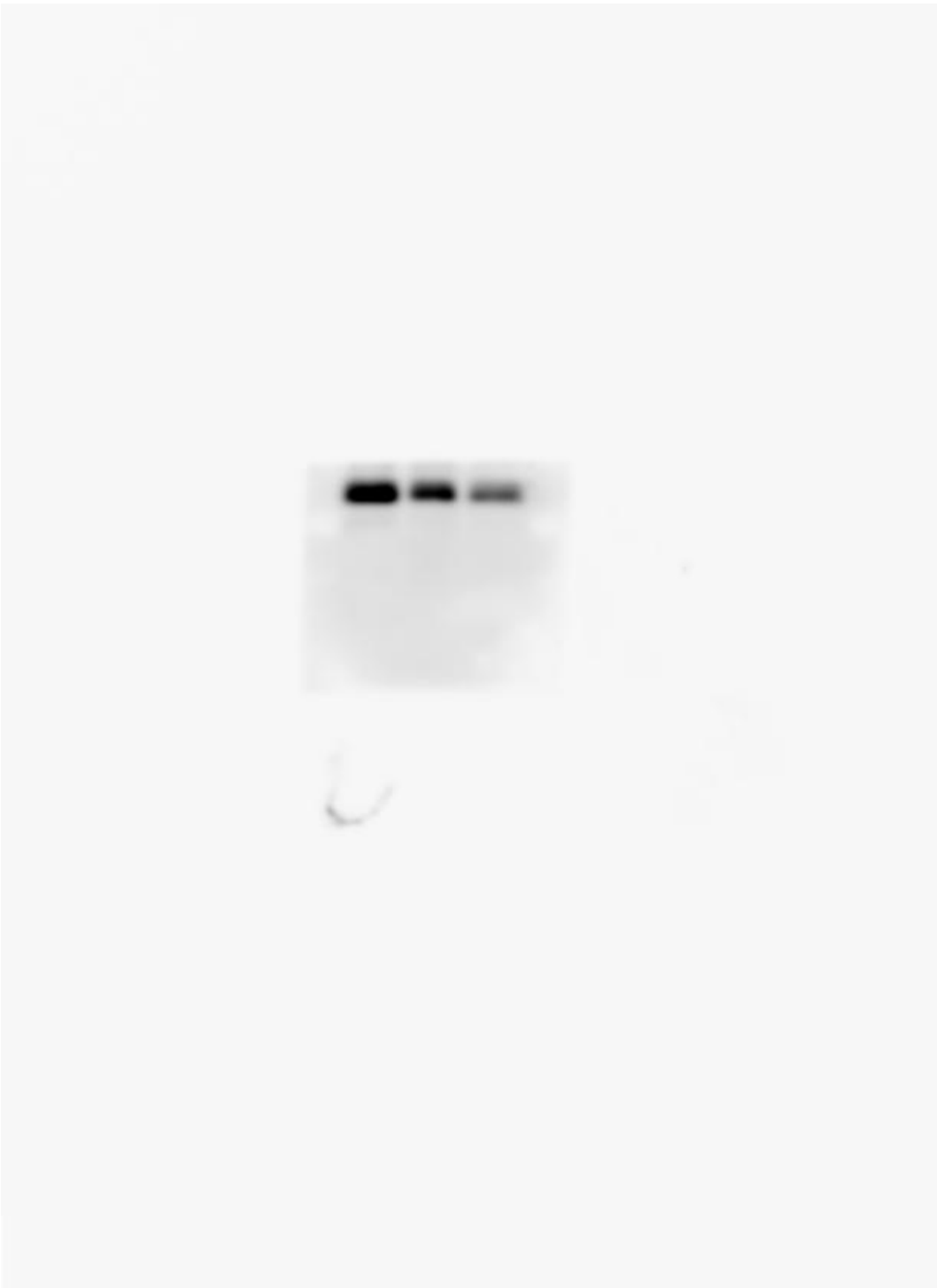

CCLP1-β-actin

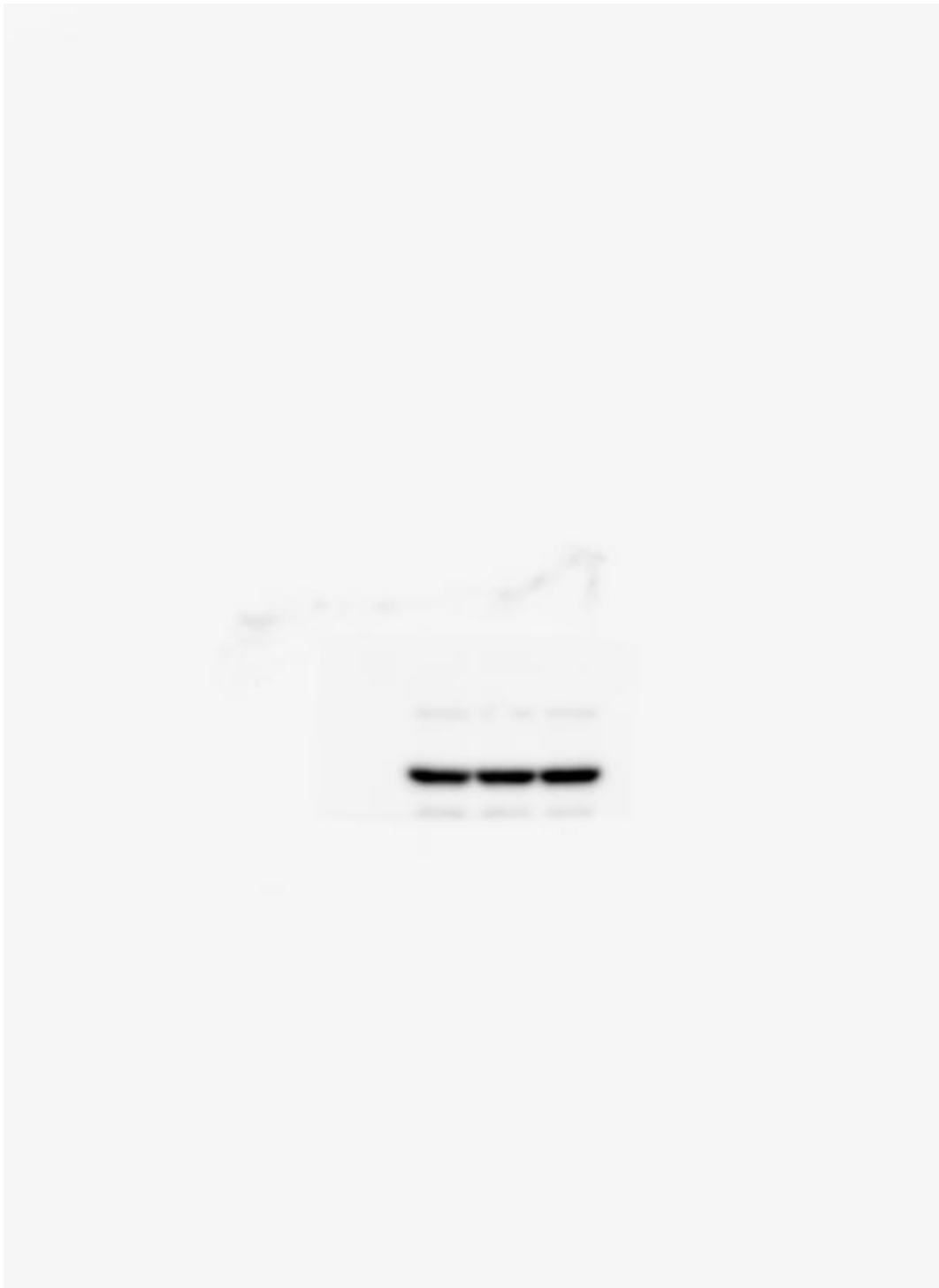

HuCCT1-HMGA2

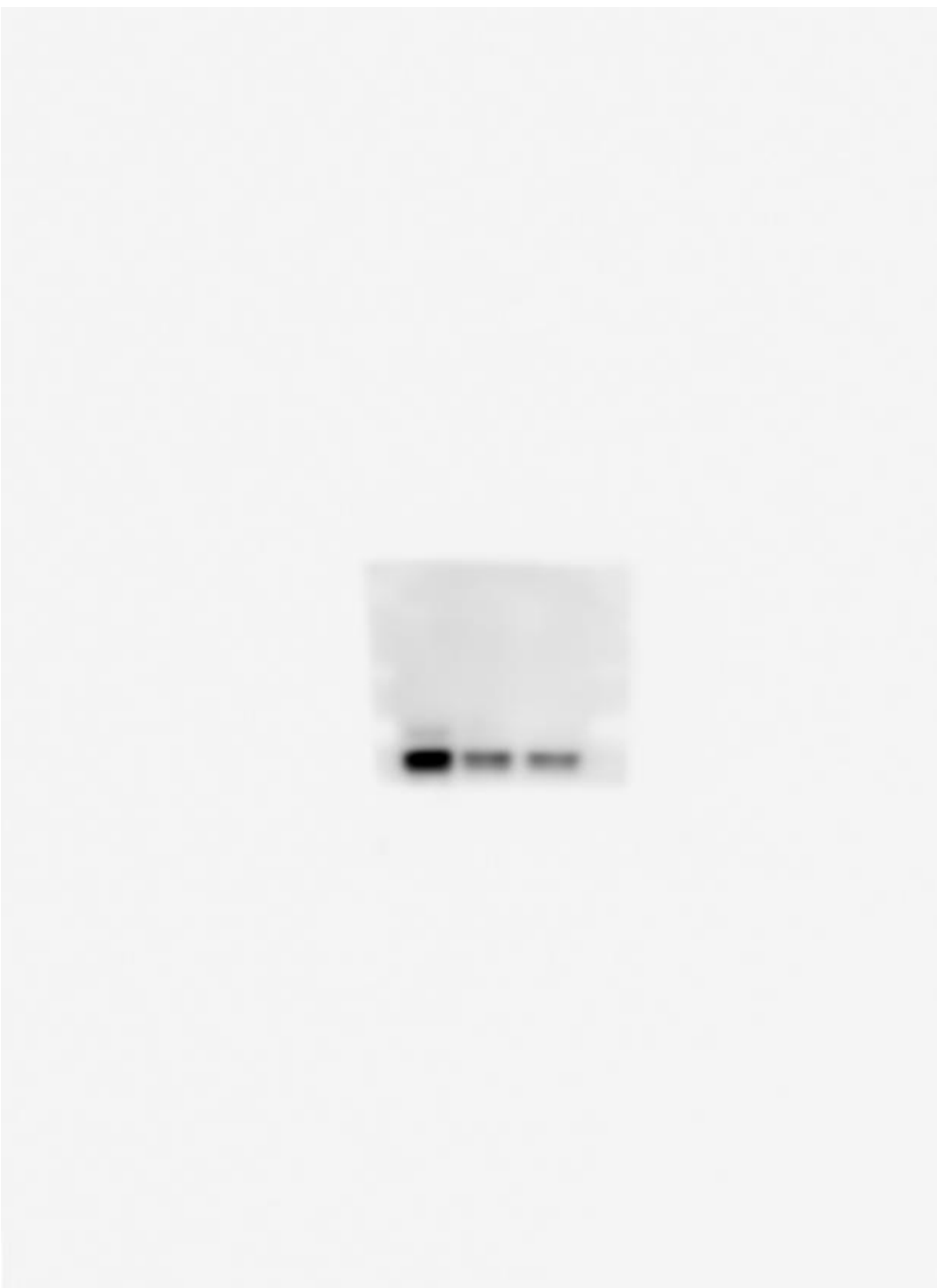

HuCCT1-β-actin

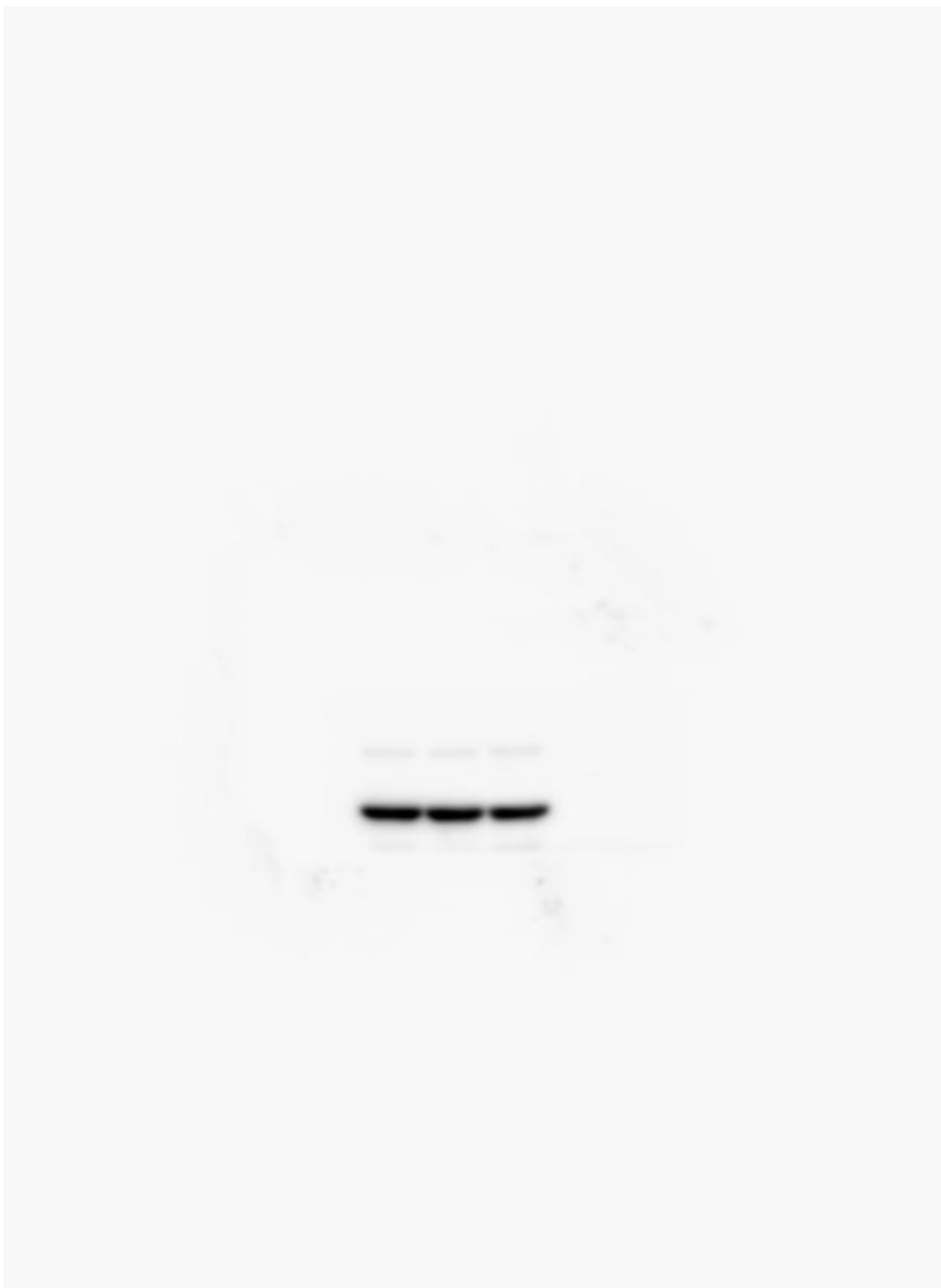

Figure 7. J

IGF2BP1

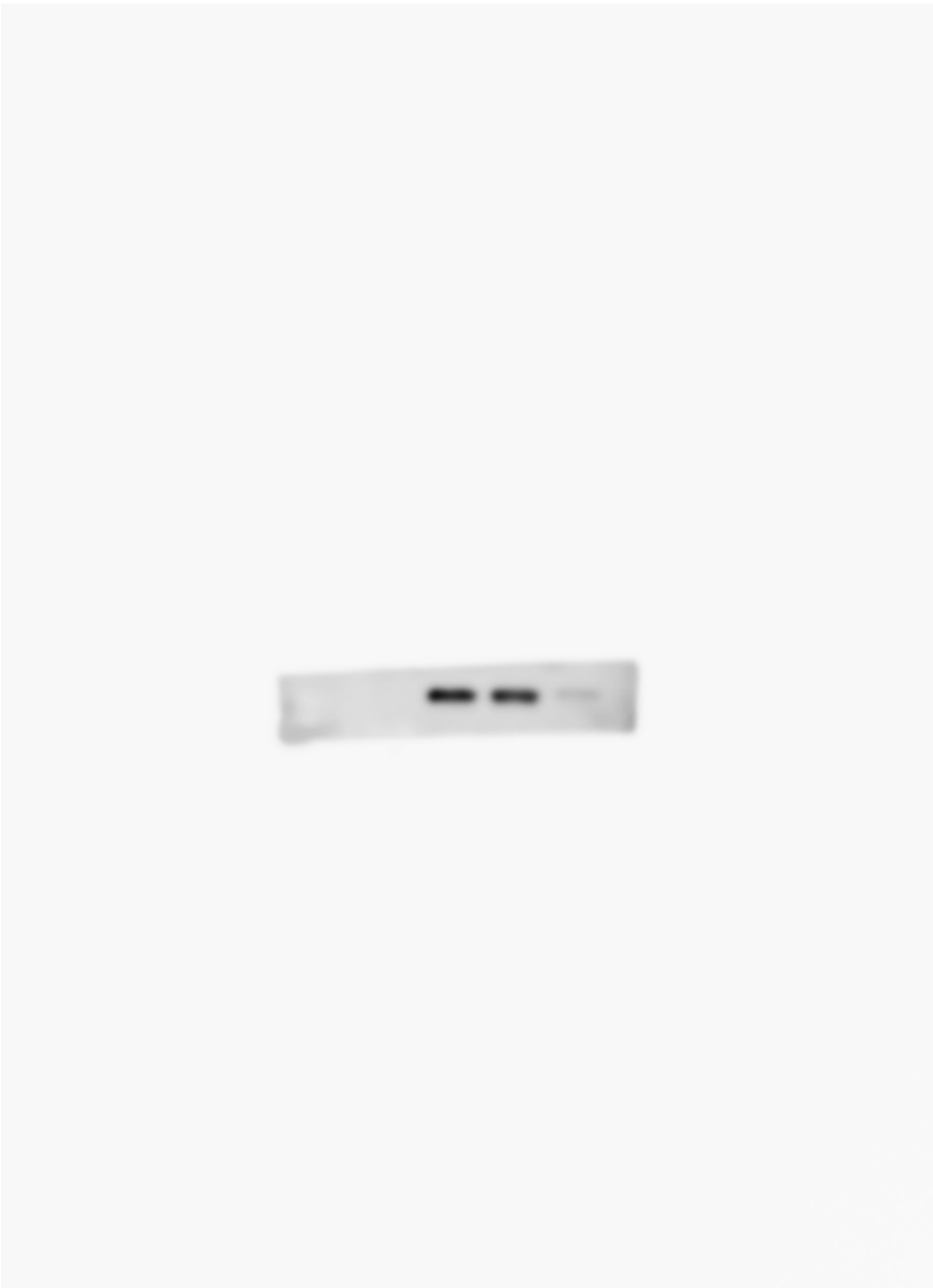

Figure 7. L

IGF2BP1

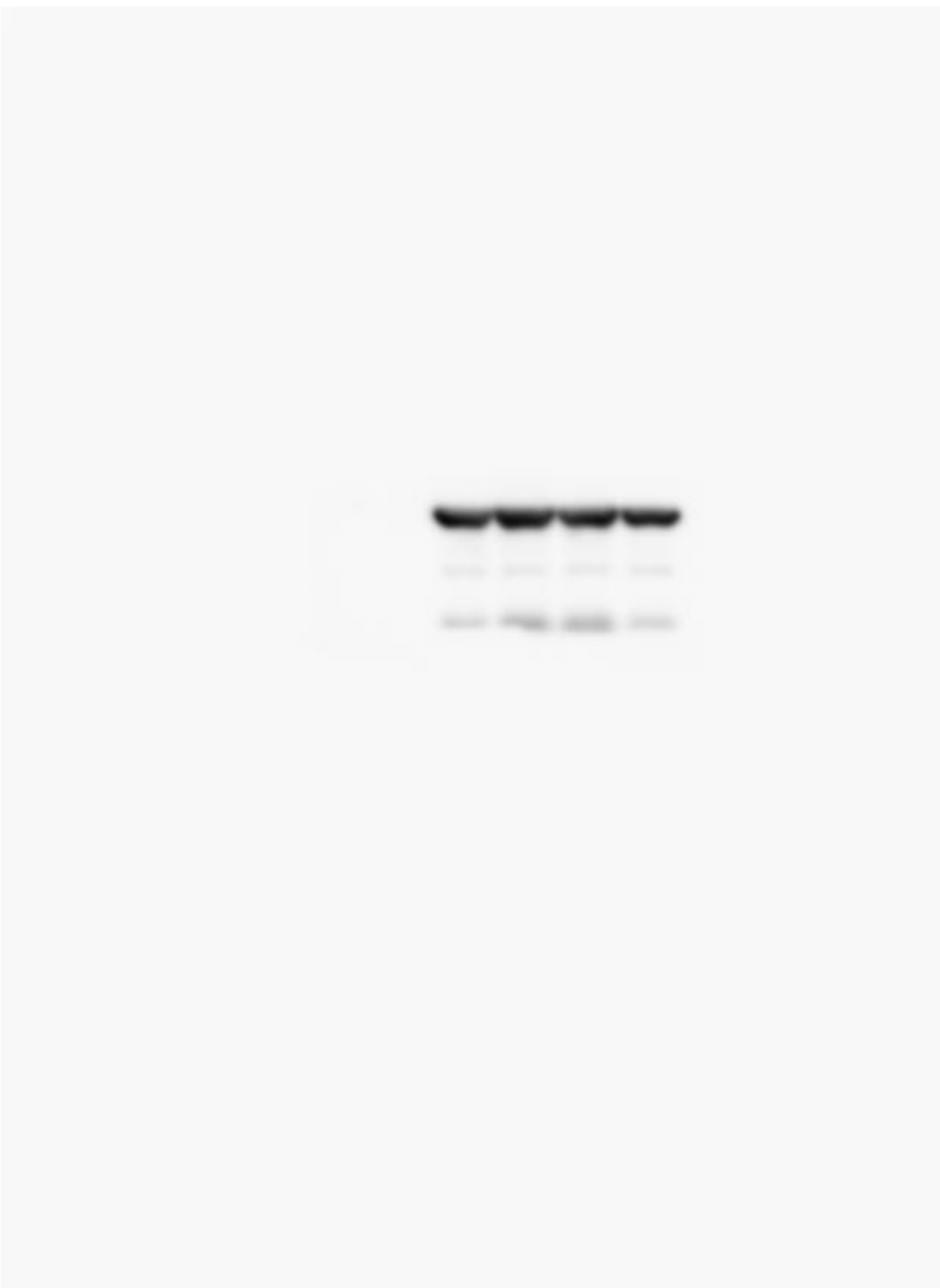

β-actin

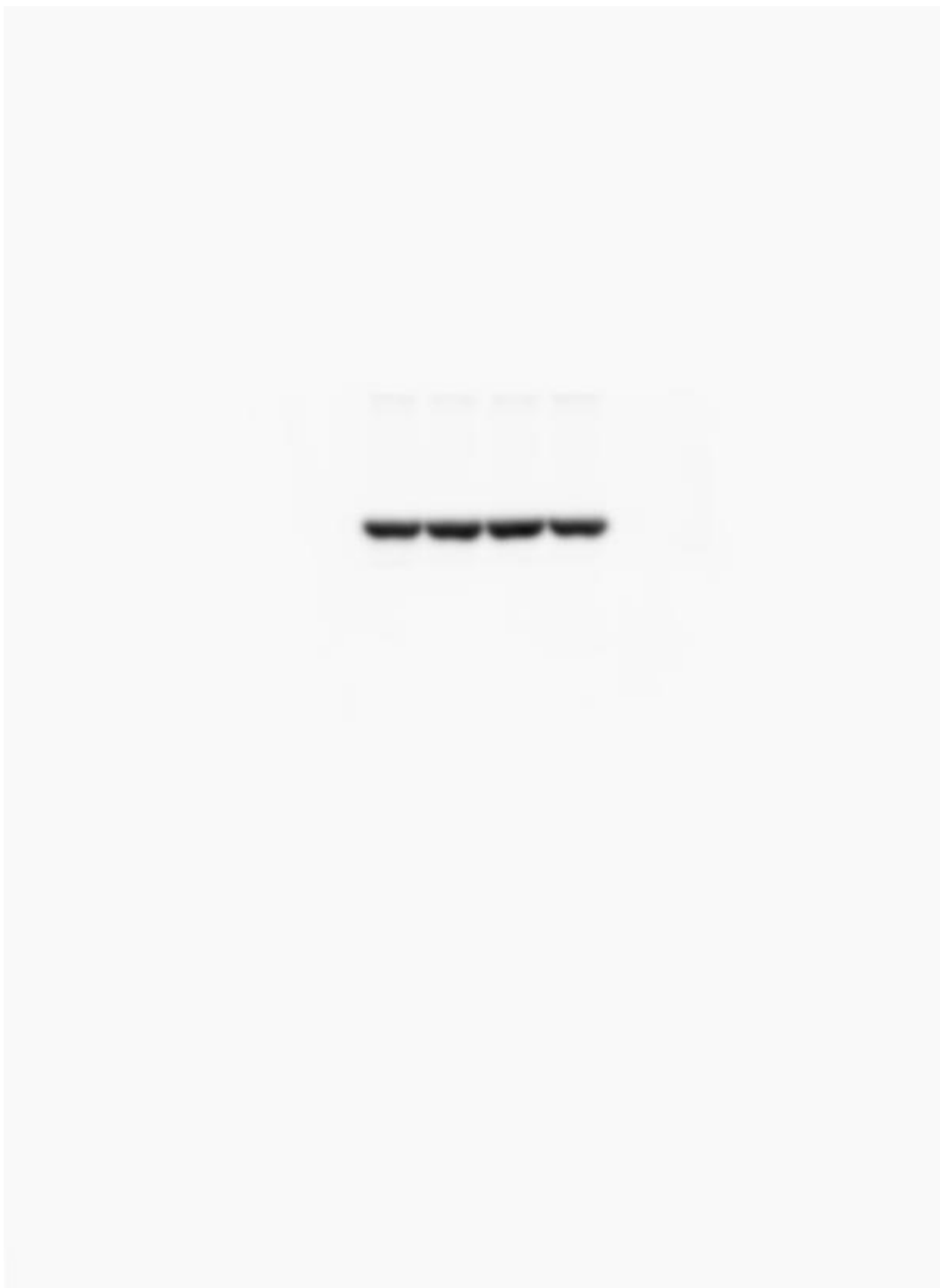

Figure 10. D

HMGA2-CCLP1

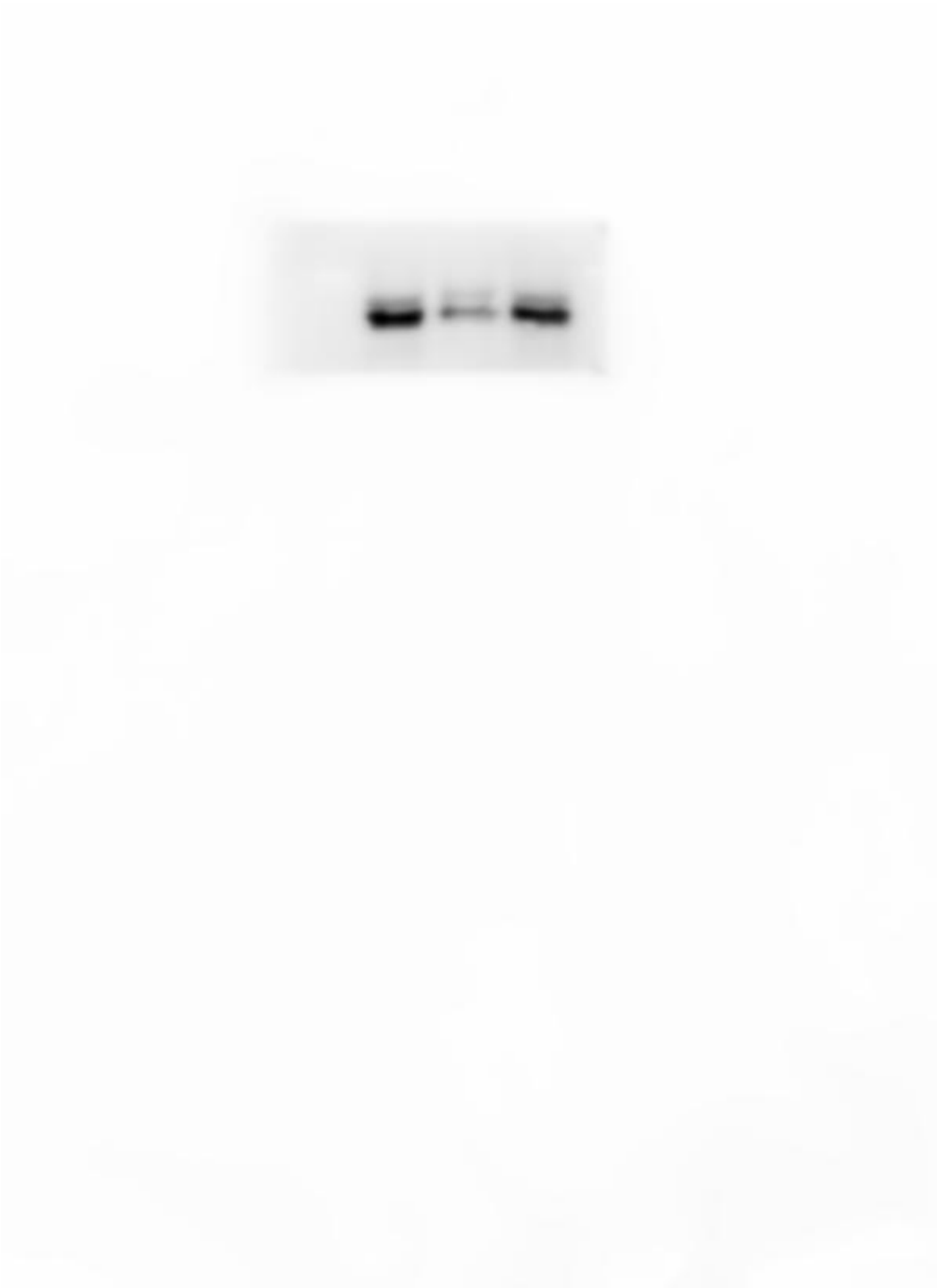

HMGA2-HuCCT1

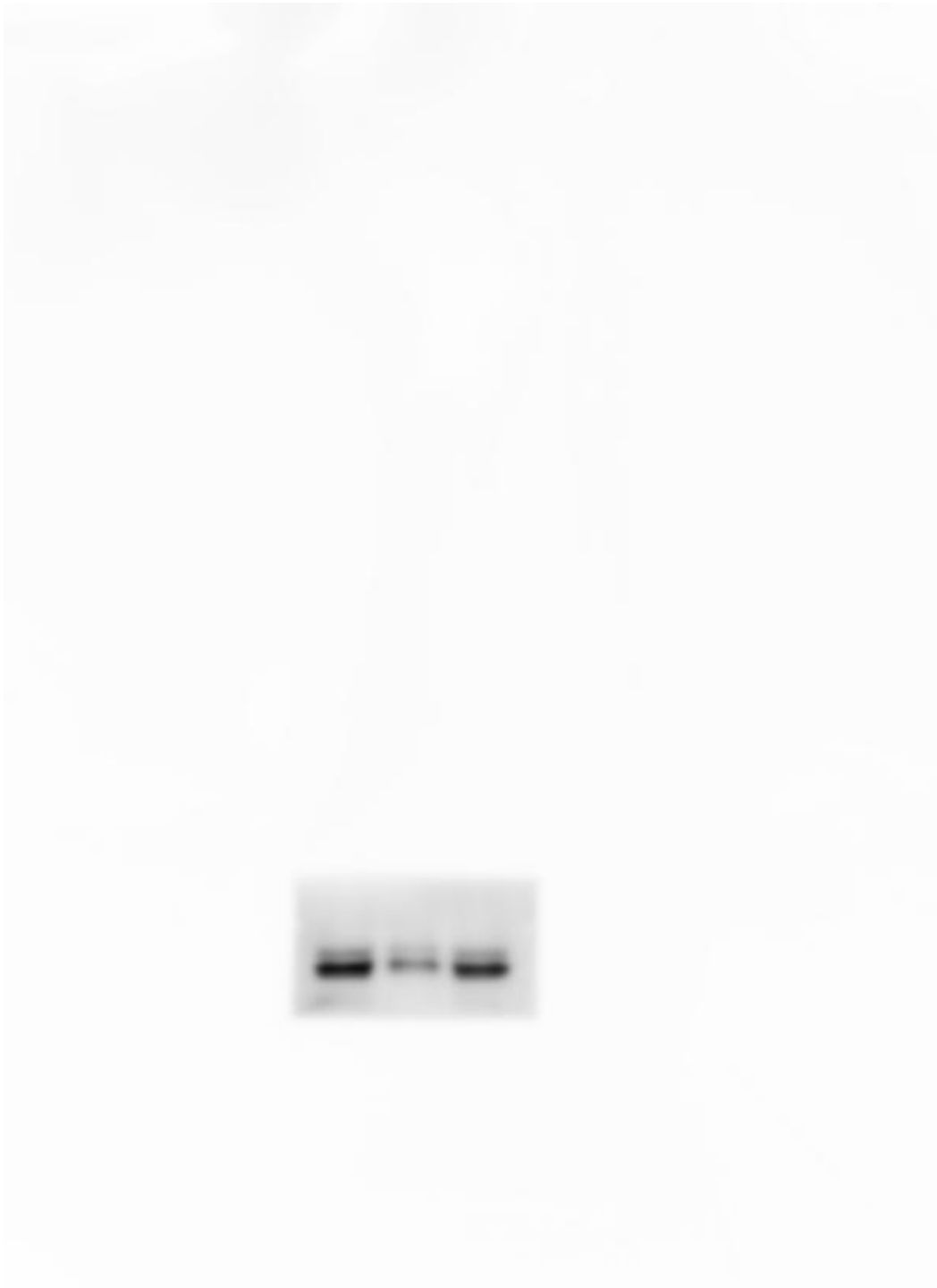

P-PI3K-CCLP1

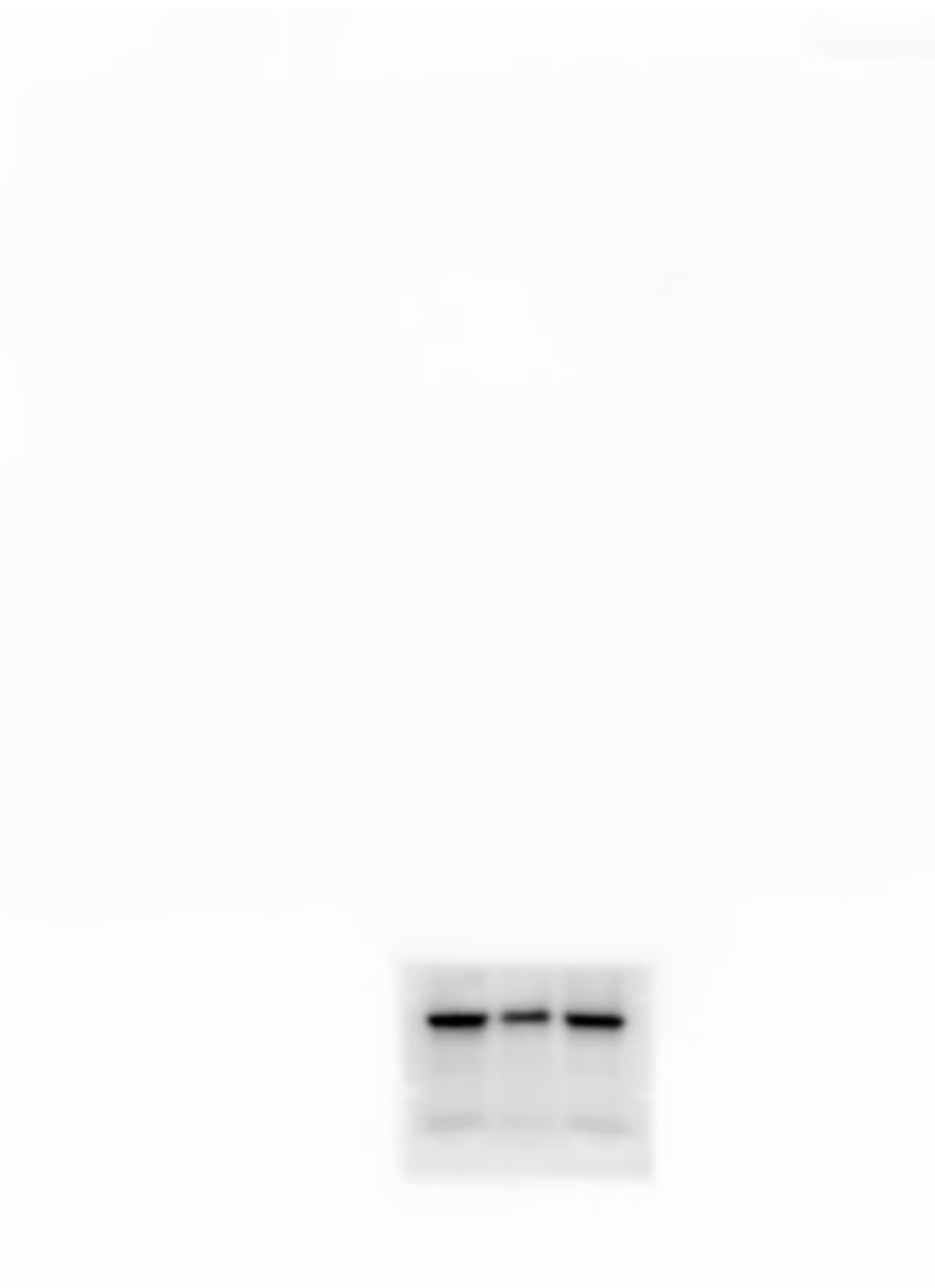

P-PI3K-HuCCT1

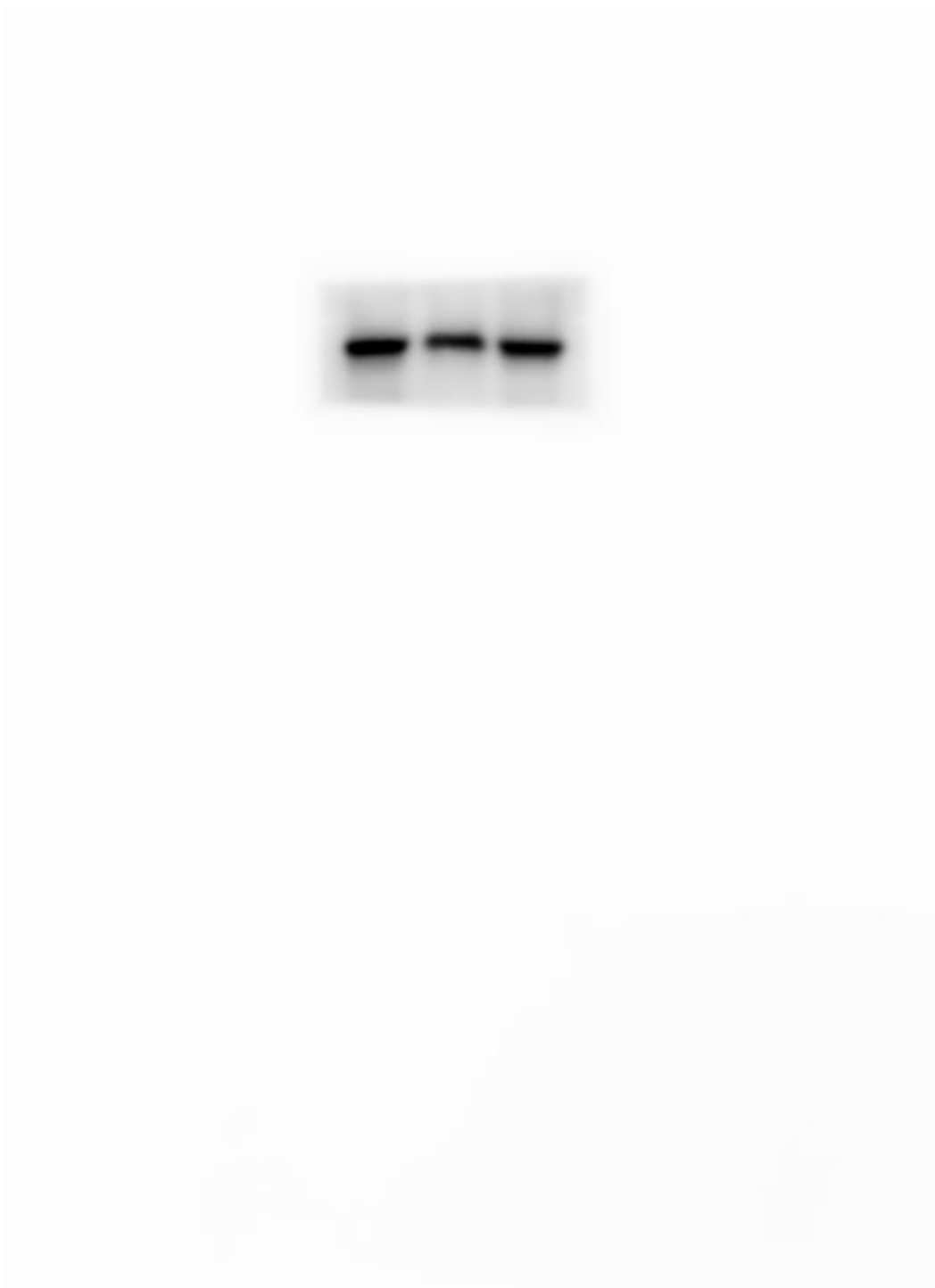

PI3K-CCLP1

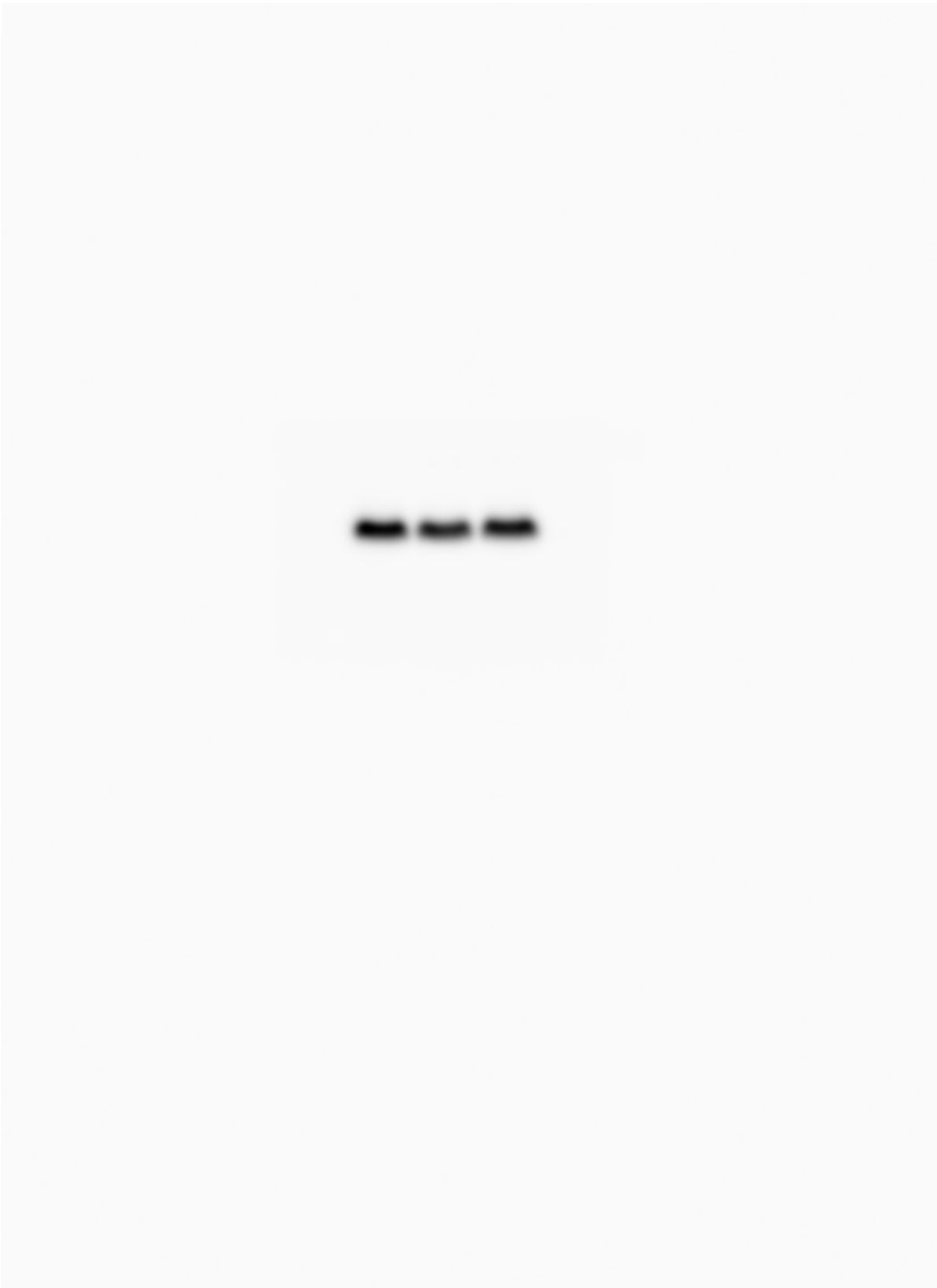

PI3K-HuCCT1

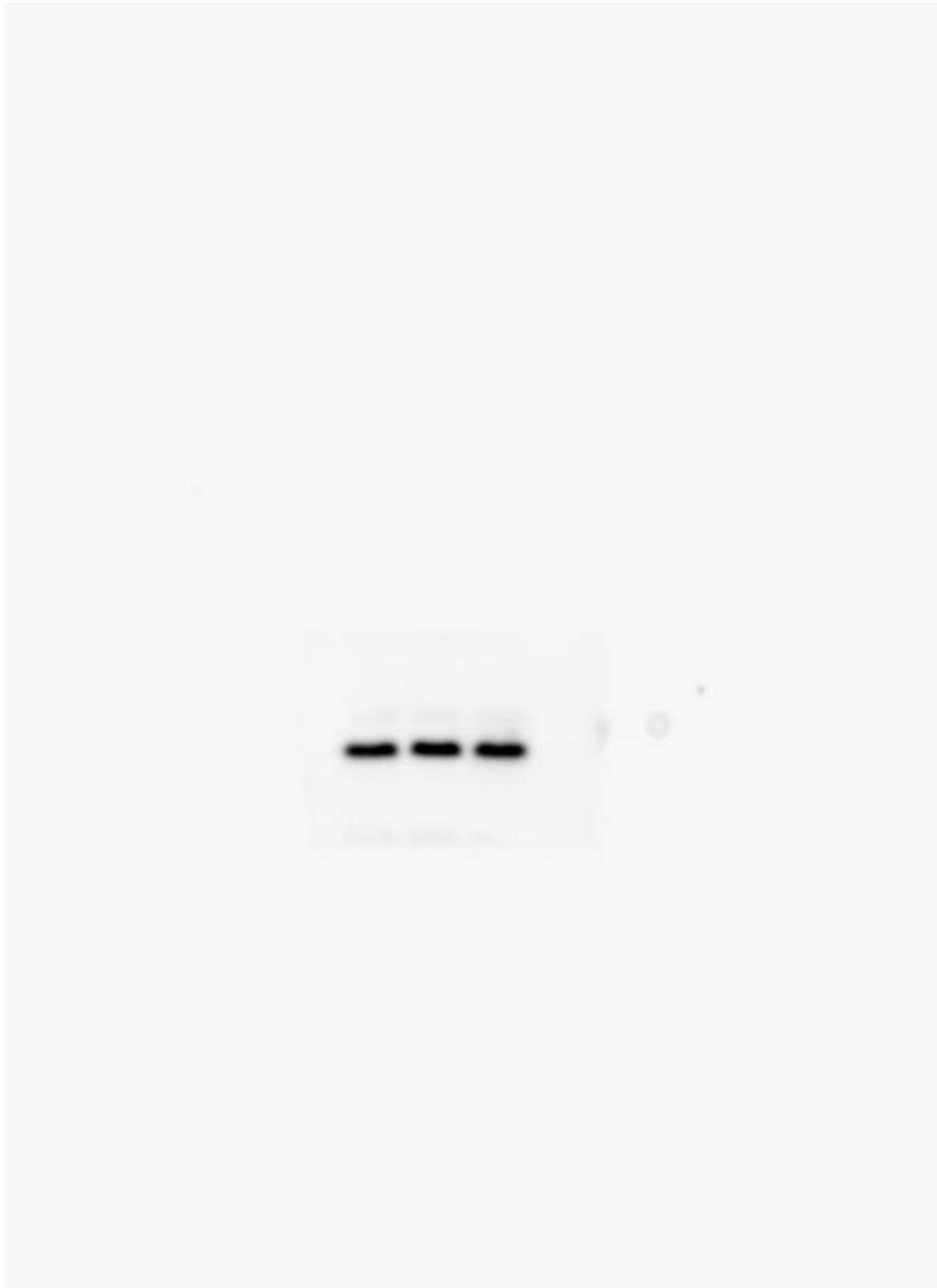

P-AKT-CCLP1

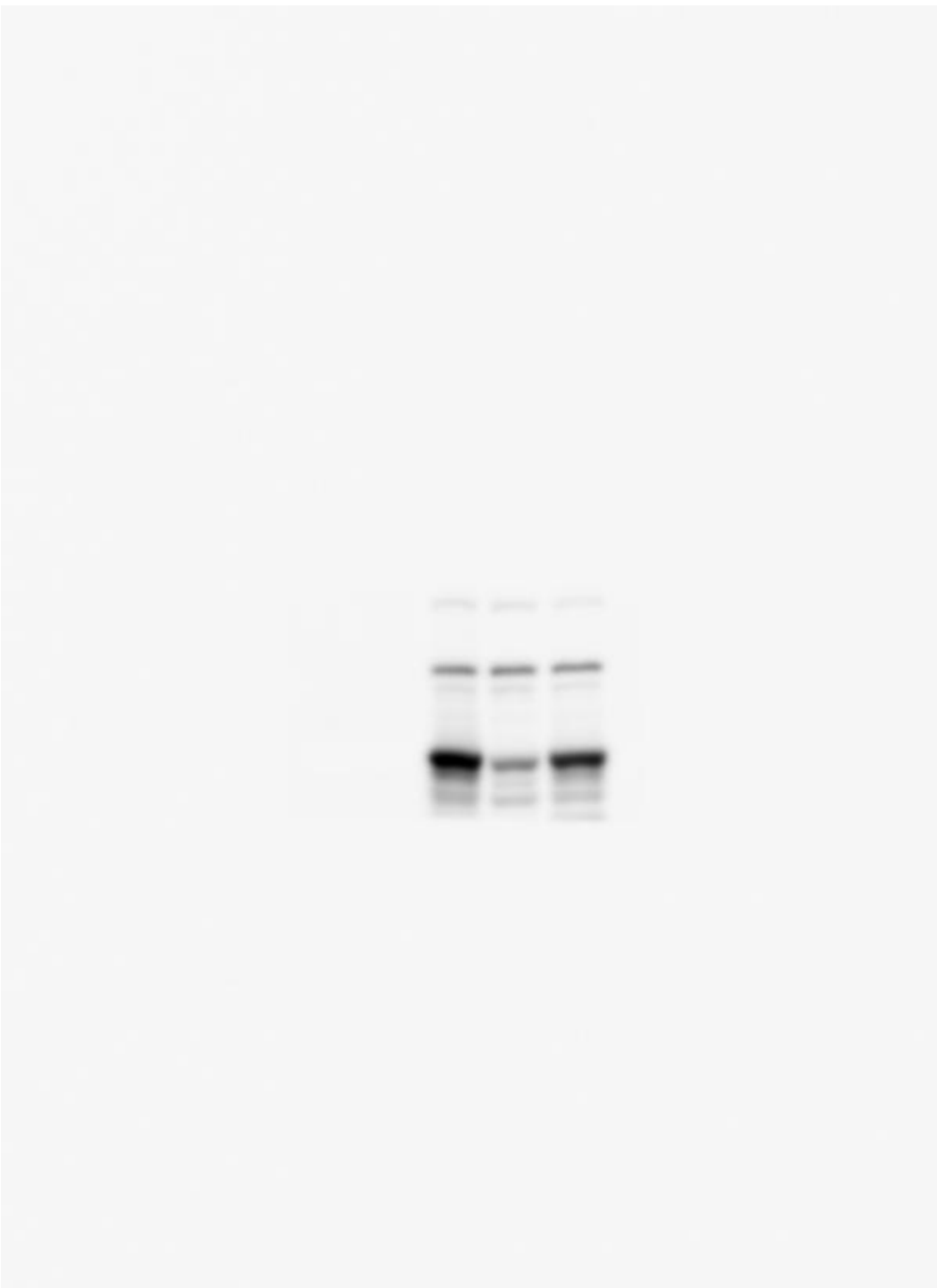

P-AKT-HuCCT1

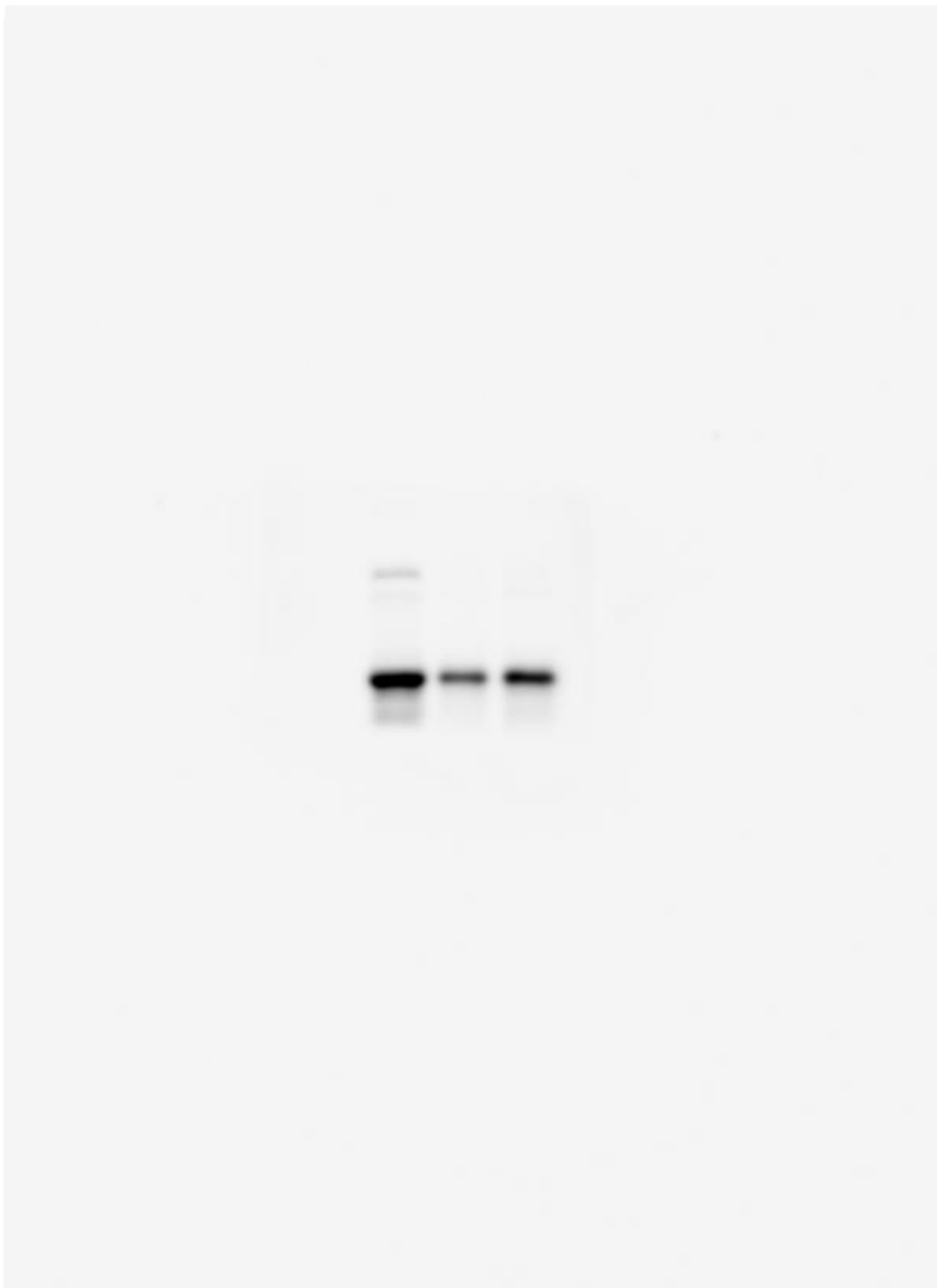

AKT-CCLP1

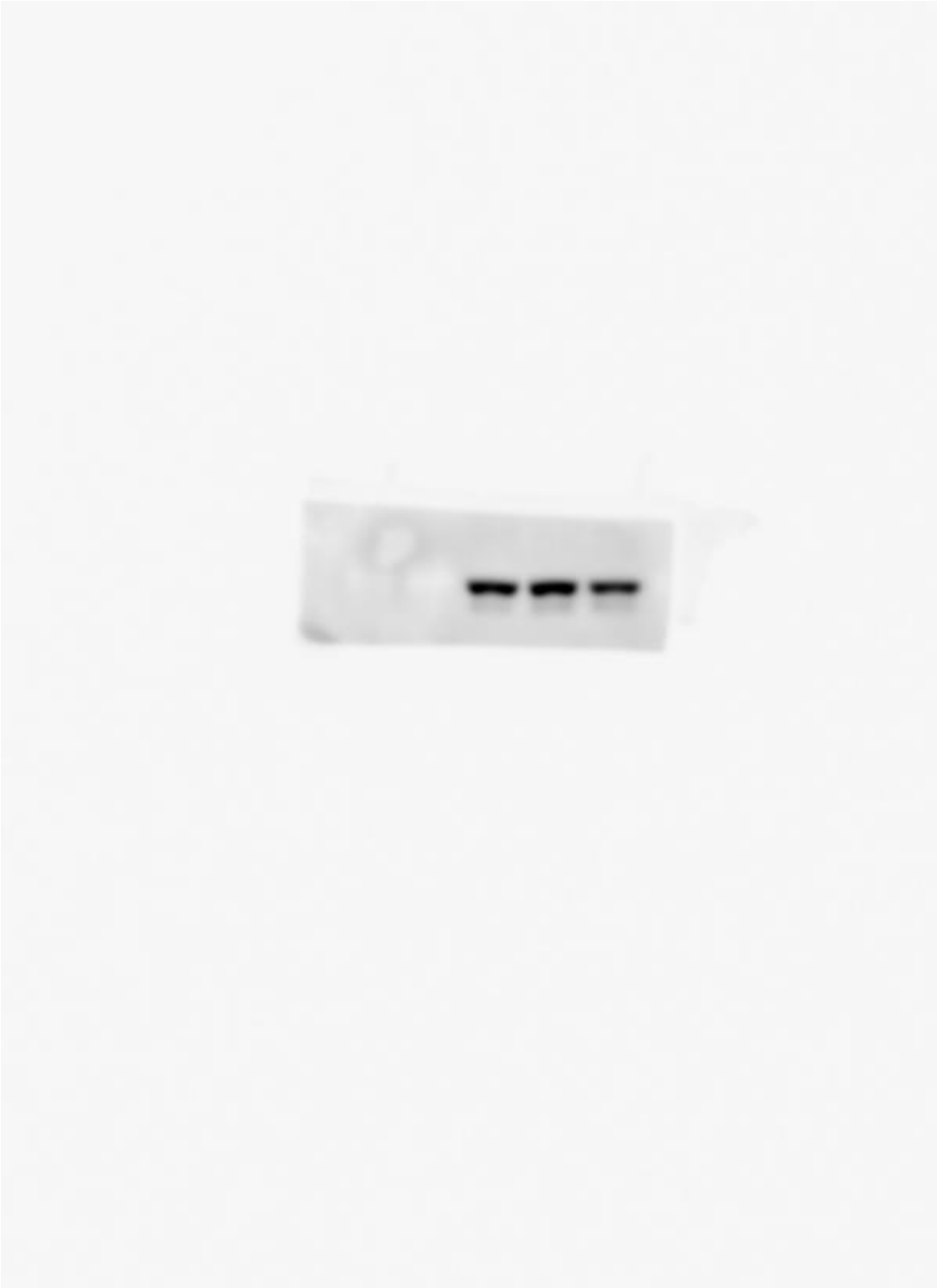

AKT-HuCCT1

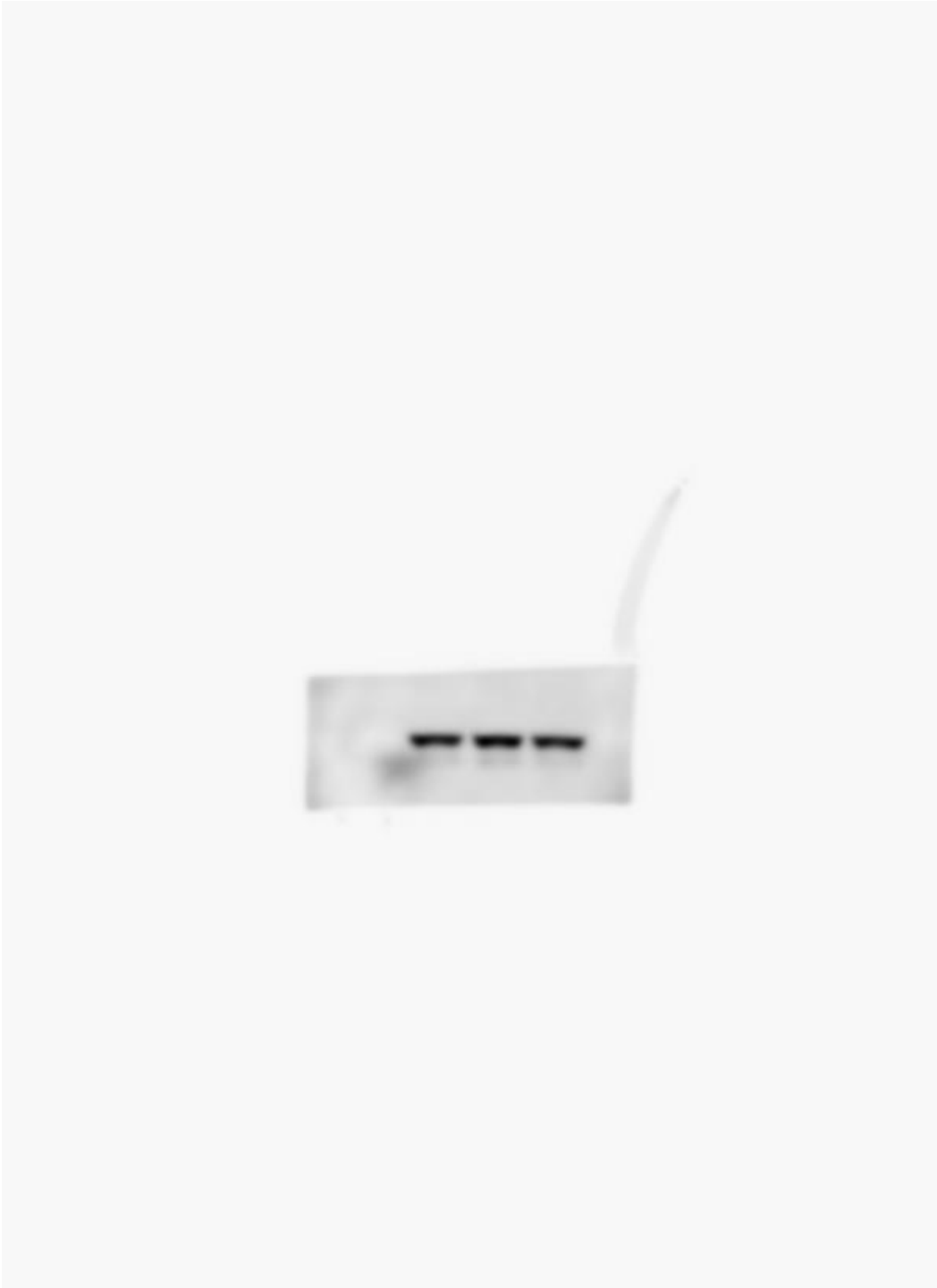

GAPDH-CCLP1

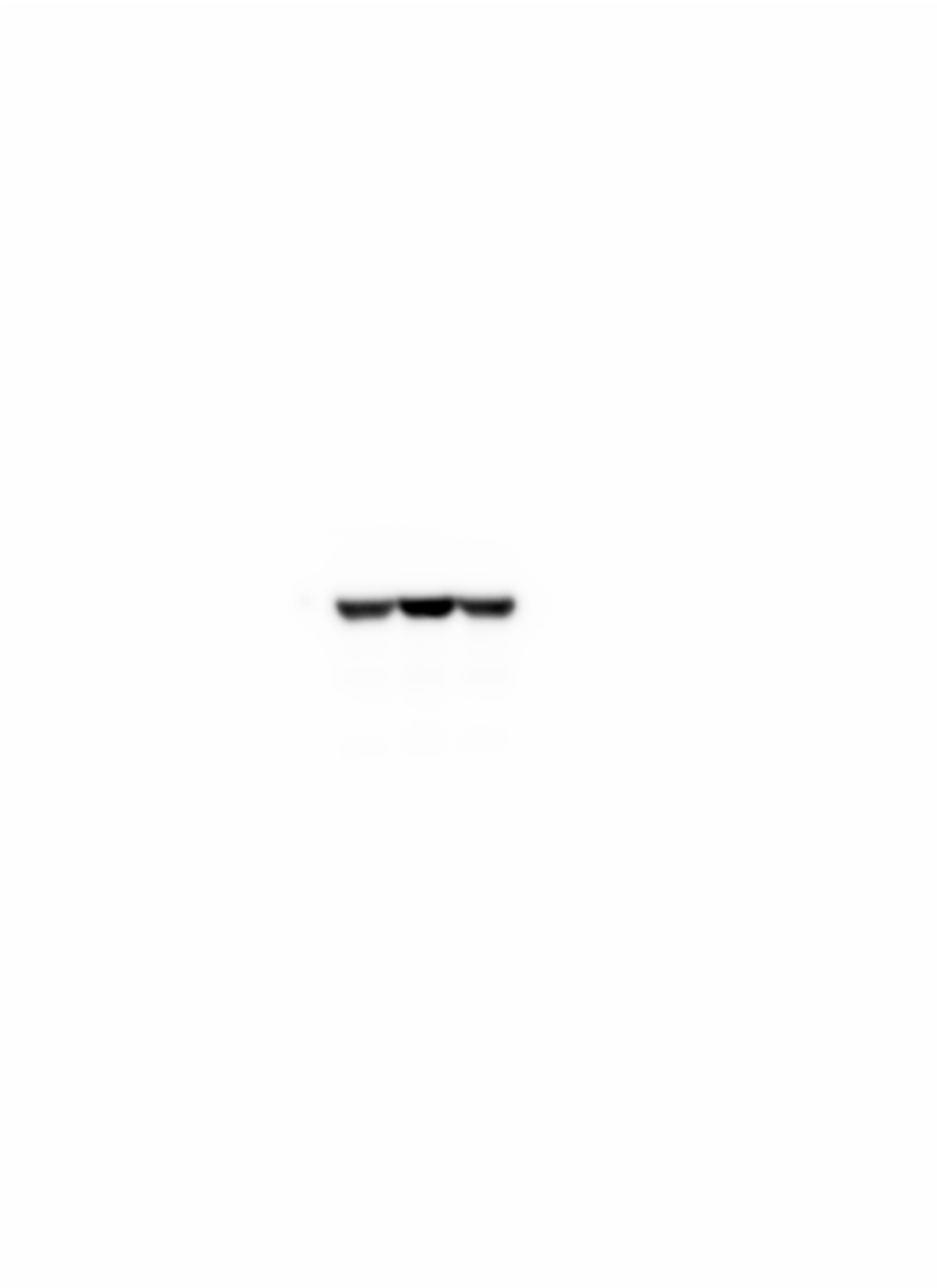

GAPDH-HuCCT1

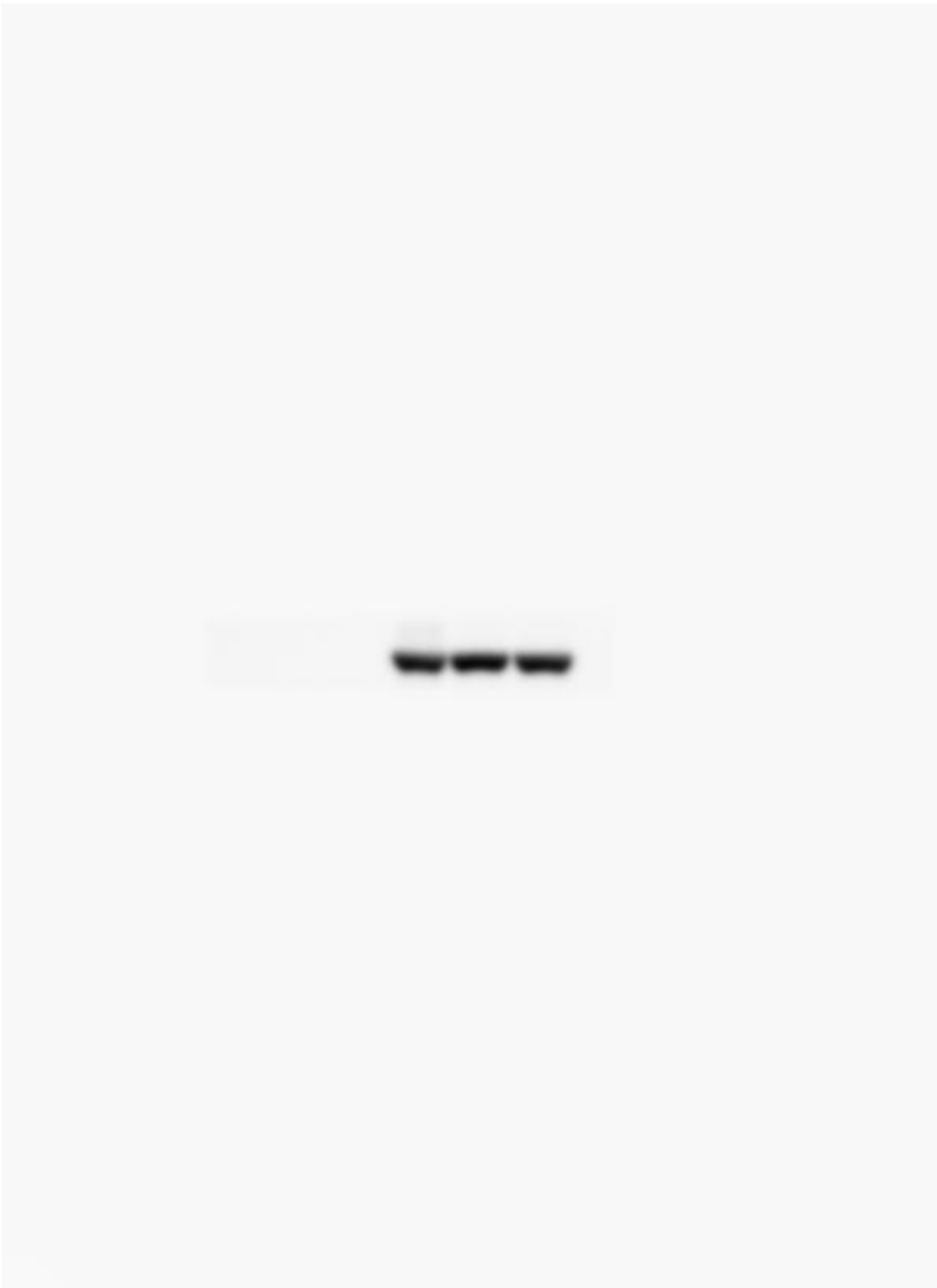

Figure S3. A

HMGA2-CCLP1

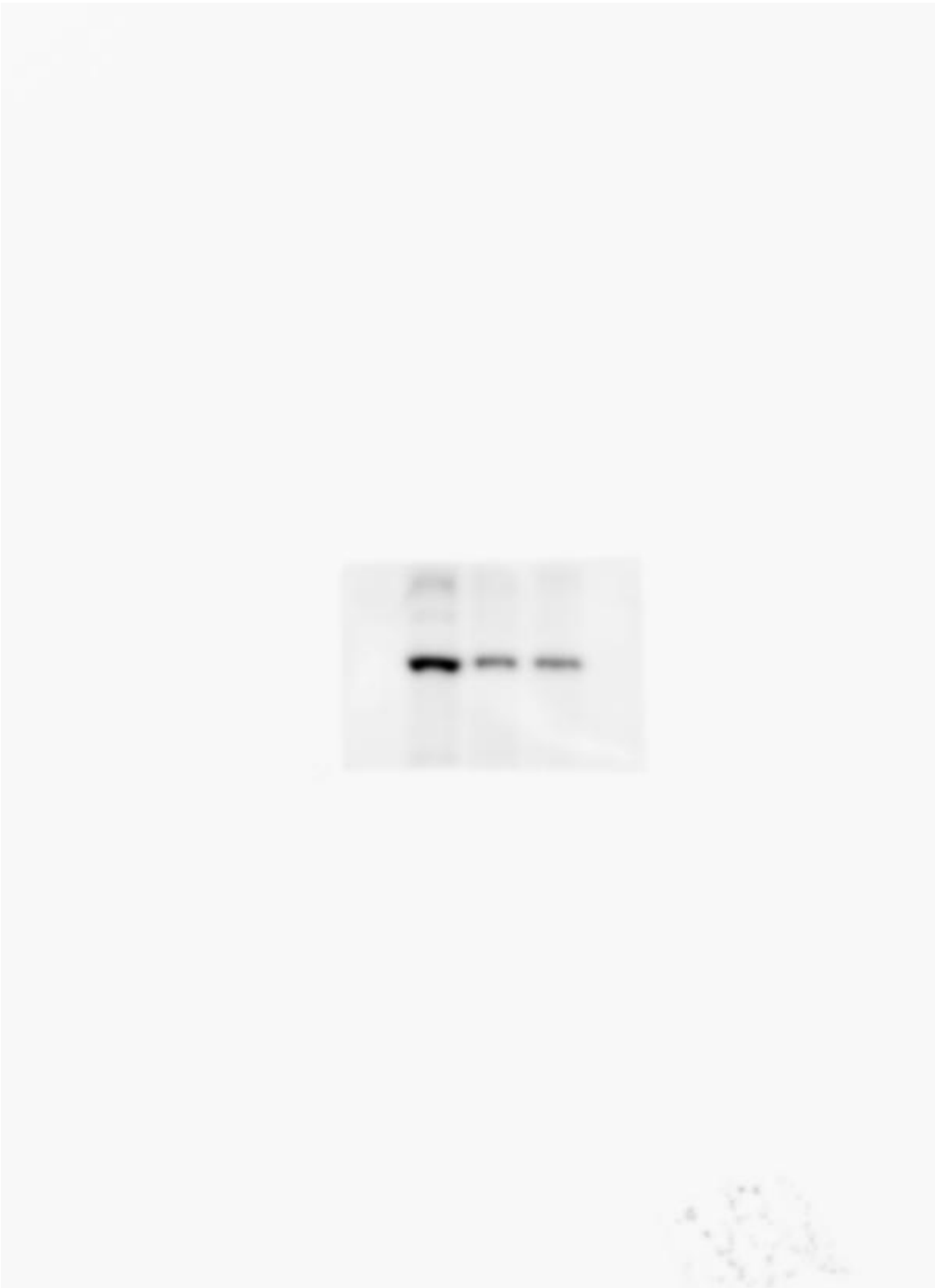

HMGA2-HuCCT1

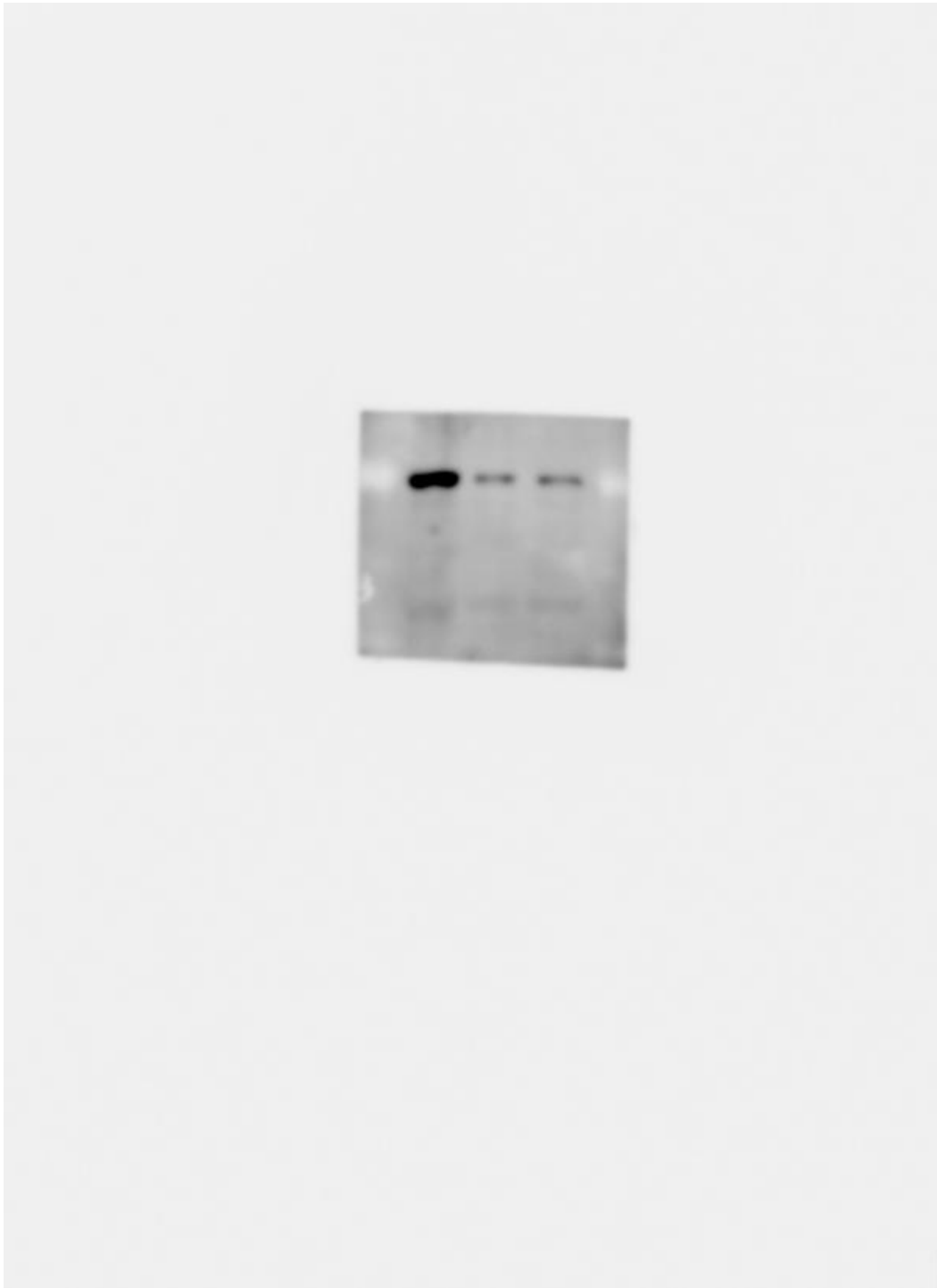

β-actin-CCLP1

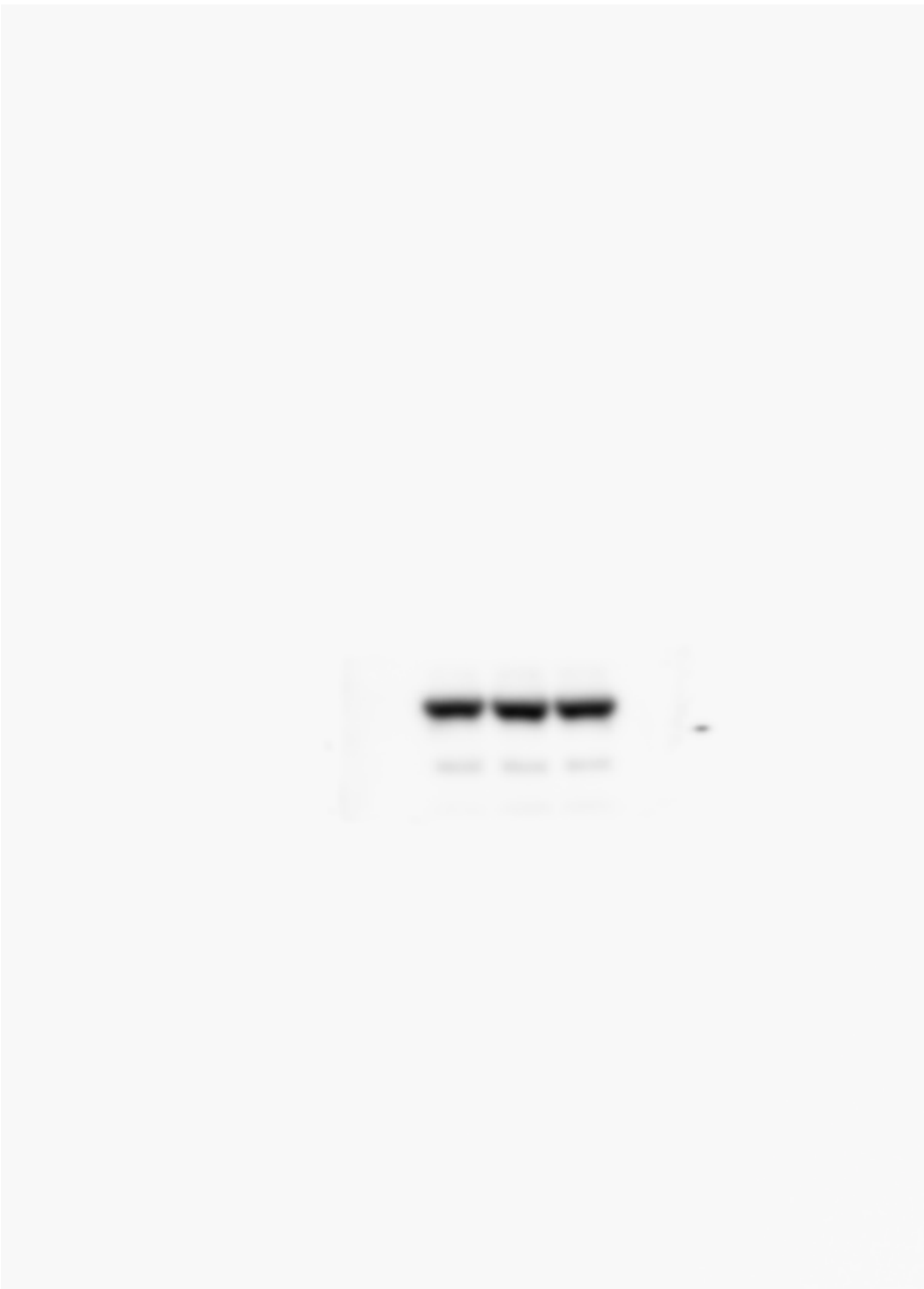

β-actin-HuCCT1

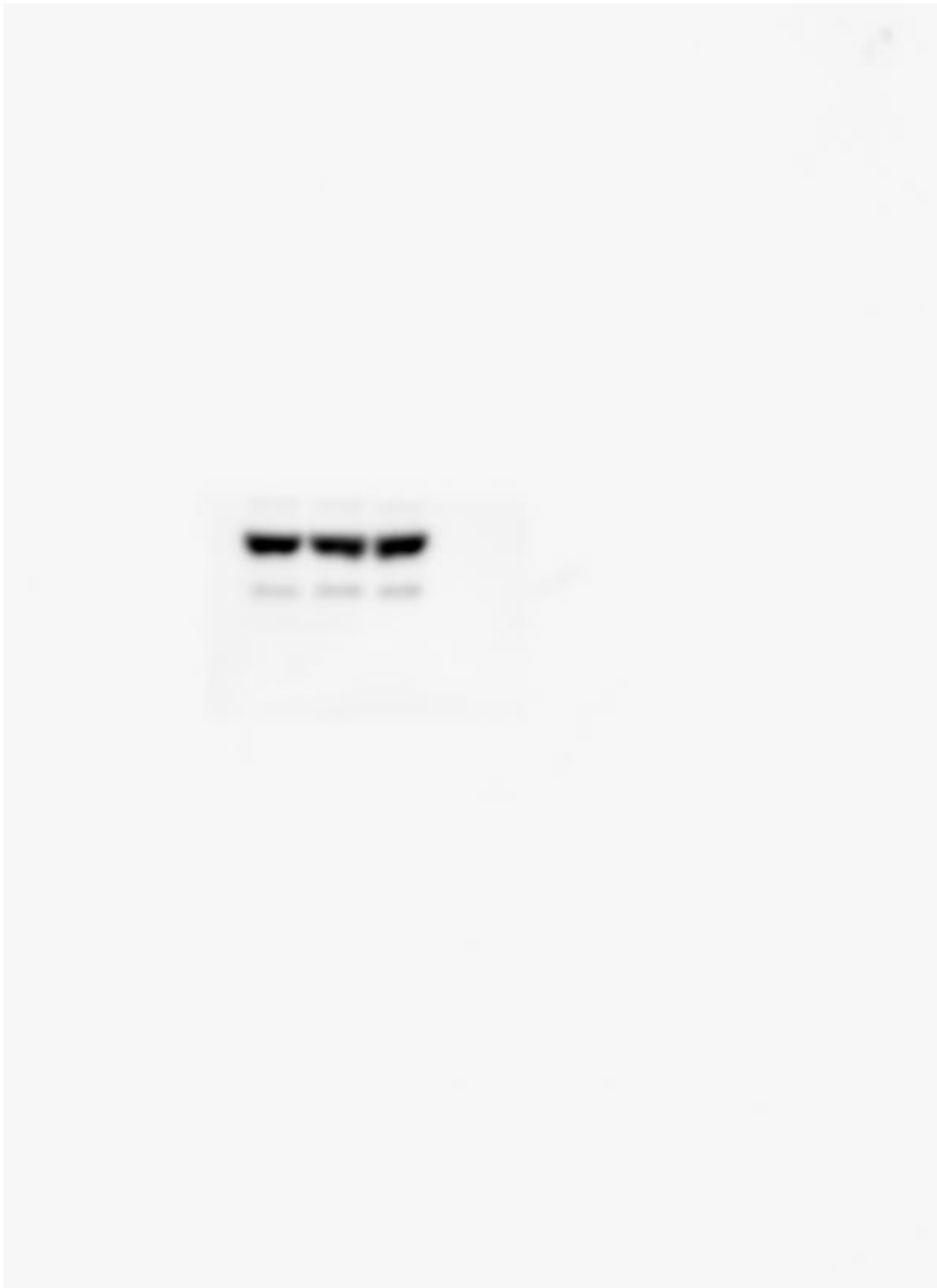

Figure S4. A

HMGA2

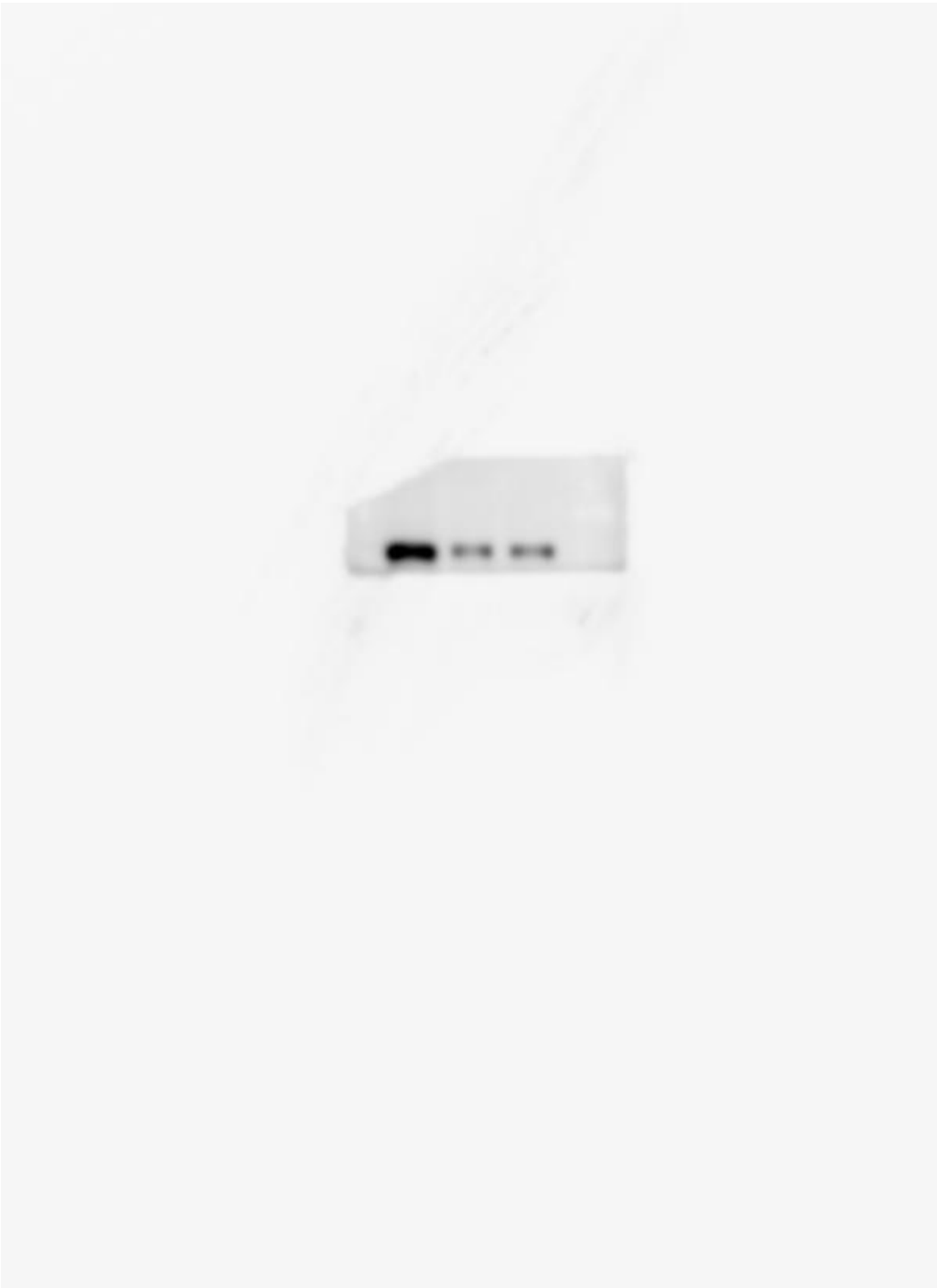

β-actin

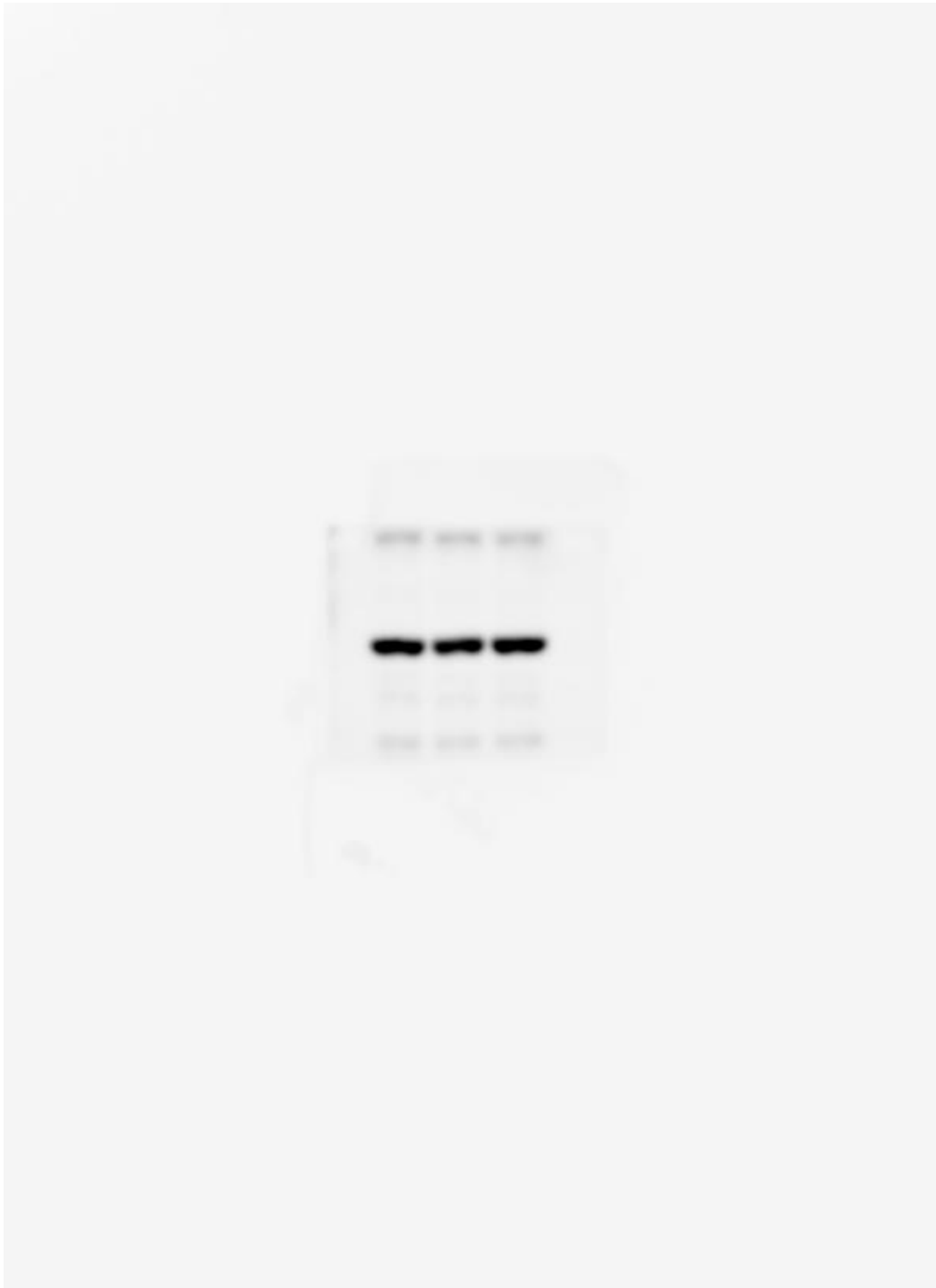

Figure S4. B

HMGA2

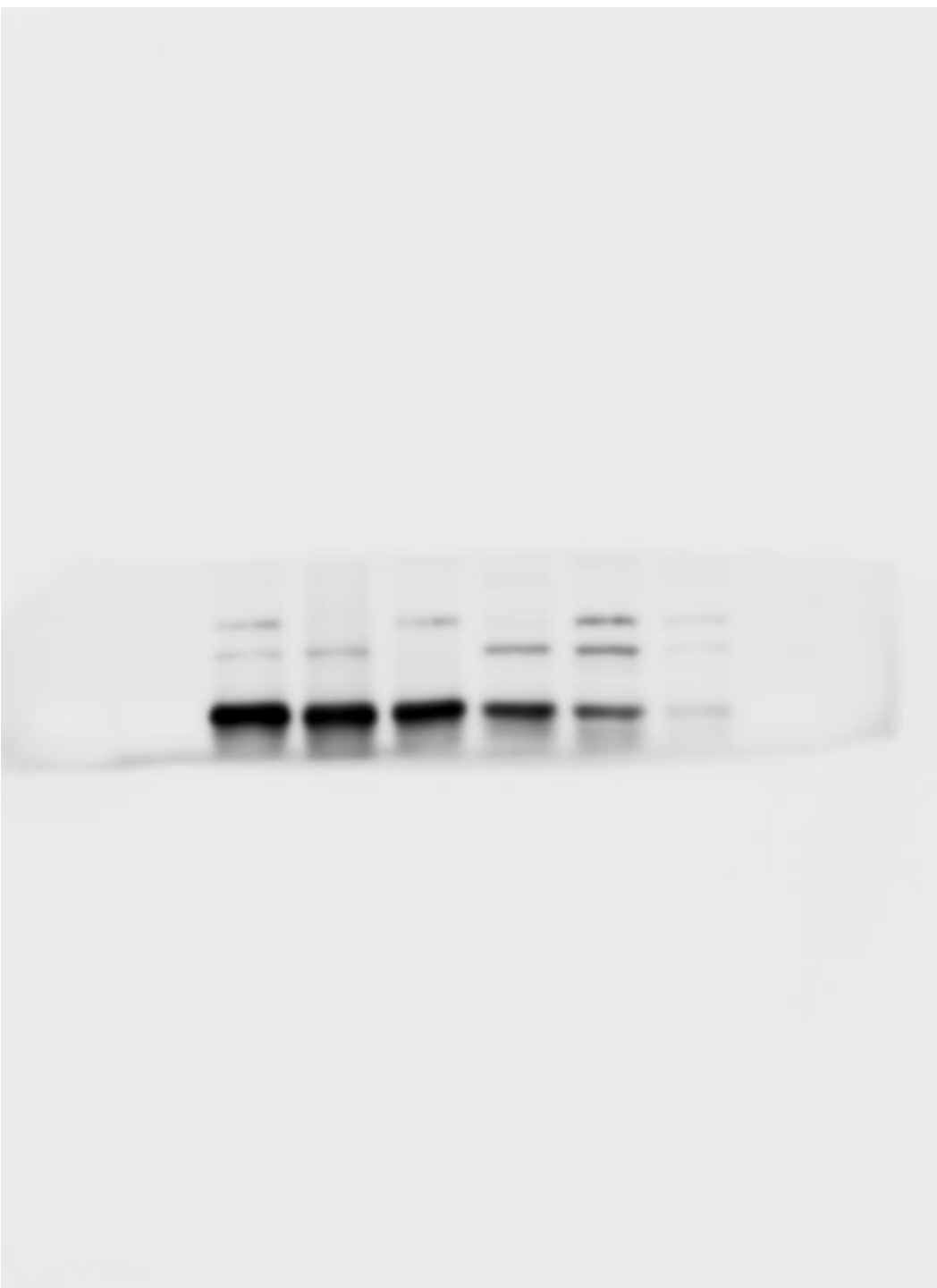

β-actin

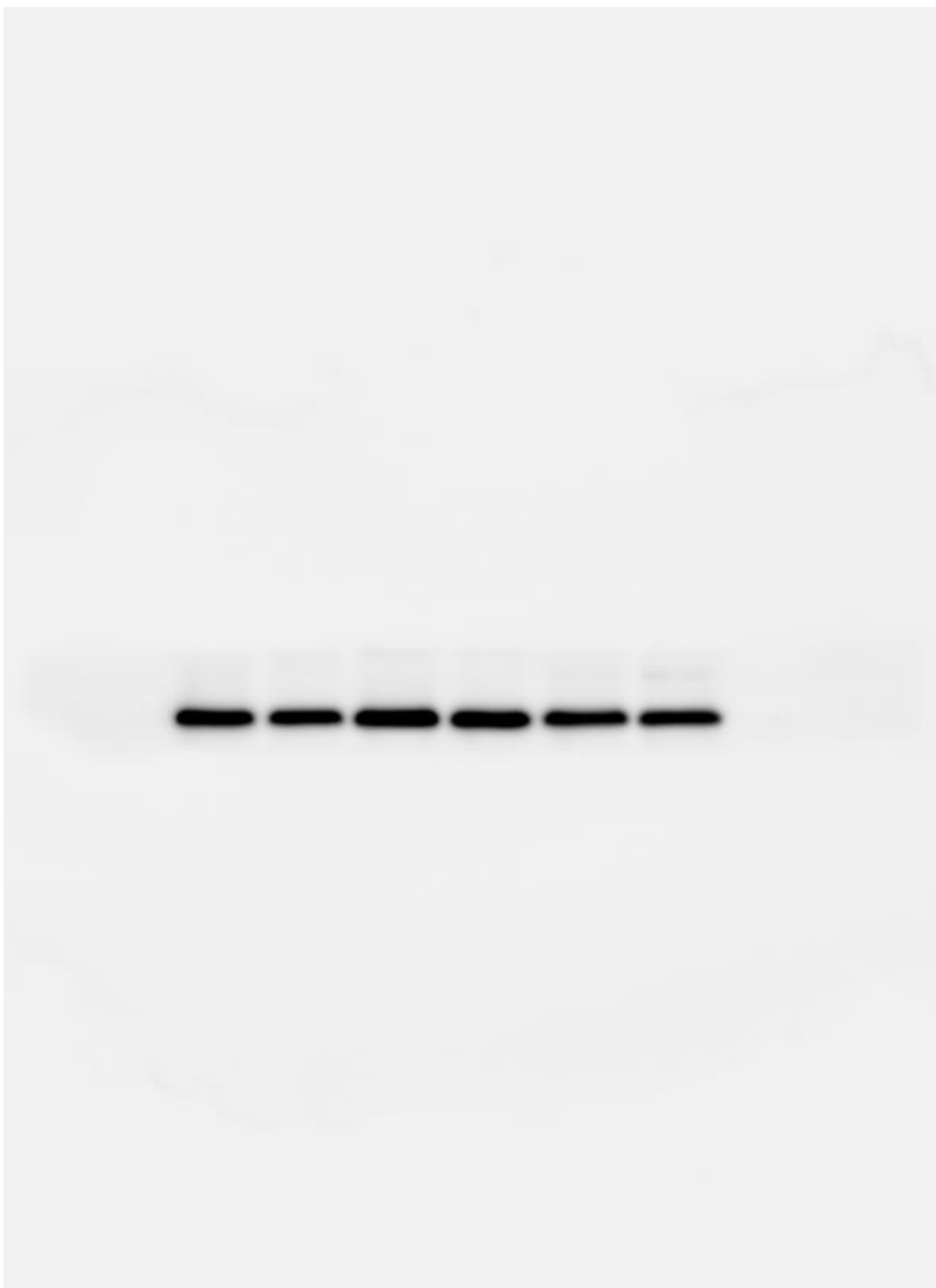

Figure S4. D

HMGA2

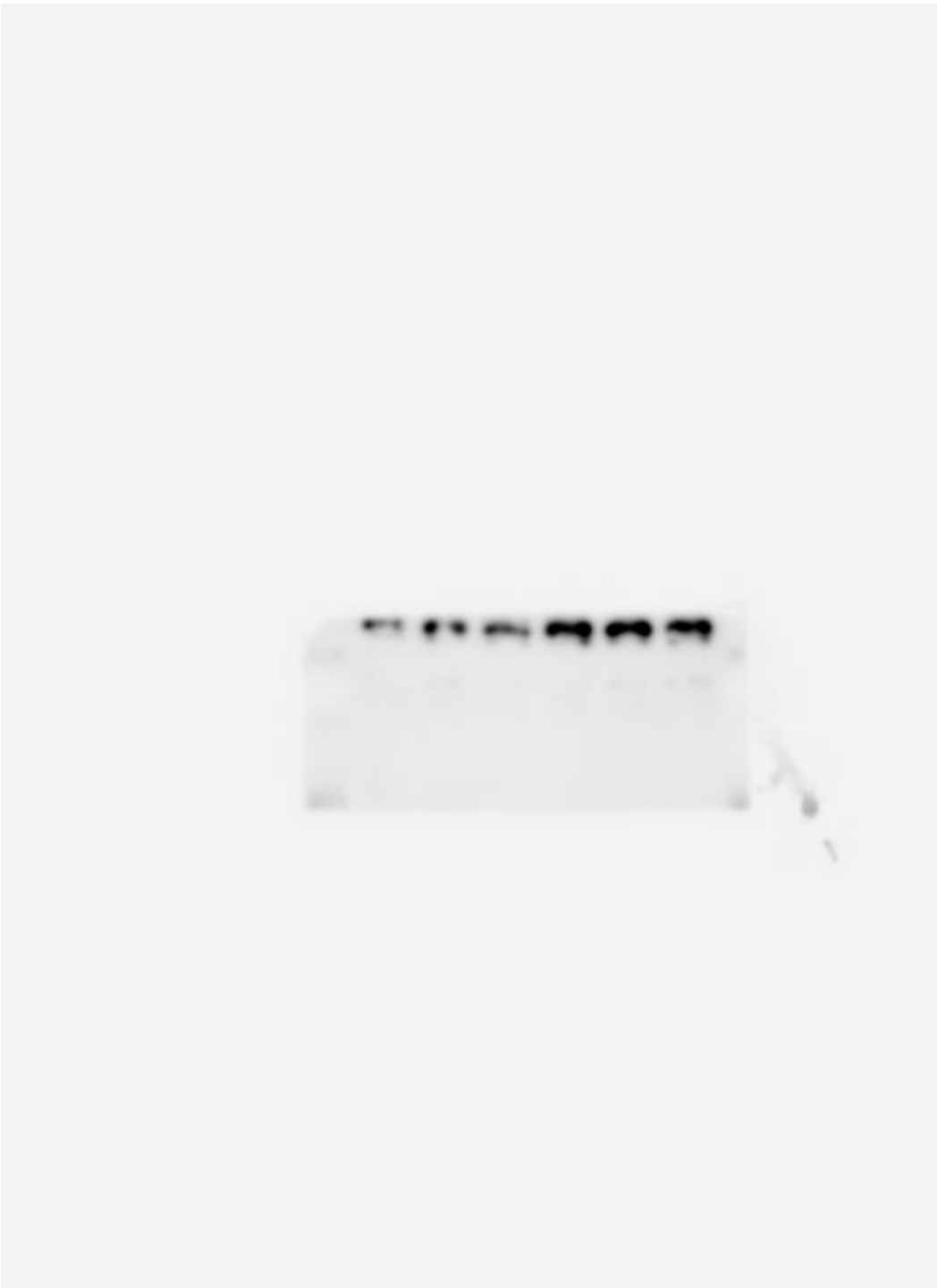

β-actin

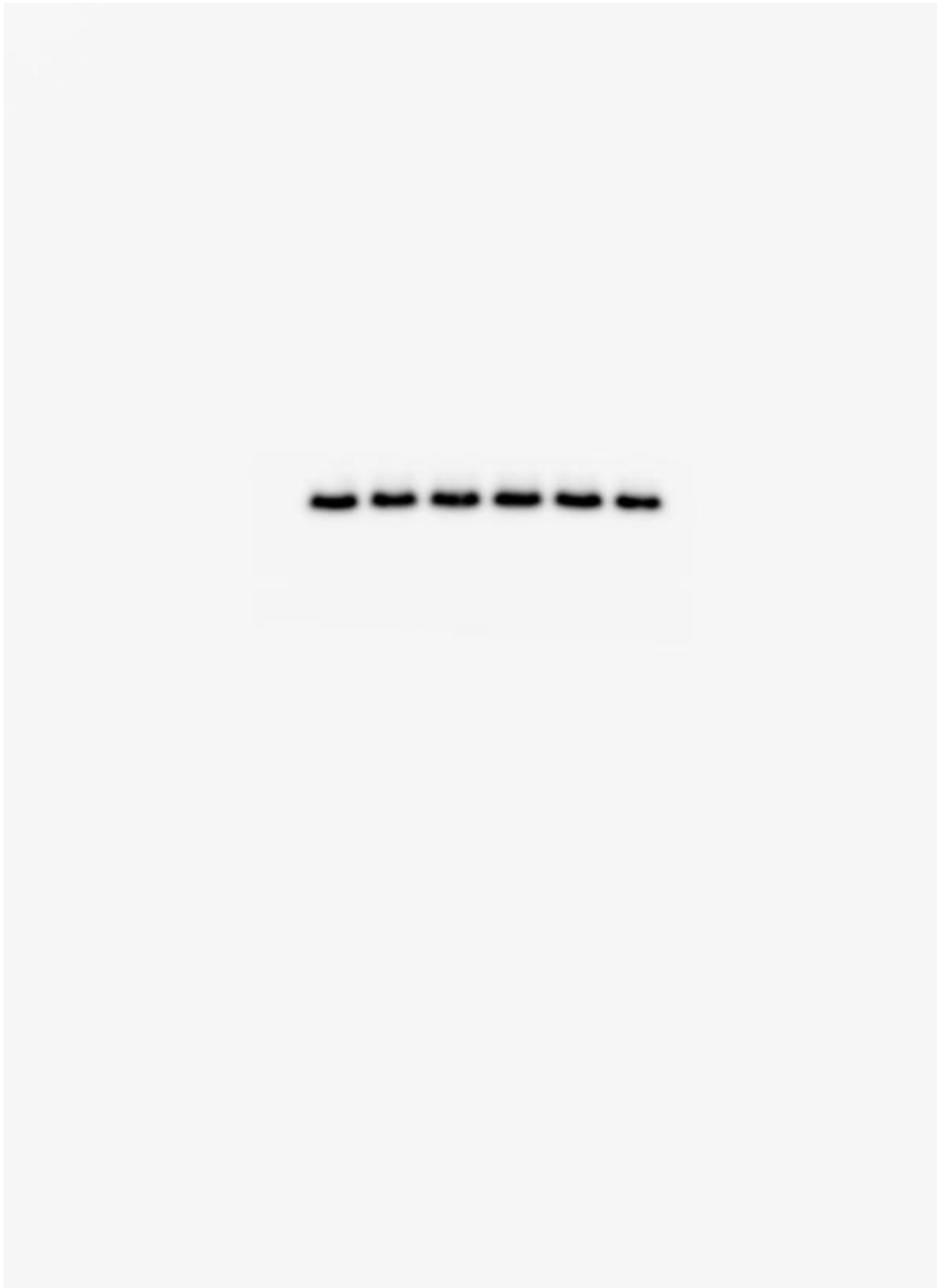

Figure S7. A

IGF2BP1

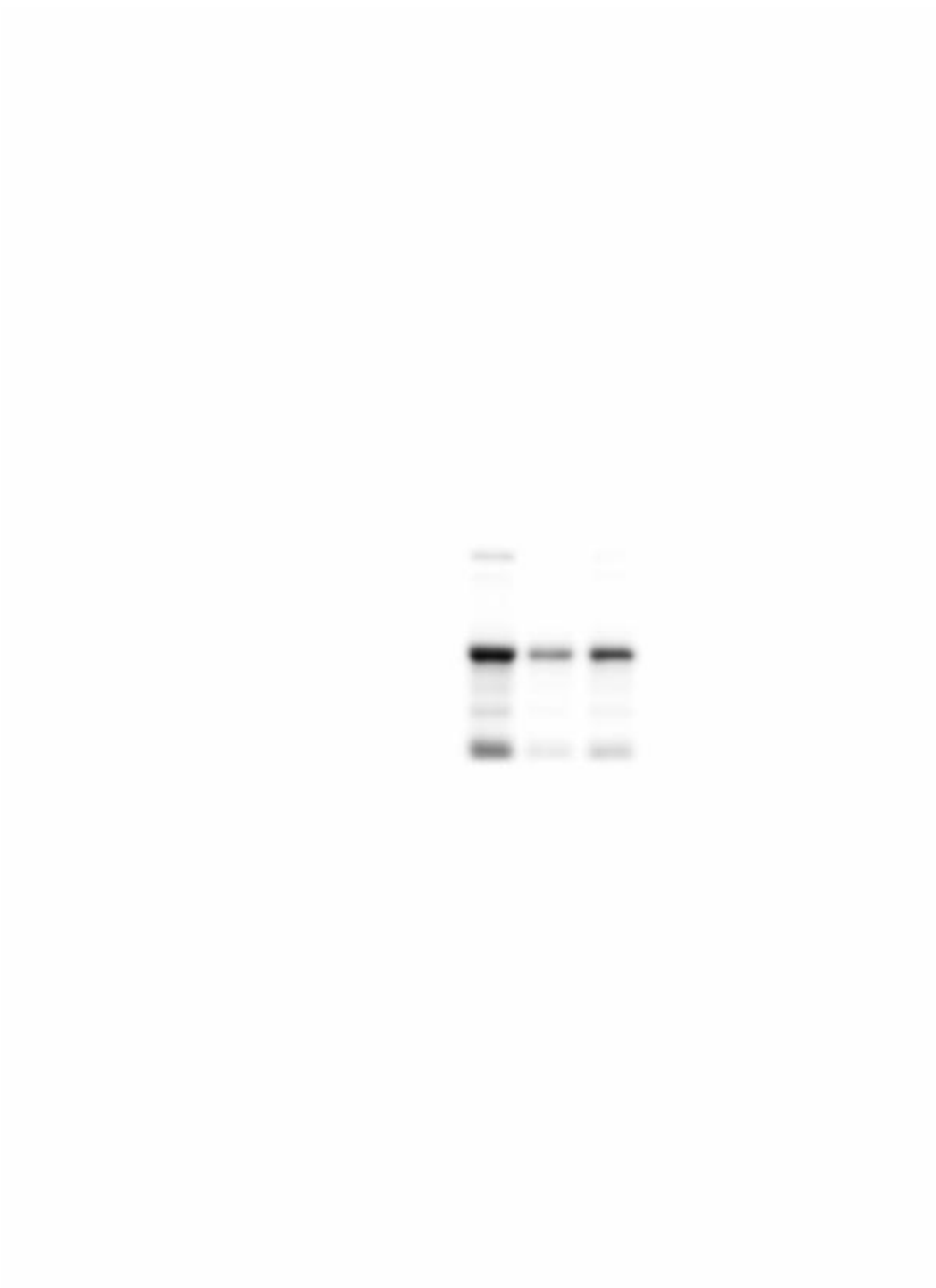

β-actin

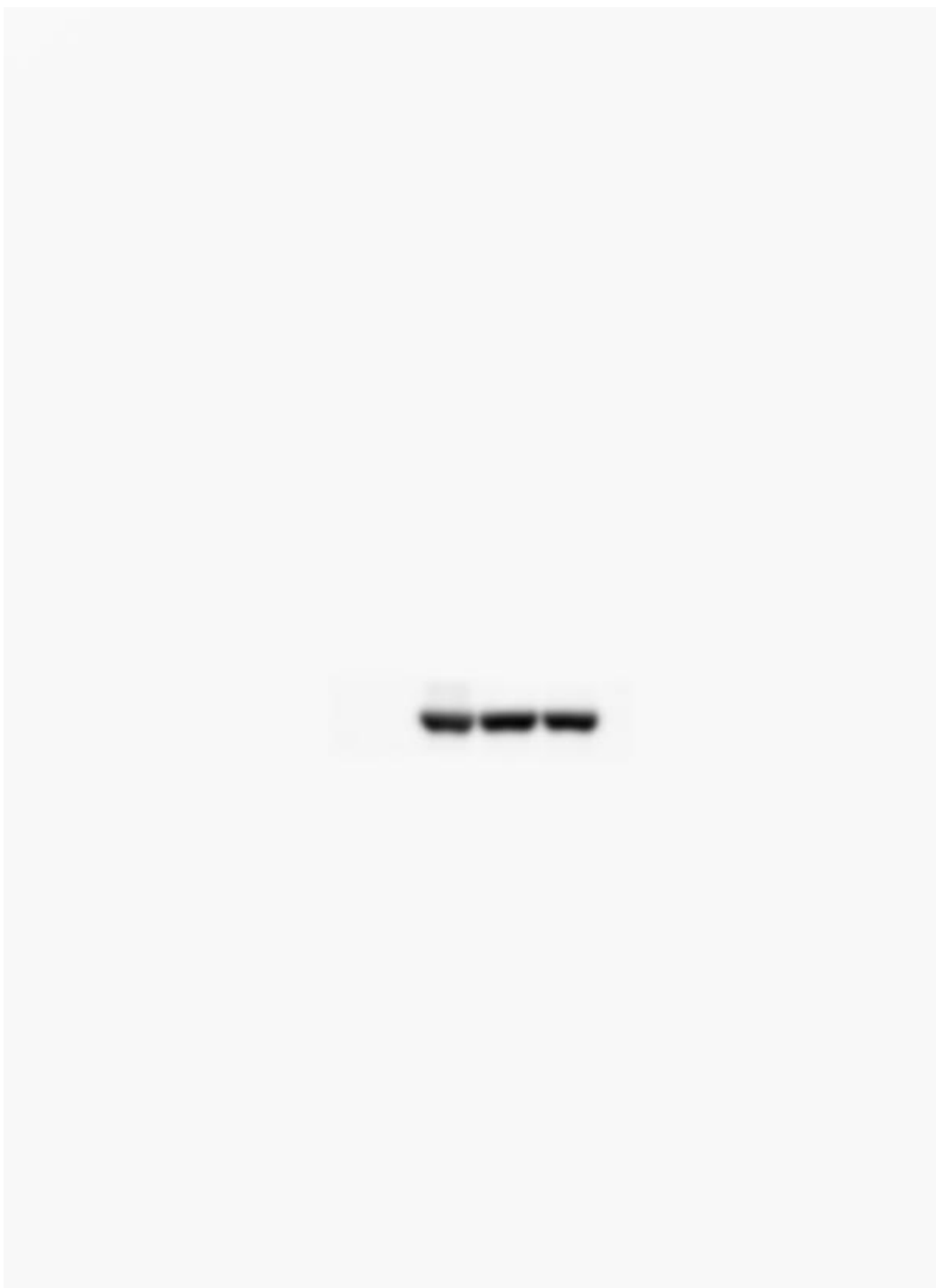

Figure S7. B

HMGA2 β-actin

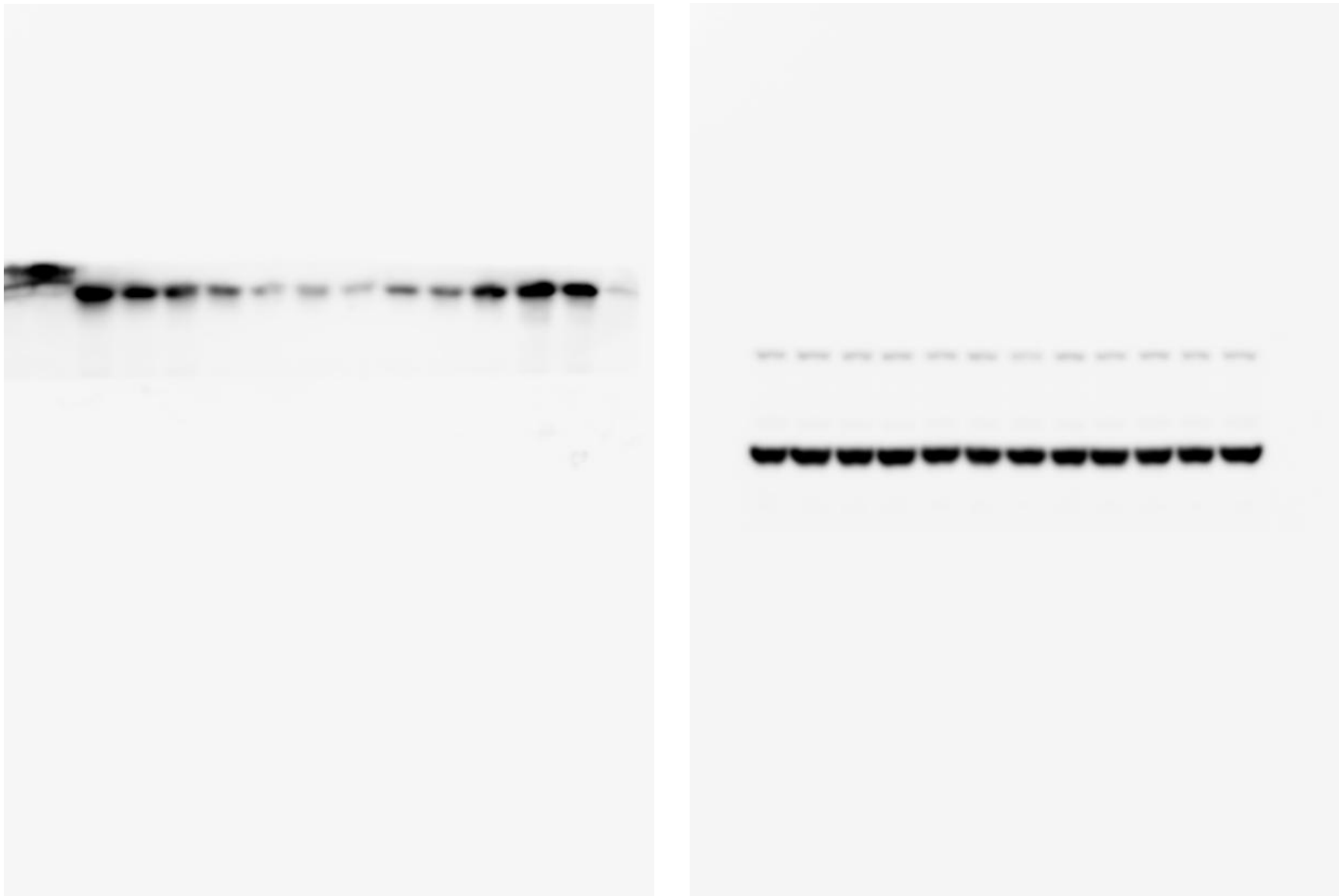

Supplement: Supplementary file 1 [file mmc1.pdf]
